# Spatially Separated Redox Centers in One‐Dimensional Sp^2^‐Carbon Covalent Organic Frameworks Enable Synergistic Photocatalytic Palladium Recovery and Bisphenol A Mineralization

**DOI:** 10.1002/advs.75468

**Published:** 2026-05-01

**Authors:** Yi‐Ru Chen, Jing‐Yi Li, Yao Xiao, Dan Zhong, Lu Zhang, Yu‐Ting Xie, Xiu Wang, Yibao Li, Wei‐Rong Cui, Jian‐Ding Qiu

**Affiliations:** ^1^ College of Chemistry and Materials Gannan Normal University Ganzhou China; ^2^ School of Metallurgical Engineering Jiangxi University of Science and Technology Ganzhou China; ^3^ School of Chemistry and Chemical Engineering Nanchang University Nanchang China

**Keywords:** bisphenol A, covalent organic frameworks, one‐dimensional, photocatalytic, palladium recovery

## Abstract

The simultaneous recovery of precious metal and pollutant degradation is often hindered by competing redox processes. Herein, we address this condition by designing a one‐dimensional covalent organic framework (COF), olefin‐linked iridium COF (Ole Ir‐COF^2+^), featuring a donor–acceptor–acceptor cascade. Ir complexes and pyrene units form a dual photosensitizing system enabling broad‐spectrum absorption, while quaternized bipyridine groups precisely modify the framework edges to create a cationic interface that selectively anchors PdCl_4_^2−^. The fully conjugated backbone establishes spatially separated oxidation (Ir/pyrene) and reduction (bipyridinium) centers, synergistically promoting efficient mass transfer and directional charge transport. Ole Ir‐COF^2+^ exhibits a carrier lifetime of 5.06 ns (threefold longer than Ole COF^4+^) and an ultralow exciton binding energy of 25.3 meV (half that of Ole COF^4+^). Without sacrificial agents, it achieves a palladium [Pd(II)] extraction capacity of 1749 mg g^−1^ (twofold higher than that of Ole COF^4+^) and a bisphenol A (BPA) degradation rate constant of 0.1710 min^−1^.

## Graphical abstract

A one‐dimensional sp^2^‐carbon covalent organic framework with spatially segregated redox centers integrates a dual photosensitizing system and a cationic interface for synergistic photocatalysis. The material achieves exceptional palladium recovery (1749 mg g^−1^) and bisphenol A mineralization (0.1710 min^−1^) without sacrificial agents, demonstrating efficient charge separation and practical applicability in real leachate.

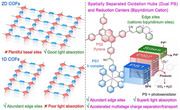

## 1 Introduction

The efficient recovery of the precious metal palladium (Pd) and deep purification of recalcitrant organic pollutants (e.g., bisphenol A, BPA) are critical demands for resource recycling and environmental remediation [1, 2, 3, 4, 5, 6, 7]. Currently, they are predominantly achieved using separate treatment processes in industries: precious metal recovery generally uses energy‐intensive pyrometallurgical or hydrometallurgical techniques, while organic pollutants are commonly degraded via advanced oxidation processes [8, 9, 10]. However, this conventional approach suffers from several drawbacks, including complex procedures, high costs, and the risk of secondary pollution. Photocatalysis technology offers a highly promising pathway for integrating resource recovery–environmental remediation by using solar energy to simultaneously facilitate precious metal reduction and organic pollutant oxidation [11, 12, 13, 14, 15, 16, 17]. However, achieving such a synergistic process imposes stringent requirements on the photocatalyst: it should be capable of simultaneously providing spatially separated active sites for efficient reduction and oxidation, and achieve effective separation and directional migration of photogenerated electron–hole pairs to their respective reaction centers [18, 19]. If these two aspects cannot be simultaneously achieved, the two competing pathways of Pd(II) reduction and BPA oxidation cannot proceed efficiently and synergistically, resulting in overall poor performance.

Among various candidate materials, covalent organic frameworks (COFs) are considered an ideal platform for overcoming the aforementioned bottlenecks owing to their customizable pore structures, tunable electronic energy levels, and extended π‐conjugated systems [20, 21, 22, 23, 24]. However, COFs with different dimensions exhibit inherent performance trade‐offs: two‐dimensional (2D) COFs possess well‐defined planar conjugated structures, facilitating photogenerated exciton migration and light harvesting; however, their tight interlayer π–π stacking severely limits the accessibility of active sites and reactant mass transfer [25, 26, 27, 28]. In contrast, one‐dimensional (1D) COFs can promote interfacial reactions by exposing abundant edge active sites, but their limited conjugation dimension tends to result in severe photogenerated charge recombination and weak light absorption capability [29, 30, 31, 32, 33, 34]. Even in traditional donor–acceptor (D–A) type COFs [35, 36, 37], although bulk charge separation is improved, their oxidation and reduction sites remain close at the molecular scale, triggering carrier recombination and competition for catalytic sites at the interface. The inherent contradiction between dimension and function renders the development of a novel structure that can overcome structural limitations and synergistically integrate efficient light harvesting, with highly accessible active interfaces, and directional charge transport. This is a critical scientific challenge that needs to be urgently resolved.

**SCHEME 1:**
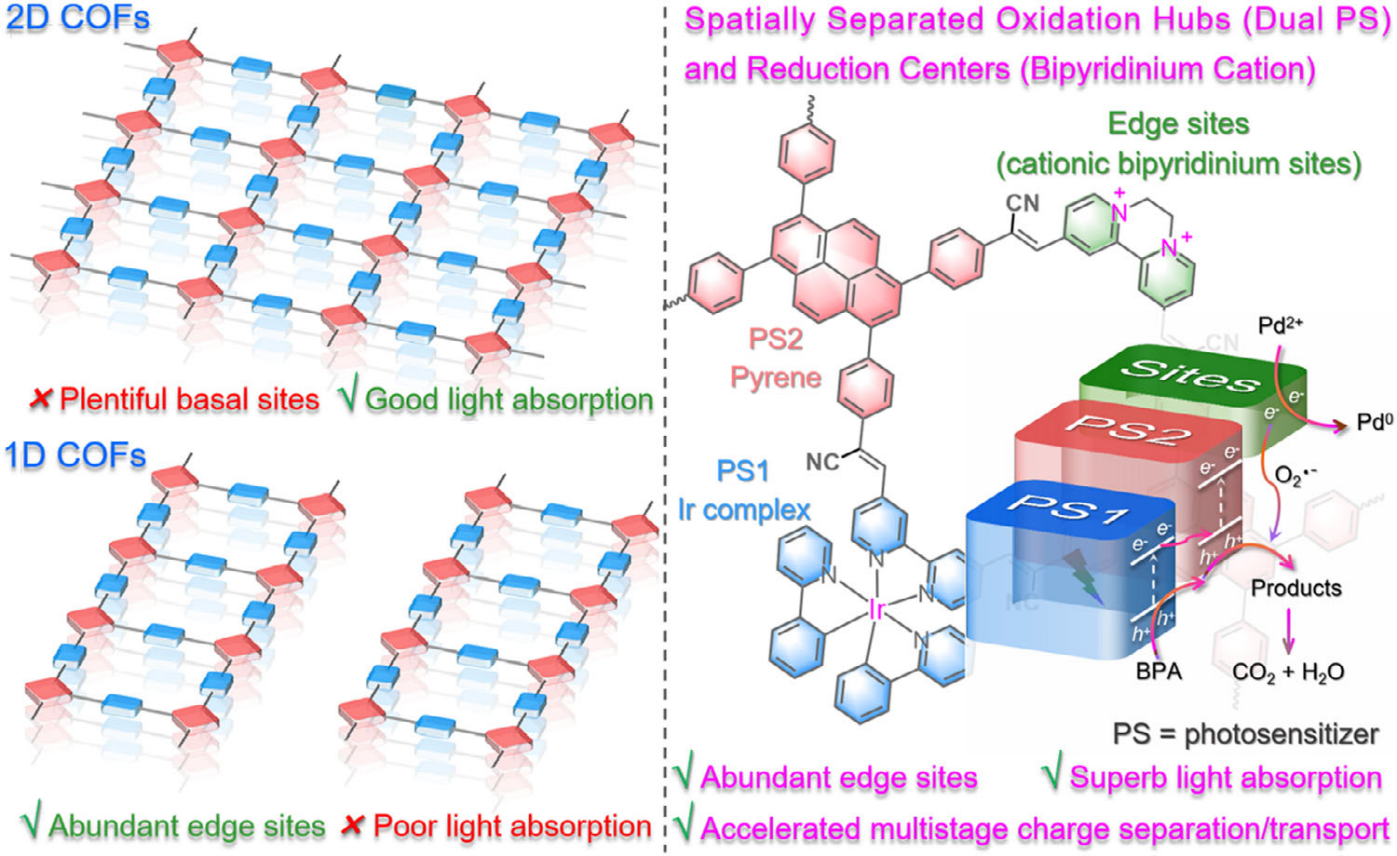
Overcoming the dimension‐function trade‐off in conventional COFs: synergistic photocatalytic BPA oxidation and Pd(II) reduction enabled by the spatially separated redox centers in 1D Ole Ir‐COF^2+^.

To address the core challenges of low reaction efficiency caused by the aforementioned dimensional–functional trade‐off and competition for redox sites, this study proposes and validates a strategy based on a D–A–A cascade structure to achieve spatially separated redox centers, successfully constructing a 1D fully conjugated COF (Ole Ir‐COF^2+^) (Scheme 1). This design integrates Ir complexes and pyrene units at the molecular level to construct a dual photosensitizing system for broad‐spectrum absorption. A highly accessible cationic interface was designed to selectively anchor palladium chloride (PdCl_4_^2−^) by precisely modifying the framework edge with bipyridine quaternary; subsequently, the fully conjugated backbone is leveraged to enable efficient and directional electron transport. Experimental characterization and theoretical calculations confirm that this ternary architecture successfully establishes spatially separated active centers: the Ir complex and pyrene unit serve as oxidation centers, inducing BPA degradation, while bipyridine quaternary ammonium salt captures and reduces Pd(II) by acting as a reduction center. This unique spatial separation characteristic, facilitated by the strong built‐in electric field resulting from the D–A–A energy level alignment, ensures that, following their separation at spatially isolated redox sites, photogenerated electrons migrate efficiently and unidirectionally to the reduction center, while holes are directed toward the oxidation sites. In contrast, 2D frameworks often suffer from interlayer recombination effects and tortuous charge migration pathways. This 1D confinement effect suppresses charge recombination, yielding an extended carrier lifetime of 5.06 ns for Ole Ir‐COF^2+^, three times that of the reference material Ole COF^4+^.

Compared with traditional inorganic semiconductor photocatalysts (such as TiO_2_ and g‐C_3_N_4_), Ole Ir‐COF^2^^+^ exhibits three core advantages in structural design. Firstly, atomically precise spatial separation of active sites, overcoming the competitive recombination caused by the random distribution of redox centers in traditional materials. Secondly, 1D open channels and cation interfaces, providing Pd(II) accessibility and selectivity far superior to 2D layered materials. Thirdly, rapid charge transport mediated by a fully conjugated framework, with a carrier lifetime (5.06 ns) significantly better than most organic and inorganic photocatalytic systems. Owing to this synergistic design of directional charge flow and spatial separation of the reaction center, Ole Ir‐COF^2+^ achieves a high Pd(II) extraction capacity of 1749 mg g^−1^ (twice that of Ole COF^4+^ and 15‐fold that of graphitic carbon nitride (g‐C_3_N_4_) with a BPA degradation rate constant of 0.1720 min^−1^ (13‐fold that of g‐C_3_N_4_), without any sacrificial agents. Theoretical studies revealed that as a hole scavenger, BPA further enhances charge separation by consuming holes and substantially reduces the Pd(II) reduction barrier from 1.44 to 0.89 eV, kinetically promoting the overall reaction.

## 2 Results and Discussion

### 2.1 Rational Design and Structural Characterization

**FIGURE 1:**
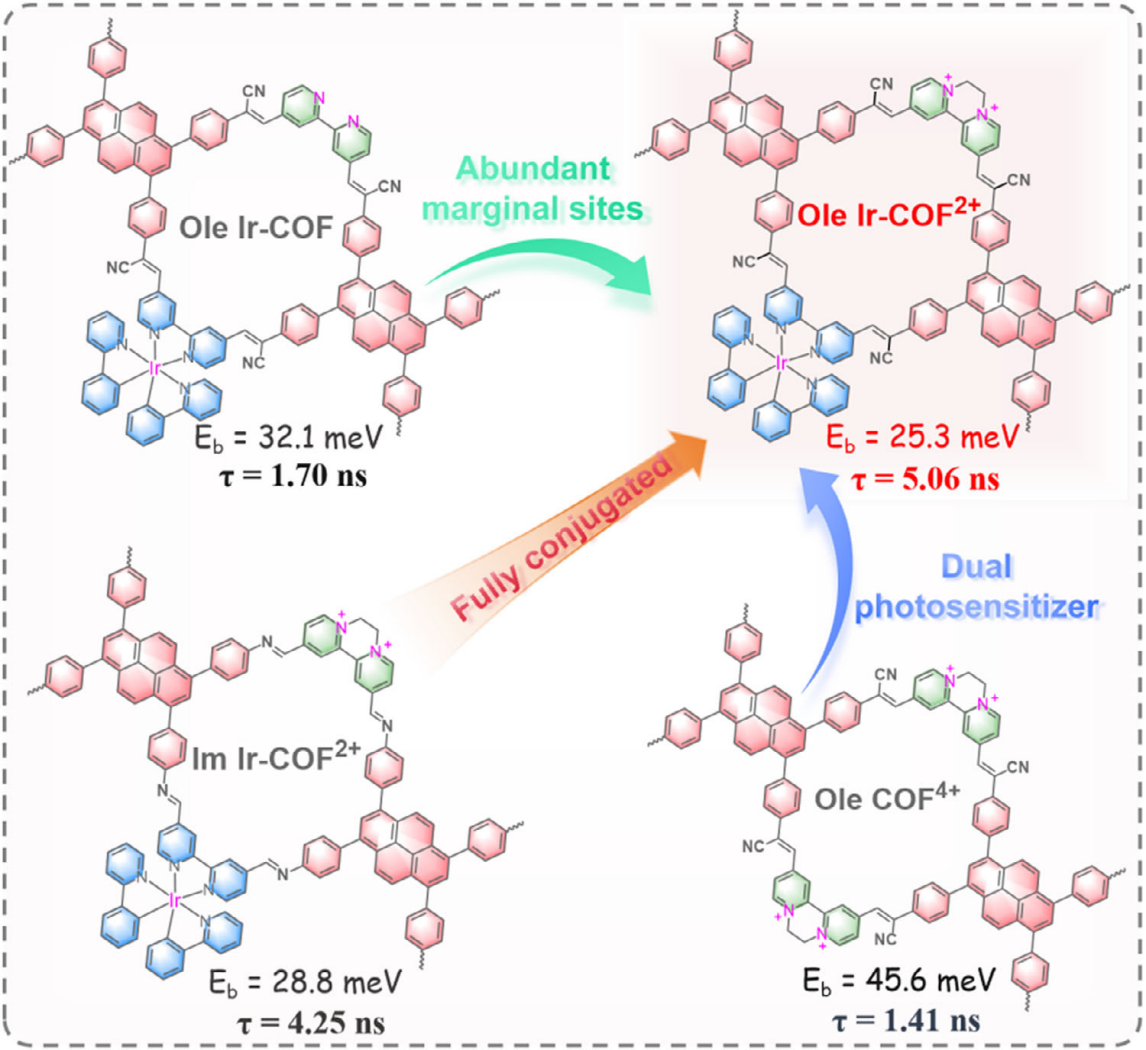
Comparative structure–property relationships of the four COFs. The D–A–A structured Ole Ir‐COF^2+^ exhibits optimal performance with a carrier lifetime (τ) of 5.06 ns and an exciton binding energy (E_b_) of 25.3 meV, outperforming its nonquaternized analog (Ole Ir‐COF), nondual‐photosensitizing COF (Ole COF^4+^), and the imine‐linked counterpart (Im Ir‐COF^2+^).

To achieve the synergistic photoreduction of precious metals and degradation of pollutants using COFs, a photocatalyst should be designed by integrating efficient light harvesting, accessible active interfaces, and directional charge transport. This study reports the synthesis of a fully conjugated 1D COF (Ole Ir‐COF; Figure 1 and Figure S1; See Supporting Information for synthetic details) from 2,2′,2′′,2′′′‐(pyrene‐1,3,6,8‐tetrayltetrakis(benzene‐4,1‐diyl))tetraacetonitrile (PyTT‐CN), (2,2′,‐bipyridine‐4,4′,‐dicarboxaldehyde)bis(2‐phenylpyridinato)iridium(III) (Ir(ppy)_2_(CHO)_2_), and 2,2′‐bipyridine‐4,4′‐dicarboxaldehyde (BpyA) via a Knoevenagel condensation reaction [32]. The relevant model reactions and synthetic routes of the organic building blocks are shown in Schemes S1–S3. To further enhance the accessibility of edge active sites, Ole Ir‐COF is modified via quaternization to obtain Ole Ir‐COF^2+^ [39, 40]. Its modification ratio is systematically optimized (Figures S2–S4 and Table S1) [41, 42, 43]. As a comparison, Ole COF^4+^ without the Ir complex was synthesized in parallel to verify the contribution of the introduced dual photosensitizing units (Ir complex and pyrene) to enhance the light‐harvesting ability of the framework (Figure S5) [44, 45, 46, 47]. Furthermore, Im Ir‐COF^2+^ with a similar structure was synthesized as a reference material to elucidate the advantages of fully conjugated olefin linkages over conventional imine linkages in charge transport performance (Figure S6). We successfully constructed a 1D fully conjugated Ole Ir‐COF^2+^ with a ternary cascade structure by integrating Ir complexes and pyrene units at the molecular level, establishing a dual photosensitizing system. We introduced highly accessible active sites by quaternizing the bipyridine at the backbone edge, creating an ideal platform for the efficient photoreduction of Pd(II) and synergistic BPA degradation.

**FIGURE 2:**
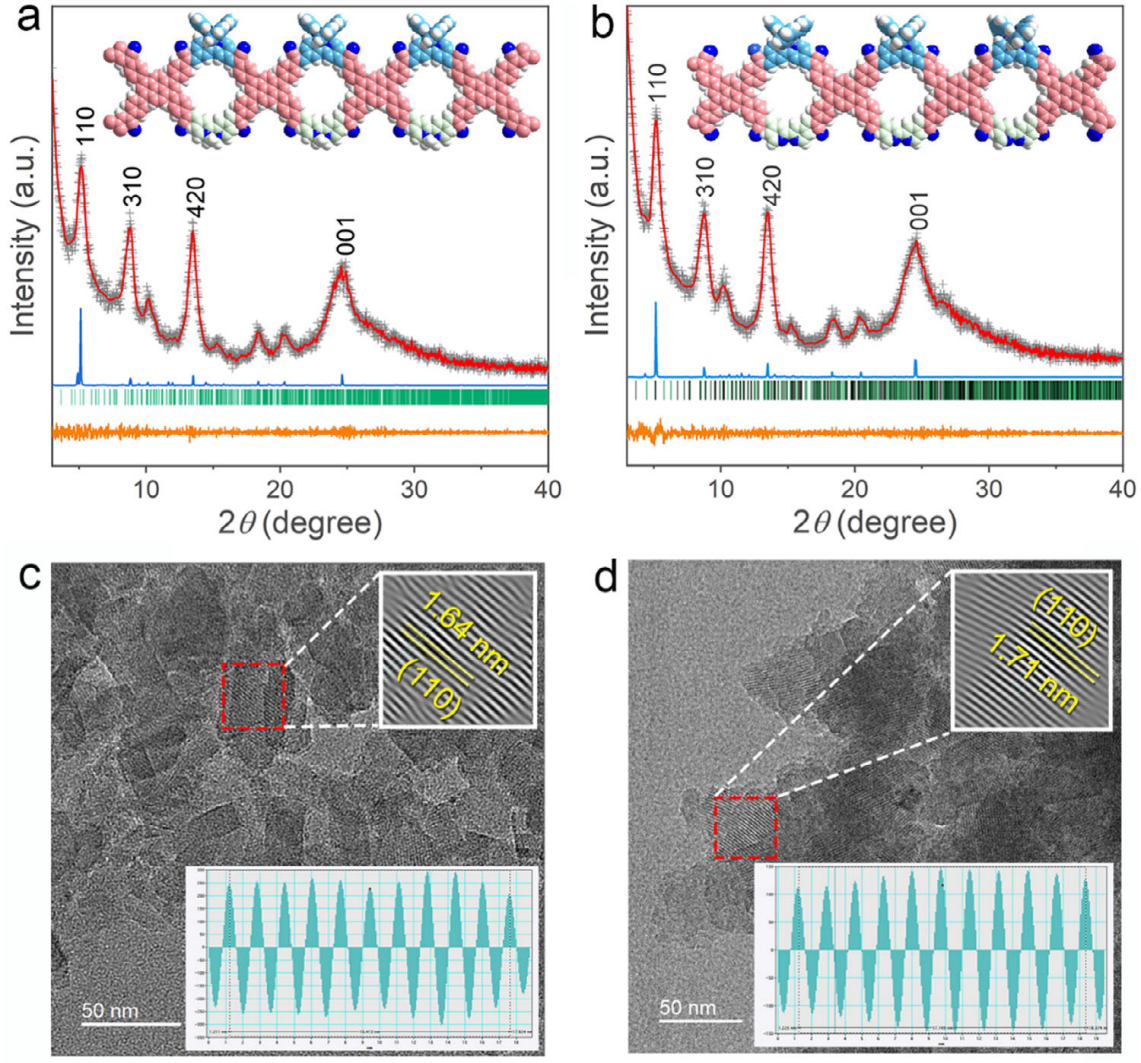
Crystal structure characterization of COFs. Experimental (black) and Pawley‐refined (red) PXRD patterns with simulated AA‐stacking structures for (a) Ole Ir‐COF^2^^+^, and (b) Ole Ir‐COF. Bragg positions (green), difference curves (orange), and AA‐stacking simulations (blue) are shown. Atomic colors in models: C (light blue/pink/mint), N (dark blue), H (white), Ir (purple). TEM images of (c) Ole Ir‐COF^2+^ and (d) Ole Ir‐COF, respectively.

The powder X‐ray diffraction (PXRD) pattern of Ole Ir‐COF^2+^ exhibits a strong diffraction peak at 5.2° (2θ), which corresponds to the (110) crystal plane (Figure 2a), with a series of higher‐order diffraction peaks at 8.8° (310), 10.2° (220), 13.5° (420), and 24.6° (001), indicating a highly ordered crystalline structure [48]. Pawley refinement results reveal that the experimental result aligns with the simulation profile based on the AA stacking model (R_p_ = 5.26% and R_wp_ = 6.84%), confirming a well‐defined 1D layered structure. The comparable PXRD patterns of the post‐modified Im Ir‐COF^2+^ and Ole COF^4+^ with the unmodified Ole Ir‐COF confirm the retention of the structural order, following quaternization (Figure 2b; Figures S7 and S8). Notably, the (001) peak at the 2θ value of 24.5° shows a broad full width at half maximum, originating from the enlarged intramolecular torsion induced by cationic functionalization. This suppressed interlayer π–π stacking potentially enhances the accessibility of active sites. High‐resolution transmission electron microscopy (HR‐TEM) images further confirm the high crystallinity of the four COFs (Figure 2c,d; Figure S9). The absence of lattice fringes characteristic of metallic nanoparticles or oxides in the Ir‐containing COFs confirms the atomic dispersion of Ir species and structural homogeneity.

**FIGURE 3:**
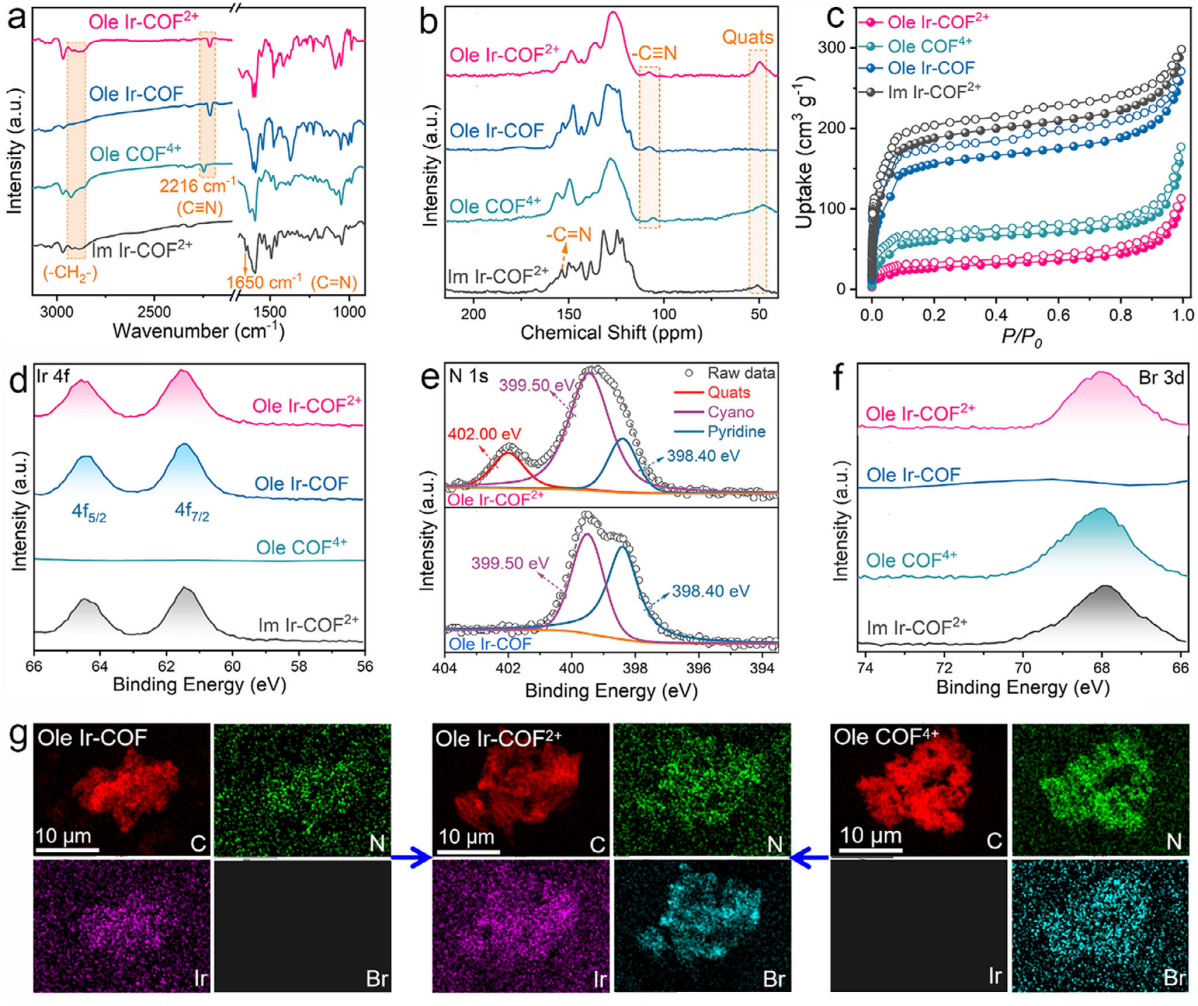
Structural and compositional authentication of the COFs. (a) FT‐IR spectra. (b) Solid‐state ^13^C CP/MAS NMR spectra. (c) Nitrogen adsorption‐desorption isotherms. (d) The high‐resolution XPS spectra of Ir 4f spectra of COFs. (e) The N 1s XPS spectra of Ole Ir‐COF^2+^ and Ole Ir‐COF. (f) The Br 3d XPS spectra of COFs. (g) EDS element mapping images of Ole Ir‐COF, Ole Ir‐COF^2+^, and Ole COF^4+^.

Fourier transform infrared (FTIR) spectra show the complete disappearance of the C═O stretching vibration peak (1703 cm^−1^) and the emergence of a strong vibration peak ascribed to C═C (1660 cm^−1^) (Figure 3a), indicating that Ole Ir‐COF^2+^ is highly condensed. Notably, characteristic aliphatic C–H stretching vibration bands (2928 and 2861 cm^−1^) are present in Ole Ir‐COF^2+^, Ole COF^2+^, and Im Ir‐COF^2+^, but absent in Ole Ir‐COF, confirming the successful introduction of alkyl groups (Figures S10–S12). FTIR spectra of Im Ir‐COF^2+^ show a C═N stretching vibration (1650 cm^−1^), confirming the formation of the imine linkage (Figure S13). Solid‐state carbon (^13^C) cross‐polarization magic‐angle spinning nuclear magnetic resonance spectra (Figure 3b; Figure S14) further elucidate the successful synthesis of COFs, with signals at 50 and 108 ppm for Ole Ir‐COF^2+^ and Ole COF^4+^, corresponding to alkyl and cyano groups, respectively, and the signal at 154 ppm for Im Ir‐COF^2+^ corresponds to imine carbons [25].

The porosity of COFs was systematically evaluated through 77 K nitrogen (N_2_) adsorption–desorption experiments (Figure 3c; Figure S15). Ole Ir‐COF and Im Ir‐COF^2+^ demonstrate high Brunauer–Emmett–Teller (BET) surface areas of 532 and 612 m^2^ g^−1^, respectively. Their pore size distribution is ∼1.30 nm, obtained using the nonlocal density functional theory (NLDFT; Figure S16), which is consistent with the model. The high surface area of Im Ir‐COF^2+^ can be attributed to the dynamic reversible nature of imine bonds, which promotes self‐healing of the framework [49]. The reduced specific surface areas and porosity of Ole Ir‐COF^2+^ and Ole COF^4+^ (Table S2) are attributed to the packing disorder caused by ion repulsion and steric hindrance of the Ir complex.

The evolution of N/bromine (Br) chemical states in COFs was analyzed via X‐ray photoelectron spectroscopy (XPS). High‐resolution Ir 4f spectra of Ole Ir‐COF^2^^+^, Ole Ir‐COF, and Im Ir‐COF^2^^+^ exhibit two characteristic peaks, corresponding to Ir 4f^7/2^ and Ir 4f^5/2^ with binding energies of 61.5 and 64.5 eV (Figure 3d), respectively. The high‐resolution N 1s spectra consistently show quaternary ammonium nitrogen (ca. 402.0 eV) across all cationic COFs, indicating the cyclic bis‐quaternization pathway (Figures S17–S19 and Table S3) [40, 42]. Ole Ir‐COF^2+^ distinctly exhibits additional peaks that can be attributed to pyridinic (398.4 eV) and cyano (399.5 eV) nitrogen (Figure 3e). The Br 3d spectra of Ole Ir‐COF^2+^, Ole COF^4+^, and Im Ir‐COF^2+^ exhibit a symmetric single peak at 67.6 eV, characteristic of Br^−^ (Figure 3f) [42]. Energy‐dispersive X‐ray spectroscopy (EDS) elemental mapping results (Figure 3g; Figure S20 and Table S4) confirm the homogeneous distribution of all constituent elements throughout the COF frameworks. In particular, Ir and Br are colocalized in Ole Ir‐COF^2^^+^, while Br is uniformly distributed in Ole COF^4+^ and Im Ir‐COF^2+^, fully corroborating the XPS findings.

Thermogravimetric analysis (TGA) was performed to further evaluate the stability of COFs. Figure S21 shows that the imine‐linked Im Ir‐COF^2+^ exhibits a 13.9% mass loss at 300°C; in contrast, the sp^2^c Ole Ir‐COF^2+^ merely lost 6.5%, indicating the superior thermal stability of the fully conjugated olefin skeleton. These results confirm the successful construction of a structurally ordered 1D sp^2^‐carbon COF (Ole Ir‐COF^2+^) by synergistically integrating dual photosensitizing with edge cationic sites. The ternary D–A–A cascade architecture of the material enables molecular‐scale spatial separation of oxidation (Ir complex/pyrene) and reduction (bipyridinium) centers, while its highly ordered channels and fully conjugated framework provide an ideal platform for directional charge migration and synergistic photocatalysis.

### 2.2 Ternary Cascade Architecture Enabling Superior Photophysics

**FIGURE 4:**
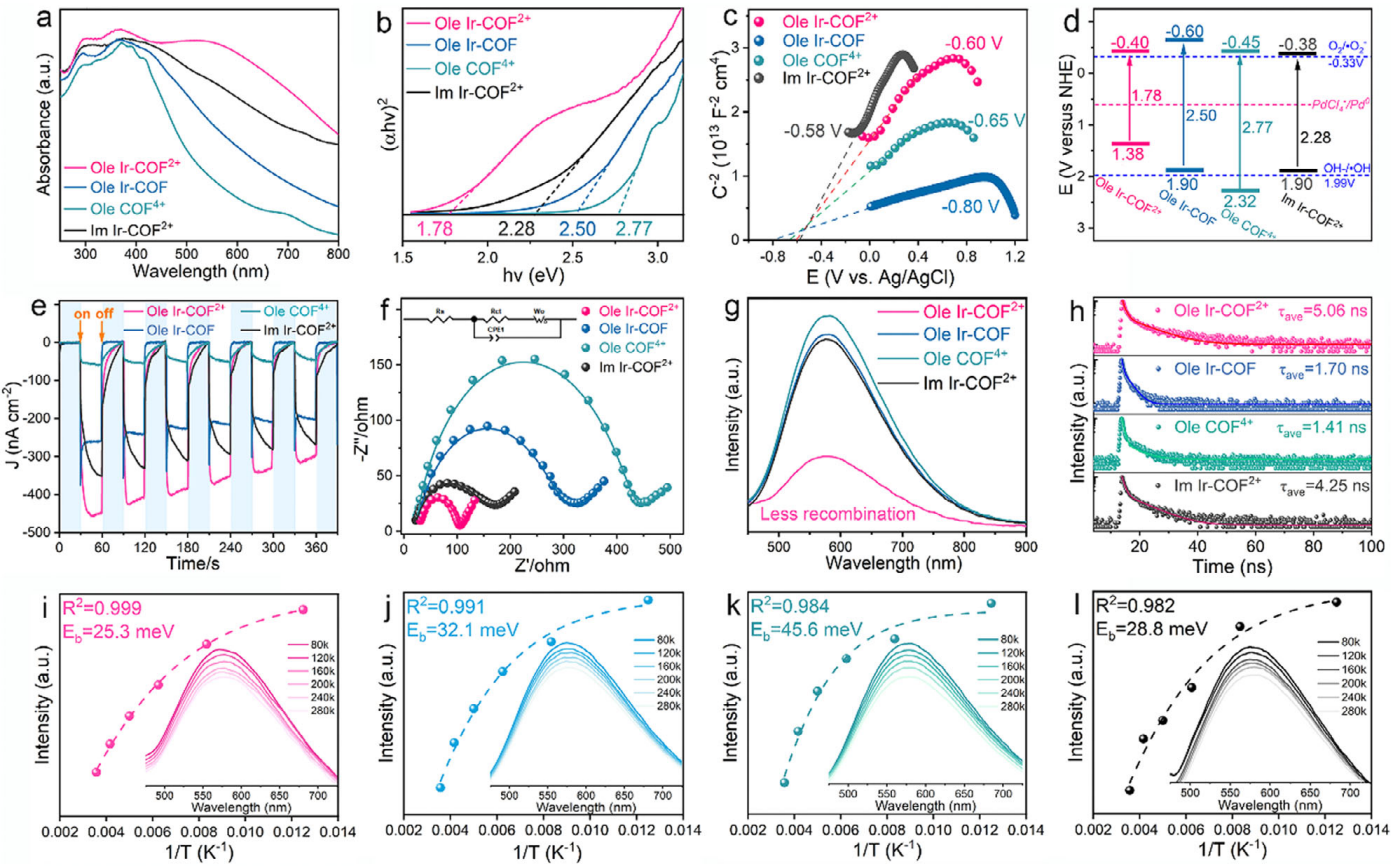
Rational design validated by optoelectronic performance. (a) UV–vis absorption spectra, (b) Tauc plot, (c) Mott–Schottky plot at 1 kHz frequency, and (d) band alignment diagram. (e) TPR spectra, (f) EIS spectra, (g) steady‐state PL spectra, (h) TR‐PL decay lifetimes spectra. TD‐PL spectra of integrated intensity as a function of temperature for (i) Ole Ir‐COF^2^^+^, (j) Ole Ir‐COF, (k) Ole COF^4^^+^, and (l) Im Ir‐COF^2^^+^ (Insets: TDPL spectra from 80 to 280 K).

We systematically investigated the photophysical properties of COFs using ultraviolet–visible (UV–vis) diffuse reflectance spectroscopy, transient photocurrent response (TPR) testing, and electrochemical impedance spectroscopy (EIS). While the UV–vis spectra reveal that Ole Ir‐COF^2+^ exhibits a broader photoresponse range in the near‐infrared (NIR) region (600–800 nm) than the other three COFs (Figure 4a). Tauc plot analysis further reveals that Ole Ir‐COF^2+^ exhibits the narrowest band gap among the four COFs (Figure 4b). These enhanced properties originate from the N^+^ center lowering the π→π*/n→π* transition energy, and the Ir‐induced enhancement of electron delocalization via metal d‐/ligand π‐orbital coupling. Mott–Schottky measurements (Figure 4c; Figure S22) indicate that the conduction band potentials (E_CB_) of four COFs are more negative than the standard redox potential of the O_2_/•O_2_^−^ pair [−0.33 V vs. normal hydrogen electrode (NHE)], providing the thermodynamic driving force for •O_2_^−^ generation. E_CB_ also fulfills the requirement for reducing PdCl_4_^2−^/Pd^0^ (0.62 V vs. NHE). Meanwhile, the sufficiently positive valence band potentials (E_VB_) drive the oxidative degradation of BPA (Figure 4d). Thus, COFs can effectively establish a photodriven bifunctional system, enabling the simultaneous reduction of Pd(II) and oxidation of BPA. The TPR and EIS further elucidated the kinetics of charge separation and migration. Among the four COFs, Ole Ir‐COF^2+^ exhibits the highest photocurrent density and smallest interfacial charge transfer resistance (*R*_ct_), confirming its superior carrier separation and migration kinetics (Figure 4e,f; Table S5), suggesting its potential for enhanced photocatalytic performance. This experimental phenomenon is in excellent agreement with theoretical calculations. Compared to Im Ir‐COF^2+^, Ole Ir‐COF^2+^ exhibits a lower charge reorganization energy (Figure S23), confirming that olefin bonds can effectively reduce energy loss during charge transport, thereby improving interfacial charge transfer efficiency.

Based on the aforementioned excellent photoelectric properties, we further investigated the separation efficiency of photogenerated carriers using photoluminescence (PL) spectroscopy, time‐resolved PL (TR‐PL), and temperature‐dependent PL (TD‐PL) spectroscopy. The lowest emission intensity of Ole Ir‐COF^2+^ reveals that its radiative recombination is effectively suppressed, while the nonradiative decay process dominates, which is conducive to carrier separation (Figure 4g). TR‐PL analysis reveals that Ole Ir‐COF^2+^ exhibits the longest τ (5.06 ns), which is 3‐, 3.6‐, and 1.2‐fold longer than Ole Ir‐COF (1.70 ns), Ole COF^4+^ (1.41 ns), and Im Ir‐COF^2+^ (4.25 ns), respectively (Figure 4h; Table S6). τ is prolonged owing to the suppression of nonradiative recombination, which provides additional time for carriers to migrate to the material surface and participate in the catalytic reaction, improving photocatalytic efficiency.

The results of the combined TD‐PL spectra (Figure 4il) and the Arrhenius equation show that the E_b_ of Ole Ir‐COF^2+^ (25.3 meV) is substantially lower than that of Ole Ir‐COF (32.1 meV), Ole COF^4+^ (45.6 meV), and Im Ir‐COF^2+^ (28.8 meV). This indicates that the D–A–A cascade structure and fully conjugated framework of Ole Ir‐COF^2+^ effectively reduces the exciton binding energy, kinetically promoting the generation and separation of free charges.

### 2.3 Exceptional Photocatalytic Pd(II) Extraction

**FIGURE 5:**
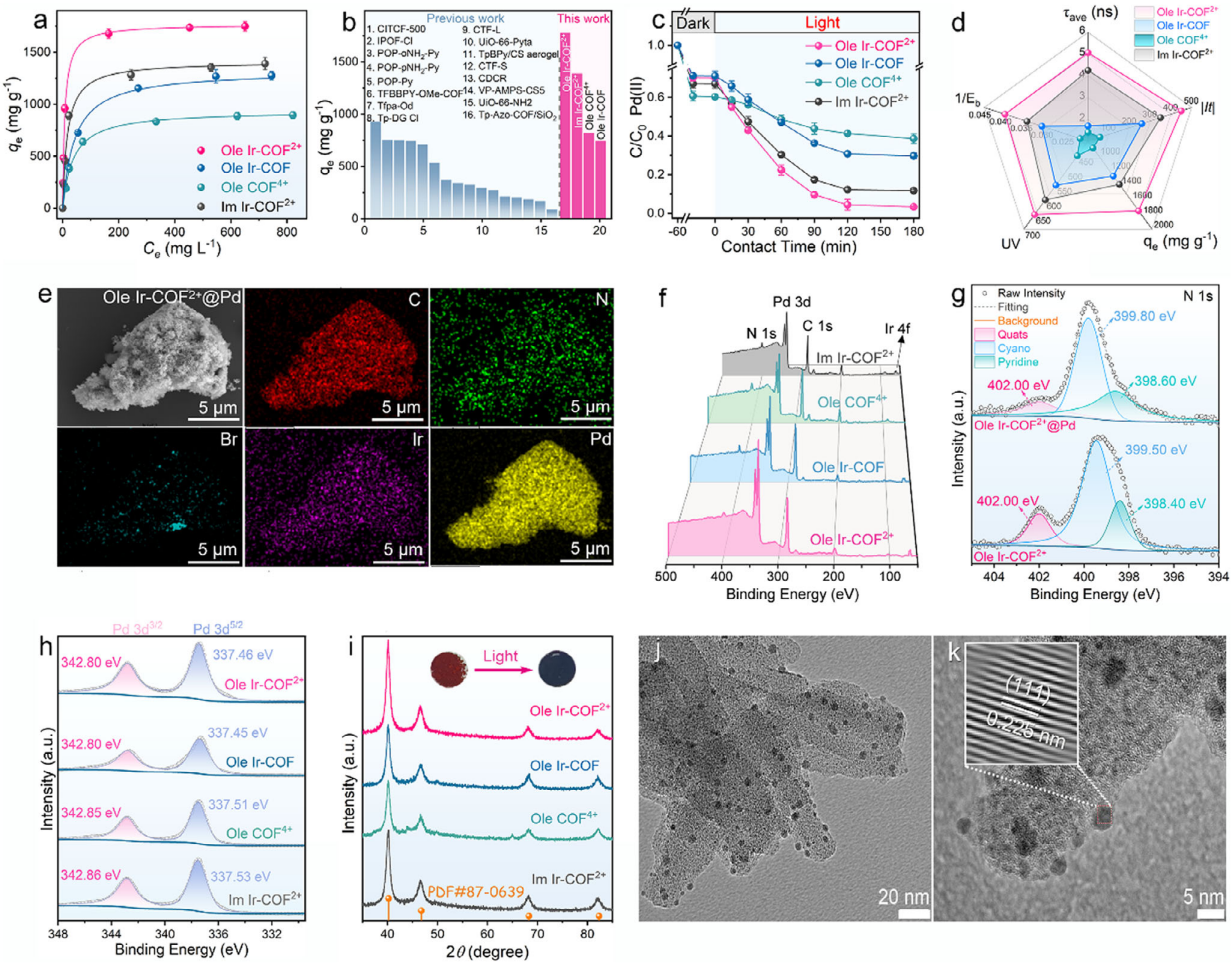
Exceptional photocatalytic Pd(II) recovery performance and mechanism. (a) Adsorption isotherm of PdCl_4_^2−^ on Ole Ir‐COF^2^^+^ (pink), Ole Ir‐COF (light blue), Ole COF^4^^+^ (light green), and Im Ir‐COF^2^^+^ (black) (pH 5.0). (b) Comparison of the adsorption performance for Pd(II) with different adsorbents. (c) Removal kinetics of PdCl_4_^2−^ on four COFs. (d) Radar chart of performance across all evaluated metrics. (e) SEM and EDS element mapping images of Ole Ir‐COF^2+^ after photocatalytic Pd(II) recovery (named Ole Ir‐COF^2^^+^@Pd). (f) Survey XPS spectra of Ole Ir‐COF^2^^+^ (pink), Ole Ir‐COF (light blue), Ole COF^4^^+^ (light green), and Im Ir‐COF^2^^+^ (black). The high‐resolution of (g) N 1s and (h) Pd 3d spectra. (i) PXRD profiles after photocatalytic Pd(II) recovery (insets: color evolution of Ole Ir‐COF^2^^+^). TEM images of Pd nanoparticles on the Ole Ir‐COF^2^^+^ at (j) 20 and (k) 5 nm.

Inspired by the exceptional optoelectronic properties of the aforementioned COFs, we systematically evaluated their performance for photoenhanced Pd(II) extraction. Subsequent experiments were conducted at an optimal pH of 5 [50, 51], which is consistent with the point of zero charge measured for Ole Ir‐COF^2+^ (Figures S24 and S25). Pd(II) adsorption capacities under dark conditions for Ole COF^4+^, Im Ir‐COF^2+^, Ole Ir‐COF^2+^, and Ole Ir‐COF are 502, 394, 363, and 319 mg g^−1^ (Figure S26), respectively, scaling positively with their quaternary ammonium salt content. Notably, the Pd(II) adsorption capacity of Ole COF^4+^ under dark conditions (502 mg g^−1^) is 1.8‐fold that of a comparable 2D COF (275 mg g^−1^) (Figures S27−S31), demonstrating the high accessibility of 1D cationic edge sites. Under illumination, Ole Ir‐COF^2+^ achieves a maximum Pd(II) extraction capacity of 1749 mg g^−1^, surpassing that of nonphotosensitized Ole COF^4+^ and benchmark g‐C_3_N_4_ by a factor of 2 and 15 (Figure 5a; Figures S32 and S33), respectively. Its Pd(II) extraction capacity sets a new record among all reported COFs to date and considerably surpasses those of advanced candidates, including Tfpa‐Od, Tp‐Azo‐COF–SiO_2_, and TFBBPY‐OMe‐COF (Figure 5b; Table S7). Notably, Ole Ir‐COF^2+^ achieved a Pd(II) removal rate of >99% within 2 h under illumination (Figure 5c), substantially outperforming the other three COFs and underscoring the pivotal role of its D–A–A cascade structure and conjugated framework in enhancing photocatalytic efficiency.

To evaluate its potential for precious metal recovery, the selective adsorption of Pd(II) by Ole Ir‐COF^2+^ was evaluated in simulated wastewater containing competitive ions, including Ni(II), Fe(II), Zn(II), Pb(II), Ca(II), Co(II), and Cd(II) (Figure S34). For Pd(II), Ole Ir‐COF^2+^ achieves a high distribution coefficient (K_d_ = 2.46 × 10^3^ mL g^−1^) combined with exceptional selectivity (SF = 400), outperforming crown ether‐based adsorbents (K_d_ = 0.0173 mL g^−1^) by orders of magnitude. Ole Ir‐COF^2+^ demonstrated exceptional stability for >5 cycles, with negligible loss in Pd(II) removal efficiency (96.9% retention; Figure S35). The superior photocatalytic Pd(II) extraction capability of Ole Ir‐COF^2+^ originates from the synergy of its optimal photophysical properties, including broad light absorption, extended charge carrier lifetime, low exciton binding energy, and superior photocurrent response, as visualized in the radar chart (Figure 5d).

EDS and XPS analysis results reveal a markedly intensified Pd signal under visible light irradiation (Figure 5e,f; Figures S36 and S37), corroborating the light‐enhanced Pd(II) extraction capability of Ole Ir‐COF^2+^ and further confirming the prior experimental findings. For Ole Ir‐COF^2+^@Pd, the N 1s binding energy substantially shifts to 398.6 eV (Δ = +0.2 eV) (Figure 5g; Figure S38 and Table S3) [39, 42, 52]. This shift can be ascribed to the strong negative electric field generated by the highly charged PdCl_4_^2−^ anion, which induces a highly pronounced polarization of the electron cloud around the quaternary nitrogen (N^+^) (Table S8). The superior photoenhanced extraction capability of Ole Ir‐COF^2+^ is unequivocally demonstrated by its considerably stronger Pd(II) signal under visible light irradiation than the other three COFs via XPS and EDS analyses (Figure 5h; Figures S36–S41) [2, 53, 54].

We monitored the chemical state evolution of Pd via XRD to further elucidate the reaction process. The sharp diffraction peaks at 40.1°, 46.7°, and 68.1° (2θ) closely match the (111), (200), and (220) planes of Pd^0^ (PDF #88‐2335; relative intensity ratio > 0.95), confirming the formation of Pd^0^ (Figure 5i; Table S9). The substantially higher intensity of Pd^0^ peaks in Ole Ir‐COF^2+^ than the other three COFs demonstrates its superior Pd(II) photoreduction capability. The successful reduction was further confirmed by a distinct color change of Ole Ir‐COF^2+^ from reddish‐brown to dark gray, offering macroscopic evidence of Pd^0^ formation (Insets in Figure 5i). TEM images further confirm the uniform dispersion of Pd^0^ nanoparticles on Ole Ir‐COF^2+^, with a measured lattice spacing of 0.225 nm (deviation < 0.003 nm), corresponding to the (111) plane of high‐purity crystalline Pd^0^ (Figure 5j,k; Figure S42). The superior photocatalytic Pd(II) reduction performance of Ole Ir‐COF^2+^ arises from its unique ternary cascade structure, where cationic edge sites facilitate Pd(II) enrichment and a conjugated dual photosensitizing system that enables efficient generation and migration of photogenerated electrons, which collectively drive the highly efficient extraction and photoreduction.

### 2.4 Synergistic Oxidation of BPA and Mechanistic Insights

**FIGURE 6:**
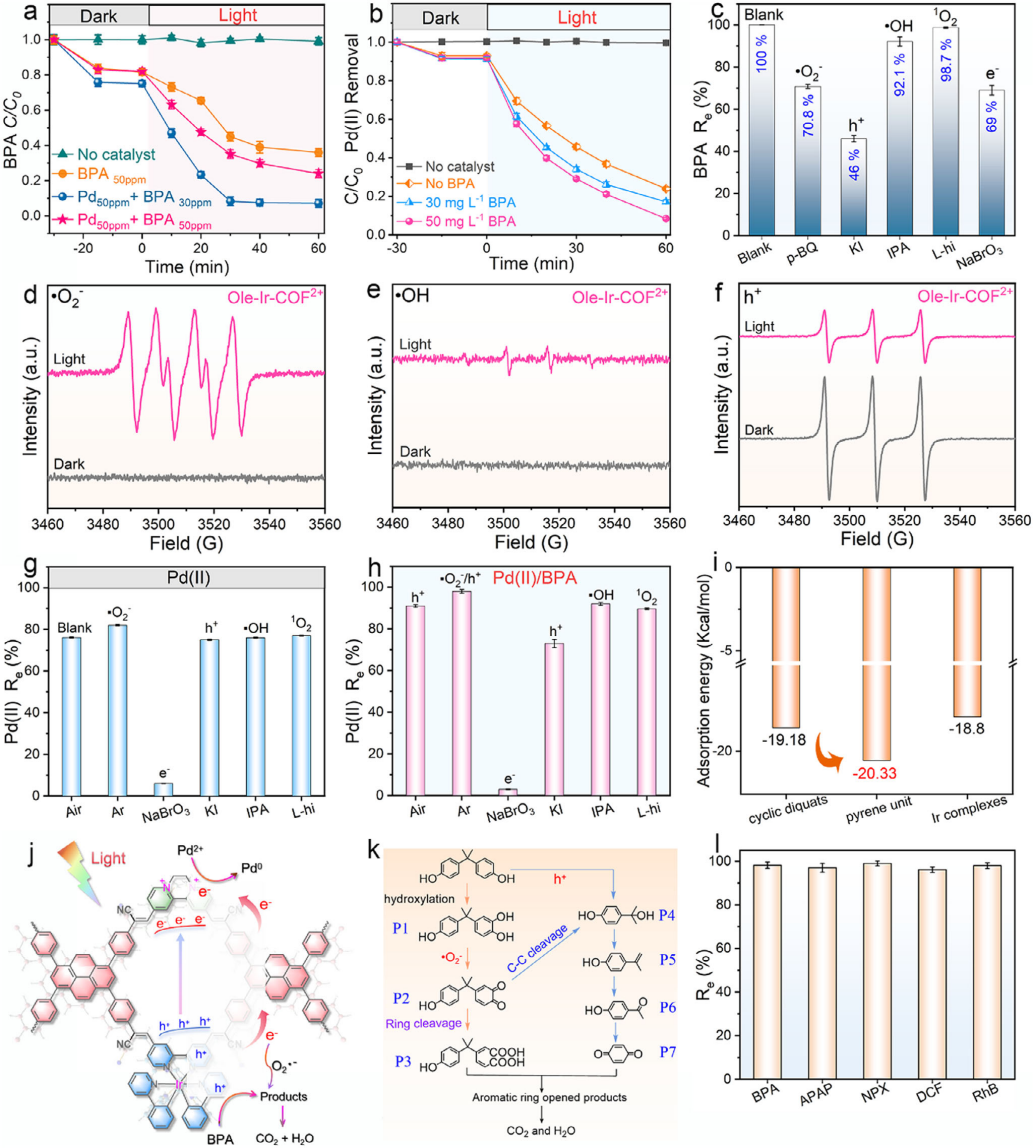
Synergistic catalytic mechanism and mutual promotion in the Pd(II)/BPA system. (a) BPA degradation kinetics with and without a catalyst or varied concentrations Pd(II). (b) Pd(II) reduction kinetics under Ole Ir‐COF^2^^+^ with different concentrations of BPA. (c) Effects of various scavengers on the degradation of BPA at a concentration of 50 mg L^−^^1^ in the absence of Pd(II). Spin‐trapping EPR spectra for reactive species in Ole Ir‐COF^2^^+^ with and without light: (d) •O_2_^−^, (e) •OH, (f) h^+^. Influence of different scavengers on the degradation of Pd (50 mg L^−^^1^) in the (g) absence and (h) presence of BPA. (i) Adsorption energies of BPA molecule adsorbed on Ole Ir‐COF^2+^ at three different sites. (j) Proposed degradation mechanism of the Pd(II)/BPA mixed system in the photocatalytic process mediated by Ole Ir‐COF^2^^+^. (k) Possible degradation pathways of BPA by Ole Ir‐COF^2+^ in Pd(II)/BPA system. (l) Photocatalytic degradation performance of Ole Ir‐COF^2+^ for four other emerging contaminants.

Having established the exceptional Pd(II) photoreduction capability of Ole Ir‐COF^2+^, we evaluated its complementary oxidation activity by implementing a coupled system for the concurrent reduction of Pd(II) and oxidation of BPA. Remarkably, under the same reaction conditions, Ole Ir‐COF^2+^ exhibits > 99% BPA degradation within 15 min, with a degradation rate constant 13‐fold higher than that of g‐C_3_N_4_ (Figure S43) [55]. Control experiments confirm the necessity of the catalyst, as BPA degrades negligibly without the catalyst (Figure 6a). The degradation efficiency of BPA (50 mg L^−1^) increases from 64% following 60 min of illumination in a single‐pollutant system to 76% in the presence of Pd(II), demonstrating a synergistic enhancement. Notably, this efficiency is further boosted to 93% when the initial BPA concentration is lowered to 30 mg L^−1^. This gradient enhancement confirms a synergistic effect between Pd(II) reduction and BPA oxidation half reactions, mediated by spatially separated redox centers. While Pd(II) reduction is constrained by inefficient charge separation in the absence of BPA, its kinetics are substantially promoted with the addition of BPA (50 mg L^−1^; Figure 6b). This confirms that BPA acts as an efficient hole scavenger, directing photogenerated electrons toward Pd(II) reduction, establishing a synergistic redox enhancement mechanism.

Scavenger experiments were conducted in a pure BPA system (Figure 6c), using the degradation efficiency at 60 min as a baseline (100%) to elucidate the BPA degradation pathway. Following N_2_ purging and the addition of benzoquinone (p‐BQ) to quench superoxide radicals (•O_2_^−^), the degradation rate reduces to 70.8%, confirming that •O_2_^−^ participates in the partial oxidation process. The degradation efficiency is further suppressed to 46% upon quenching photogenerated holes with potassium iodide (KI), indicating that holes are responsible for a dominant 64% of the overall activity. The removal of hydroxide radicals (•OH) using isopropanol (IPA) slightly decreases to 92%, indicating limited •OH involvement. Electron paramagnetic resonance (EPR) spectroscopy results provide direct evidence of the generation of key reactive species. Under illumination, characteristic signals for DMPO‐•O_2_^−^ (a 1:1:1:1 quartet) and DMPO‐•OH (a weak 1:2:2:1 quartet) are detected (Figure 6d,e), confirming the production of •O_2_^−^ and •OH. Notably, the signal for singlet oxygen (^1^O_2_) is particularly intense, indicating a robust oxidative capacity (Figure S44). Crucially, Ole Ir‐COF^2+^ exhibits substantially stronger EPR signals for all three reactive species (•O_2_^−^, •OH, and ^1^O_2_) than the nondual photosensitizing Ole COF^4+^, directly correlating its superior D–A–A structure and dual‐photosensitizing system with enhanced photocatalytic activity. This enhanced generation of •O_2_^−^ was quantitatively verified using the nitroblue tetrazolium tests (Figure S45). The more pronounced attenuation of the TEMPO‐h^+^ signal in Ole Ir‐COF^2+^ under light irradiation provides direct evidence of its superior charge separation capability over Ole COF^2+^ (Figure 6f).

Photocatalytic Pd(II) removal was systematically assessed in single and binary (with BPA) systems under different atmospheric conditions with various scavengers (Figure 6g,h). In the single Pd(II) system, argon (Ar) purging increases the removal rate to 82%, substantially surpassing the 76.1% in air, by suppressing competitive O_2_ reduction and directing additional electrons to Pd(II). The crucial role of electrons is confirmed by adding sodium borate (NaBrO_3_), an electron scavenger, which drastically inhibits removal to 6%. In the Pd(II)/BPA system, Pd removal increases to 91% in air and further to 98% under Ar. This enhancement is caused by BPA acting as an efficient hole scavenger to promote charge separation. The synergistic combination of Ar (inhibiting •O_2_^−^) and BPA (consuming h^+^) establishes a dual regulatory mechanism that optimizes electron utilization for Pd(II) reduction. Theoretical calculations identify the pyrene unit as the preferred adsorption site for BPA, with a strong binding energy of −20.33 kcal mol^−1^ (Figure 6i; Figure S46), thereby facilitating its subsequent degradation. These findings support a synergistic mechanism (Figure 6j): photogenerated electrons reduce Pd(II) and partially convert O_2_ to •O_2_^−^, while holes oxidize BPA directly or indirectly (via •O_2_^−^) (Figure 6k; Figure S47) [56, 57, 58]. To evaluate the mineralization efficiency of BPA, the removal of total organic carbon (TOC) during the reaction was investigated. After 120 min of illumination, the TOC removal rates of the single BPA system and the Pd(II)+BPA system were approximately 45.3% and 89.1%, respectively, indicating that most of the BPA was completely mineralized, and the presence of Pd(II) promoted the conversion of BPA to the final product (Figure S48). Furthermore, Ole Ir‐COF^2+^ demonstrates broad‐spectrum activity, achieving removal rates up to 98% for various pollutants (acetaminophen, diclofenac, naproxen, and rhodamine B; Figure 6l; Figure S49 and Table S10), highlighting its potential to be used in water treatment. The structural stability of Ole Ir‐COF^2+^ after catalysis was verified by XPS and UV–vis spectroscopy measurements. The binding energies of Ir 4f and C 1s remained essentially unchanged relative to the fresh catalyst after reaction (Figures S50 and S51a–c and Table S11). In addition, its UV–vis spectral characteristics remained almost unchanged after catalysis (Figure S51d). These results confirm the structural stability of both the Ir complex and the pyrene unit after catalysis.

The experimental results demonstrate that Ole Ir‐COF^2+^ can efficiently generate sufficient photogenerated electron–hole pairs under illumination. Its unique D–A–A ternary cascade structure effectively promotes the spatially directional separation and migration of photogenerated carriers. Specifically, a portion of the photogenerated electrons directly reduces Pd(II) to Pd(0) at the cationic sites, while another portion reduces dissolved oxygen to generate •O_2_^−^ radicals. Subsequently, BPA undergoes nucleophilic attack by these •O_2_^−^ radicals, yielding intermediate products capable of efficiently scavenging holes, thereby indirectly promoting the reduction of Pd(II). Concurrently, the initially generated holes accumulate at oxidation sites to directly oxidize BPA, establishing an alternative degradation pathway for BPA. This spatial separation mechanism successfully achieves efficient coupling of Pd(II) reduction and BPA oxidation without a sacrificial agent.

### 2.5 Theoretical Elucidation of the Synergistic Mechanism

**FIGURE 7:**
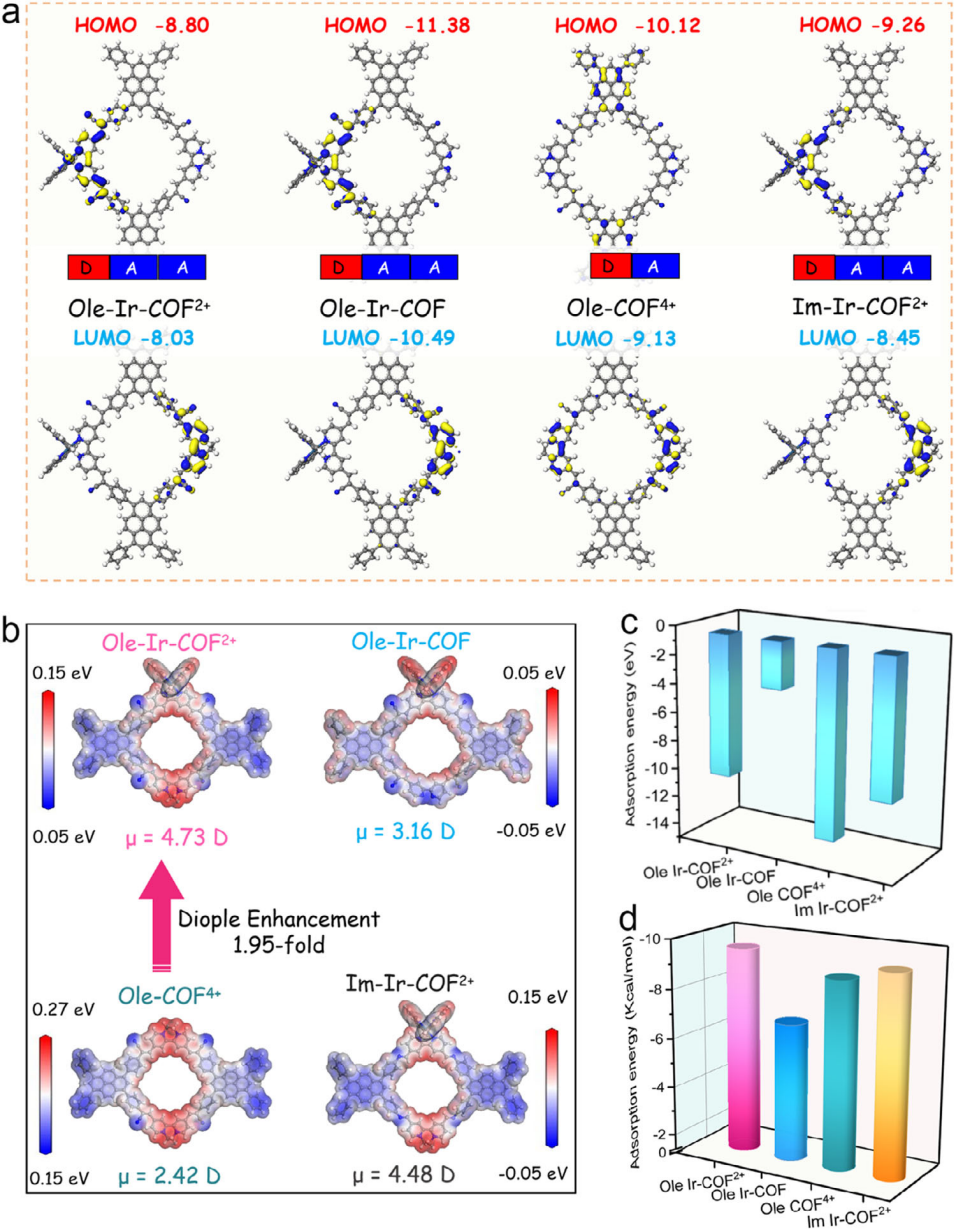
Theorical calculation. (a) HOMO and LUMO energy levels and (b) electrostatic potential surfaces for Ole Ir‐COF^2^^+^, Ole Ir‐COF, Ole COF^4^^+^, and Im Ir‐COF^2^^+^. (c) PdCl_4_^2^^−^ adsorption energies at bipyridine N‐sites of Ole Ir‐COF^2^^+^, Ole Ir‐COF, Ole COF^4^^+^, and Im Ir‐COF^2^^+^. (d) The adsorption potential energies between O_2_ and four COFs.

Density functional theory (DFT) calculations elucidate the electronic structures of COFs, revealing trends consistent with experimental observations (Figure 7). Ole Ir‐COF^2+^ exhibits the narrowest highest occupied molecular orbital–lowest unoccupied molecular orbital (HOMO–LUMO) gap (0.77 eV), attributable to its D–A–A cascade and fully conjugated framework, than 0.89 eV (Ole Ir‐COF), 0.99 eV (Ole COF^4+^), and 0.81 eV (Im Ir‐COF^2+^). Frontier orbital analysis confirms spatial charge separation, with the HOMO localized on Ir donors and the LUMO on pyrene–bipyridine acceptors (Figure 7a), aligning with the active sites for Pd(II) reduction and BPA oxidation and supporting a directed electron migration pathway. Quantitative dipole moment analysis showed that Ole Ir‐COF^2+^ exhibits the largest dipole (4.73 D), approximately 1.50‐, 1.95‐, and 1.06‐fold greater than Ole Ir‐COF, Ole COF^4+^, and Im Ir‐COF^2+^, respectively (Figure 7b). This enhanced polarity, originating from the D–A–A structure, facilitates exciton dissociation via a strong built‐in electric field [59]. Electrostatic potential and adsorption energy calculations identified bipyridinium units as strong Pd(II) adsorption sites (ΔG_ads_ = −12.59 eV for Ole COF^4+^; Figure 7c; Figure S52), which is consistent with experimental adsorption data. Furthermore, O_2_ adsorption energies reveal that Ole Ir‐COF^2+^ exhibits the strongest oxygen affinity (–9.56 kcal mol^−1^; Figure 7d; Figure S53), promoting O_2_ enrichment and electron transfer to generate •O_2_^−^ species.

**FIGURE 8:**
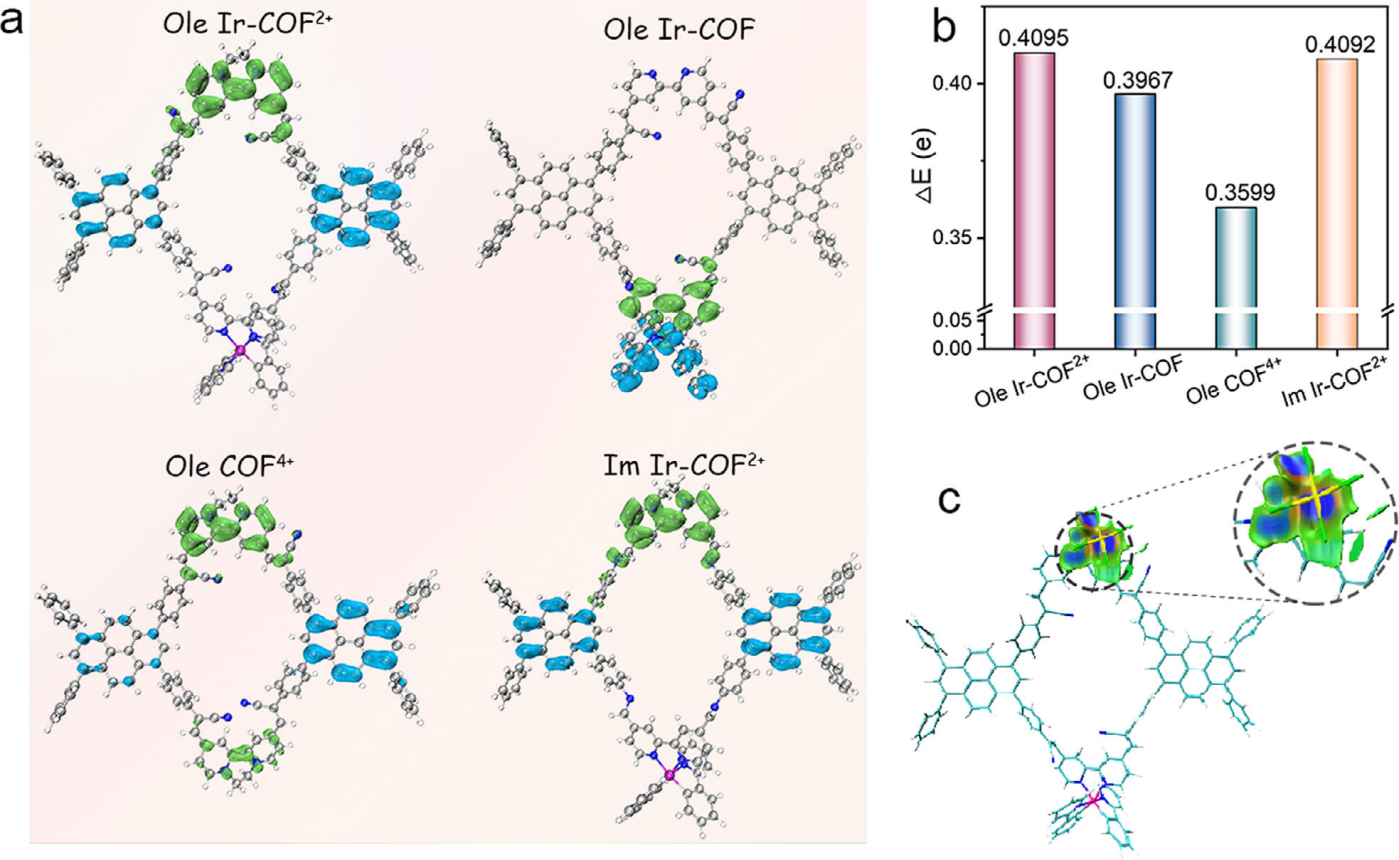
Visualizing spatial charge separation for catalytic enhancement. (a) Electron‐hole distributions of excited Ole Ir‐COF^2^^+^, Ole Ir‐COF, Ole COF^4^^+^, and Im Ir‐COF^2^^+^ in the presence of PdCl_4_^2−^; green and blue areas denote electron accumulation and depletion, respectively. (b) IFCT results for Ole Ir‐COF^2^^+^, Ole Ir‐COF, Ole COF^4^^+^, and Im Ir‐COF^2^^+^ with PdCl_4_^2−^. (c) IGMH analysis of Ole Ir‐COF^2^^+^; isosurfaces are colored by sign(λ_2_) ρ (δginter = 0.005 a.u.).

Excited‐state electron–hole distribution analysis reveals efficient spatial charge separation in the Ole Ir‐COF^2+^/PdCl_4_^2^^−^ system, with electrons delocalizing toward PdCl_4_^2−^ and holes localized on the pyrene units (Figure 8a) [60]. Interfragment charge transfer analysis confirms that Ole Ir‐COF^2+^ exhibits the largest electron transfer magnitude (ΔE) to PdCl_4_^2−,^ underscoring its superior charge transfer kinetics (Figure 8b). The independent gradient model analysis further identifies dominant electrostatic attraction, which surpasses van der Waals forces between the framework and PdCl_4_^2−^ (Figure 8c; Figure S54). This interaction induces an interfacial built‐in electric field that directionally promotes electron migration.

**FIGURE 9:**
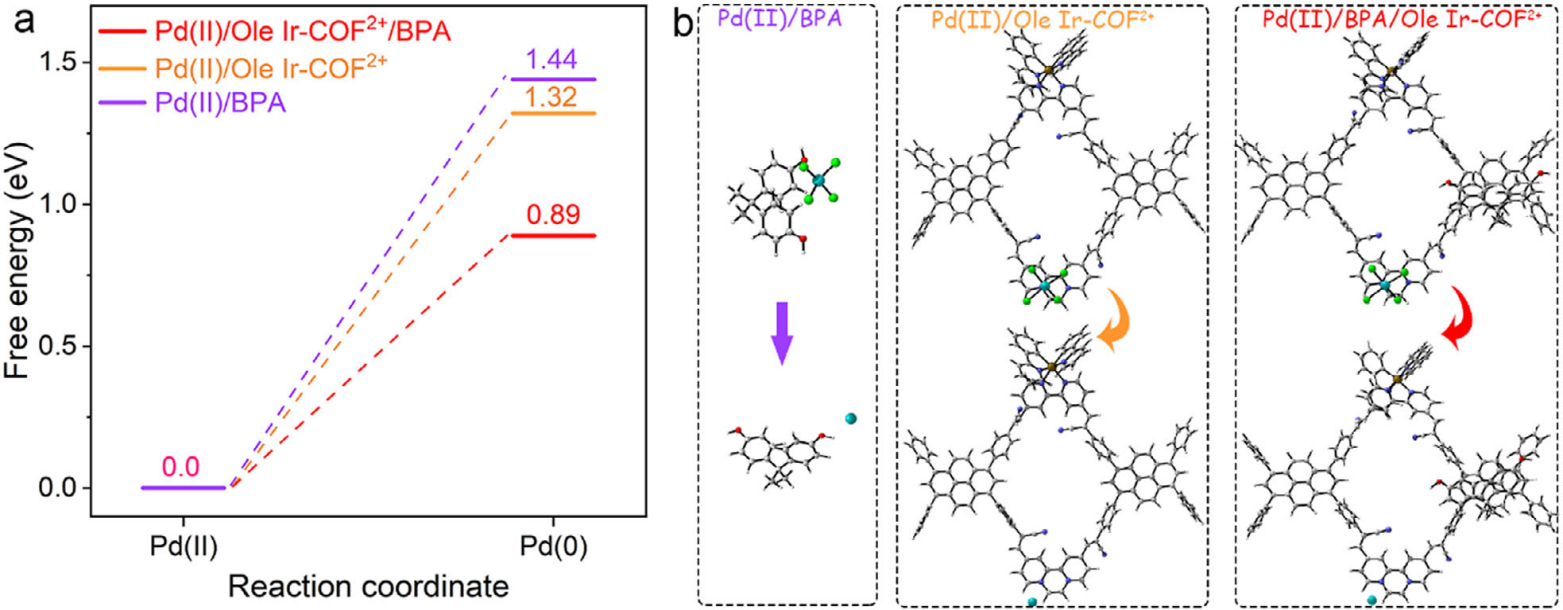
Theoretical evidence for reaction promotion via hole scavenging and barrier modulation. (a) Pd(II)/BPA/Ole Ir‐COF^2+^; Pd(II)/Ole Ir‐COF^2+^; and Pd(II)/BPA. (b) Optimization model of the reaction process.

The reaction energy barrier is 1.44 eV in the Pd(II)/BPA system, which decreases to 1.32 eV in Ole Ir‐COF^2+^ and 0.89 eV in the full ternary system, representing a 38% reduction, highlighting profound kinetic synergy (Figure 9a,b). Under illumination, the catalyst enables simultaneous thermodynamics for both half reactions, while BPA acts as a hole scavenger and a kinetic promoter via interfacial interactions. Our findings establish that reactant–catalyst interfacial synergy concurrently optimizes charge separation, thermodynamic driving force, and reaction kinetics, providing a new strategic paradigm for overcoming efficiency bottlenecks in advanced photocatalytic systems.

### 2.6 High‐performance Pd Recycling From Real Leachate

**FIGURE 10:**
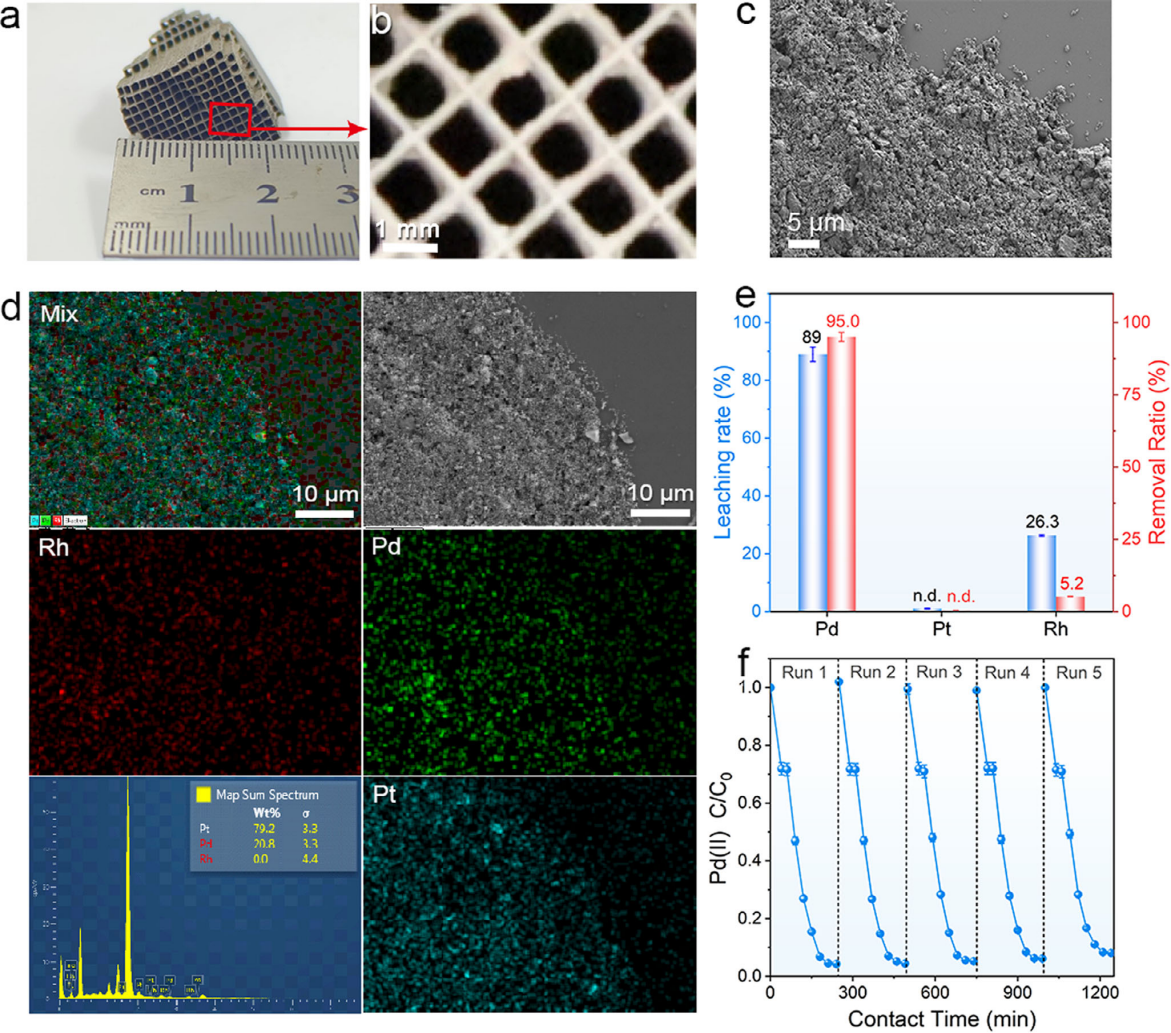
Selective and recyclable Pd recovery from real waste leachate. A diagram of actual samples of TWCs. (a) Diagram of actual samples of TWCs. (b) Magnified view of TWCs sample. (c) SEM image of ball milling TWCs coupled with (d) EDS mapping. (e) PGM ion leaching rate of hydrometallurgical (left axis) and its removal rate by Ole Ir‐COF^2+^ (right axis). (f) The reusability evaluation of the Ole Ir‐COF^2+^ for Pd(II) reduction over five cycles under light irradiation.

The complex architecture of spent three‐way catalytic converters (TWCs) presents a major challenge for platinum (Pt) group metal (PGM) recovery (Figure 10a–c) [61]. Their honeycomb washcoat confines trace PGMs (<0.2 wt.%) within a stabilizer–binder matrix, severely limiting metal accessibility. Mechanical ball milling disrupts this structure, exposing active sites and enabling EDS analysis, which shows Pt–Pd colocalization at the micrometer scale, in contrast to the weak Rh signal resulting from its ultralow abundance and encapsulation (Figure 10d). Aqua regia leaching produces an acidic leachate (pH 2), where Pd(II) concentration reaches 1546 ppm, vastly exceeding the Rh(III) (43 ppm) concentration, corresponding to leaching efficiencies of 89% and 26.3%, respectively. While Pd dissolves efficiently, Rh loss is attributed to volatile RhO_2_ formation during TWC operation. Pt resistance originates from a passivating oxide layer, as confirmed via EDS results (Figure S55) [62, 63].

The performance of Ole Ir‐COF^2+^ was evaluated in an authentic, multicomponent leachate. It exhibits exceptional specificity for Pd(II), achieving 95% capture with negligible uptake of Rh(III) or Pt(II) (Figure 10e), which corresponds to a selectivity factor of >345. Furthermore, the material demonstrates outstanding structural stability, retaining ∼92% of its initial Pd recovery capacity over five consecutive cycles (Figure 10f). Furthermore, the results of dark control experiments using actual leachate confirmed that surface adsorption or precipitation plays a minor role in the actual leachate system, while photocatalysis serves as the dominant mechanism driving Pd recovery (Figure S56). This combination of high selectivity and robust recyclability establishes Ole Ir‐COF^2+^ as a practical and efficient platform for targeted palladium recovery from complex industrial wastes.

## 3 Conclusion

This study demonstrates that designing a D–A–A cascade structure with spatially separated redox centers is a universal strategy to achieve efficient synergistic catalysis of competitive redox reactions. Based on this principle, we successfully synthesized a 1D fully conjugated Ole Ir‐COF^2+^. This material integrates broad‐spectrum absorption, directional charge migration, and highly accessible active sites via the synergistic integration of dual photosensitizing units and the edge cation interface. The strong built‐in electric field increases τ to 5.06 ns, threefold that of Ole COF^4+^, and reduces the E_b_ to 25.3 meV, considerably outperforming all reference COFs. Without sacrificial agents, Ole Ir‐COF^2+^ exhibits a remarkable Pd(II) extraction capacity of 1749 mg g^−1^, which is twofold and 15‐fold that of Ole COF^4+^ and g‐C_3_N_4_, respectively. The degradation rate constant of BPA is 0.1710 min^−1^, 13‐fold that of g‐C_3_N_4_. Theoretical studies show that BPA acts as a hole scavenger and pathway modifier, lowering the Pd(II) reduction barrier from 1.44 to 0.89 eV, substantially improving reaction kinetics. In actual leachate, the material maintains a Pd(II) capture rate of 95%, and the recovery rate stabilizes at 92% after five cycles, demonstrating excellent selectivity and stability. This study provides a new design paradigm for developing next‐generation integrated photocatalytic platforms for resource recovery and environmental remediation.

## Conflicts of Interest

The authors declare no conflicts of interest.

## Supporting information




**Supporting File**: advs75468‐sup‐0001‐SuppMat.docx.

## Data Availability

All data are either provided in the Article and its Supplementary Information or are available from the corresponding author upon request.
