# Supplementary material for: Spatially Separated Redox Centers in One‐Dimensional Sp2‐Carbon Covalent Organic Frameworks Enable Synergistic Photocatalytic Palladium Recovery and Bisphenol A Mineralization
Source: Adv Sci (Weinh). 2026 May 1;13(42):e75468. doi: 10.1002/advs.75468 (PMC13335508; doi:10.1002/advs.75468)
Supplement: Supplementary file 1 — Supporting File: advs75468‐sup‐0001‐SuppMat.docx. [file ADVS-13-e75468-s001.docx]

**Supplementary Information**

**Spatially separated redox centers in one-dimensional sp²-carbon covalent organic frameworks enable synergistic photocatalytic palladium recovery and** **bisphenol A mineralization**

Yi-Ru Chen^1,3^, Jing-Yi Li^1^, Yao Xiao^1^, Dan Zhong^1^, Lu Zhang^1^, Yu-Ting Xie^1^, Xiu Wang^1,3^, Yibao Li^1,^*, Wei-Rong Cui^1,^*, Jian-Ding Qiu^2^

^1^College of Chemistry and Materials, Gannan Normal University, Ganzhou, 341000, China

^2^School of Chemistry and Chemical Engineering, Nanchang University, Nanchang 330031, China

^3^School of Metallurgical Engineering, JiangXi University of Science and Technology, Ganzhou, 341000, China.

*E-mail addresses:* wrcui@gnnu.edu.cn (W.-R. Cui), liyibao@gnnu.edu.cn (Y. Li).

**1. Chemicals**

**Materials** 2,2',2'',2'''-(Pyrene-1,3,6,8-tetrayltetrakis(benzene-4,1-diyl))tetraacetonitrile (PyTT-CN), 4,4’,4’’,4’’’-(pyrene-1,3,6,8-tetrayl)tetraaniline (PyTTA), and 2,2′-bipyridine-4,4′-dicarboxaldehyde (BpyA) were purchased from Jilin Yanshen Technology Co., Ltd. Bisphenol A (BPA), acetaminophen (APAP), naproxen (NPX), diclofenac (DCF), rhodamine B (RhB), nitrotetrazolium blue chloride (NBT), 1,2-dichlorobenzene (o-DCB), n-butanol (n-BuOH), 1,4-dioxane, acetic acid (HOAc), p-benzoquinone (p-BQ), l-histidine, potassium hexacyanoferrate(III) (K_3_Fe(CN)_6_), potassium hexacyanoferrate(II) trihydrate (K_4_Fe(CN)_6_ 3H_2_O), nafion perfluorinated resin, 2,2,6,6-tetramethylpiperidine (TEMP), and 5,5-dimethyl-1-pyrrolidine N-oxide (DMPO) were purchased from Shanghai Titan Technology Co., Ltd. Isopropanol, tetrahydrofuran (THF), acetone, acetonitrile, 1,2-dibromoethane, NaBrO_3_, Na_2_SO_4_, NaOH, KOH, HCl, KCl, and KI were purchased from Sinopharm Chemical Reagent Co., Ltd. Na_2_PdCl_4_ was purchased from Anhui Zesheng Technology Co., Ltd. All chemical reagents were analytically pure and used directly without further purification. Wahaha drinking water was used in all experiments. All the purchased reagents were of analytical grade and used without further purification.

1. **Preparation Methods**

**Synthesis of model compound**


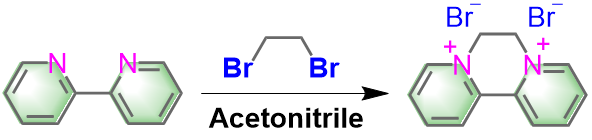


**Scheme S1. Synthesis route of model compound**.

2,2'-Bipyridine (1.56 g, 10 mmol) was dissolved in acetonitrile. 1.4 equivalents of 1,2-dibromoethane were added to the solution. The reaction solution was refluxed at 100 °C for 8 h. After the reflux, the precipitate was collected by filtration, washed with acetone, and dried in vacuum to obtain a yellow solid. Yield: 81%. ^1^H NMR (500 MHz, D_2_O) δ 9.25 (d, *J* = 5.9 Hz, 2H), 9.03 (d, *J* = 8.1 Hz, 2H), 8.95 (t, *J* = 8.0 Hz, 2H), 8.42 (t, *J* = 6.9 Hz, 2H), 5.39 (s, 4H).

**Synthesis of [(ppy)_2_-Ir-μ-Cl]_2_**

310.4 mg (2 mmol, 285.8 μL) ppy, 272 mg (0.9 mmol) IrCl_3_•3H_2_O, 6 mL ethoxyethanol, and 2 mL water were added to a 50 mL Schlenk tube. After three cycles of freeze-pump-thaw degassing, the mixture was heated at 120 °C under nitrogen for 24 h, during which time the formation of a yellow precipitate was observed. After cooling to ambient temperature, the precipitate was collected by vacuum filtration. The filter cake was thoroughly washed with deionized water (50 mL) and hexane (25 mL) to obtain the pure product [(ppy)_2_-Ir-μ-Cl]_2_ as a fine yellow powder (yield: 82%). This product was used for the next step without further purification. ^1^H NMR (400 MHz, CDCl_3_) δ 9.24 (d, *J* = 4.9 Hz, 1H), 7.87 (d, *J* = 7.9 Hz, 1H), 7.76 – 7.69 (m, 1H), 7.48 (dd, *J* = 7.8, 1.1 Hz, 1H), 6.80 – 6.71 (m, 2H), 6.58 – 6.52 (m, 1H), 5.93 (d, *J* = 7.1 Hz, 1H).


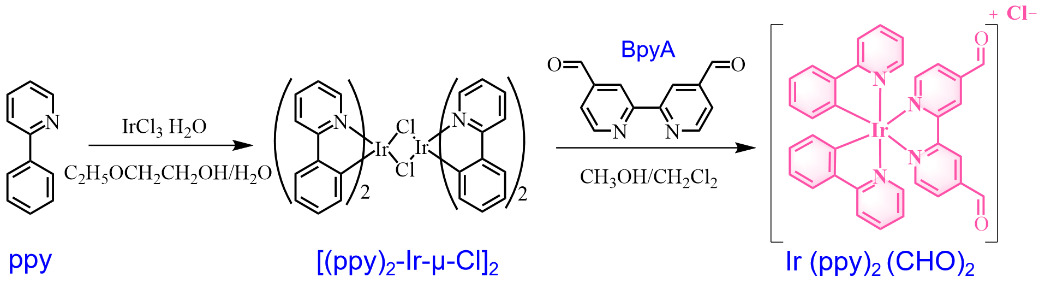


**Scheme S2**. **Synthesis route of [(ppy)_2_-Ir-μ-Cl]_2_.**

**Synthesis of Ir(ppy)_2_(CHO)_2_**

[(ppy)_2_-Ir-μ-Cl]_2_ (96.7 mg, 0.093 mmol), BpyA (47.36 mg, 0.2232 mmol), 10 mL of methanol, 30 mL of dichloromethane. Reflux at 80 °C for 8 h under N_2_ environment. Obtain red powder (a small amount of crystals) with a yield of (yield: 75 %). Purify by column chromatography (DCM:CH_3_OH= 10:1). The target compound was prepared according to the method reported in the literature^1,2,3,4^.


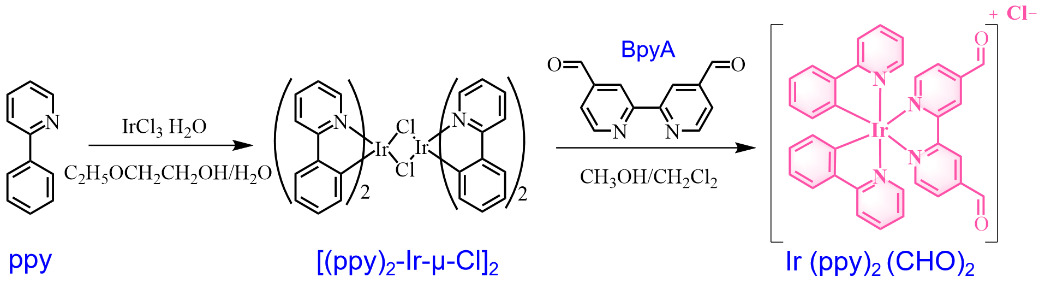


**Scheme S3**. **Synthesis route of Ir(ppy)_2_(CHO)_2_.**

**Synthesis of Ole Ir-COF**

Firstly, 2,2’-bipyridine-4,4’-dicarboxaldehyde and Ir(ppy)_2_(CHO)_2_ in a molar ratio of 7:3 were mixed with 2,2',2'',2'''-(pyrene-1,3,6,8-tetrayltetrakis(benzene-4,1-diyl))tetraacetonitrile (0.02 mmol, 13.3 mg) in the reaction solution of dioxane/4M KOH (10/1 by vol.; 1.1 mL) in a Pyrex tube (10 mL). Then it was degassed by three freeze-pump-thaw cycles. The reaction proceeded at 120 °C for 5 days. After which time, the product was collected by filtration, washed with THF using Soxhlet extraction for 24 h and dried at 40 °C under vacuum for 48 h to give the Ole Ir-COF.

**Synthesis of Ole Ir-COF^2+^**

Then, Ole Ir-COF (20 mg) was dispersed in a mixture of acetonitrile (9 mL) and dibromoalkane (1 mL) in a flask. Then, the mixture was refluxed at 82 °C under nitrogen atmosphere. After 8 h of reaction, the product was collected by filtration, washed with THF (3 × 10 mL), and dried under vacuum at 40°C for 48 h to obtain quaternized Ole Ir-COF^2+^. Yield: 85%.

**Synthesis of Ole COF^4+^**

Firstly, 2,2' - bipyridine-4,4' - formaldehyde is reacted with 1.4 equivalents of dibromoethane at 82 °C for 12 h to synthesize 2,2' - bipyridine-4,4' - formaldehyde dibromide. Then, the quaternized monomer of 2,2' - bipyridine-4,4' - formaldehyde (0.1 mmol, 40 mg) was mixed with 2,2′,2′′,2′′′-(pyrene 1,3,6,8-tetrakis (phenyl-4,1-diyl) tetraacetonitrile (0.05 mmol, 33.14 mg) in a reaction solution of o-DCB/n-BuOH/4M KOH (volume ratio 5/5/1; 1.1 mL). Then degas through three freeze-thaw cycles. The reaction was carried out at 120 °C for 3 days. Afterwards, the product was collected by filtration, washed with THF, and dried under vacuum at 40 °C for 48 h to obtain Ole COF^4+^ with yields of 67.5 %.

**Synthesis of Im Ir-COF^2+^**

Firstly, 2,2’-bipyridine-4,4’-dicarboxaldehyde and Ir(ppy)_2_(CHO)_2_ in a molar ratio of 7:3 were mixed with 4,4’,4’’,4’’’-(pyrene-1,3,6,8-tetrayl)tetraaniline (0.06 mmol, 34 mg) in the reaction solution of o-DCB/n-BuOH/6 M HOAc (6/4/2 by vol.; 1.2 mL). Then it was degassed by three freeze-pump-thaw cycles. The reaction proceeded at 120 °C for 5 days. After which time, the product was collected by filtration, washed with THF using Soxhlet extraction for 24 h and dried at 40 °C under vacuum for 48 h to give the Im Ir-COF. Yield: 83%. Then, Im Ir-COF (20 mg) was dispersed in a mixture of acetonitrile (9 mL) and dibromoalkane (1 mL) in a flask. Then, the mixture was refluxed at 82 °C under nitrogen atmosphere. After 8 h of reaction, the product was collected by filtration, washed with THF (3 × 10 mL), and dried under vacuum at 40 °C for 48 h to obtain quaternized Im Ir-COF^2+^.

**3. Characterization Methods**

**3.1 Characterizations**

Fourier-transform infrared (FT-IR) spectra were recorded on a Nicolet iS50 FT-IR spectrometer. Solid-state ^13^C cross-polarization magic-angle spinning (^13^C CP/MAS NMR) spectra were recorded with a 4-mm double-resonance MAS probe; a sample spinning rate of 8.0 kHz, a contact time of 2 ms (ramp 100), and a pulse delay of 3 s were applied. X-ray photoelectron spectroscopy (XPS) spectra of the samples and photocatalytic reduction products of PdCl_4_^2-^ were performed on ground powders using a Thermo VG Multilab 2000X. The test Passing-Energy full spectrum is 50 eV, the narrow spectrum is 20 eV, the step length is 0.05 eV, the residence time is 40-50 ms, and the charge correction is carried out with C 1s = 284.80 eV binding energy as the energy standard. The morphology of the material was imaged by a scanning electron microscope (SEM, JEM-2010, JEOL). The morphology of the prepared nanoparticles was observed using a transmission electron microscope (TEM) at an accelerating voltage of 200 kV (JEOL JEM-F200, Japan). The samples were outgassed at 120 °C for 12 h before the measurements. The nitrogen adsorption and desorption isotherms were measured at 77 K using a Micromeritics ASAP 2020M system. Surface areas were calculated from the adsorption data using Brunauer-Emmett-Teller (BET) methods. The pore-size-distribution curves were obtained via the non-local density functional theory (NLDFT) method. Metal ions concentrations were determined using an inductively coupled plasma optical emission spectrometer (ICP-OES, Ultima expert, France). The thermal properties of the COFs were evaluated using a STA PT1600 Linseis thermogravimetric analysis (TGA) instrument over the temperature range of 30 to 800 °C under nitrogen atmosphere with a heating rate of 10 °C/min. Solid UV–vis diffuse reflectance spectra (DRS) were recorded with a PE Lambda 900 UV/vis spectrophotometer at room temperature. The UV-Vis diffuse reflectance spectra of liquid samples were recorded at room temperature using a single-beam UV-Vis spectrophotometer (N5000, China). The transformation products of Bisphenol A (BPA) were quantified by LCMS (Agilent 6545Q-TOF LC/MS, China). The concentrations of BPA in the supernatant were determined by means of a LC-2030 high-performance liquid chromatography (LC) (Shimadzu, Japan).

**3.2 Powder X-ray diffraction (PXRD)**

Powder X-ray diffraction (PXRD) data of the monomers and obtained samples were collected on a Bruker AXS D8 Advance A25 Powder X-ray diffractometer (40 kV, 40 mA) using Cu Kα (λ=1.5406 Å) radiation.

The layer distance of COFs was estimated based on the Bragg’s law:

$$\begin{aligned} d=\frac{n\lambda}{2\sin\theta}\#\left( 1 \right) \end{aligned}$$

where λ is the wavelength of the X-ray and *θ* is the incident angle.

**3.3 Photoluminescence (PL) spectroscopy**

The steady-state PL spectra and time-resolved PL decay curves were recorded on Edinburgh FLS1000 spectrometer. Time-resolved PL decays were fitted by a biexponential model:

$$\begin{aligned} I_{t}=I_{0}+A_{1}e^{\frac{-t}{\tau1}}+A_{2}e^{\frac{-t}{\tau2}}\#\left( 2 \right) \end{aligned}$$

$$\begin{aligned} \tau_{ave}=\frac{A_{1}\tau_{1}^{2}+A_{2}\tau_{2}^{2}}{A_{1}\tau_{1}+A_{2}\tau_{2}}\#\left( 3 \right) \end{aligned}$$

where I_(t)_ is the intensity of TRPL signal, I₀ is the maximum fluorescence intensity at the beginning of the fluorescence decay process A_1_ and A_2_ are the amplitudes, τ_1_ and τ_2_ are the lifetimes, τ_ave_ is the intensity average lifetime.

The temperature-dependent PL spectra were also recorded on Edinburgh FLS1000

spectrometer under the temperature ranging from 80 to 280 K to reveal the binding energy of excitons in COFs. The exciton binding energy E_b_ is calculated via fitting the intensity data with the following equation:

$$\begin{aligned} \frac{I_{t}}{I_{0}}=\frac{1}{1+Ae^{\frac{-E_{b}}{\left( K_{B}T \right)}}}\#\left( 4 \right) \end{aligned}$$

where I_t_ represents the normalized integrated PL intensity and I_0_ is the value at 0 K. E_b_ is the binding energy. A is a proportional constant and k_B_ is the Boltzmann constant.

**3.4** **Electron paramagnetic resonance (EPR) characterization**

Spin trapping EPR measurements were recorded using a Bruker A300 spectrometer with DMPO as spin-trapping agent for the detection of superoxide radical. TEMP was served as the spin trapping agent for the detection of singlet oxygen (^1^O_2_).

Hydroxyl radicals (•OH): Prepare a sample aqueous solution with a concentration of 1 mg/ml. After ultrasonic dispersion, take 100 μl of the solution and add 100 μl of 100 mM DMPO solution. Mix well and seal the tube with a capillary tube. Use a 300 W xenon lamp as the light source to collect data for 5 min in the dark and light.

Superoxide free radicals (^•^O_2_^−^): Prepare a sample methanol solution with a concentration of 1 mg/ml. After ultrasonic dispersion, take 100 μl of the solution and add 100 μl of 100 mM DMPO solution. Mix well and seal the capillary tube. Use a 300 W xenon lamp as the light source to collect data for 5 min in the dark and light.

Singlet oxygen (^1^O_2_): Prepare a sample aqueous solution with a concentration of 1 mg/ml, after ultrasonic dispersion, take 100 μl of the solution and add 100 μl of 100 mM TEMP solution, mix well, seal the capillary tube, put it into the sample tube. Use a 300 W xenon lamp as the light source to collect data for 5 min in the dark and light.

**3.5** **Photoelectrochemical measurements**

Photoelectrochemical properties were measured on an electrochemical workstation (CHI 760E, Chenhua Instruments) by a standard three-electrode system, in which the Ag/AgCl electrode (saturated KCl solution) was used as a reference electrode and Pt sheet as the counter electrode. The working electrode was prepared by the drop-casting method. In a typical process, the as synthesized COF powder (5 mg) was dispersed in a mixture of ethanol (500 µL) and Nafion solution (20 µL) and sonicated for 30 min. 200 µL of the prepared slurry was dropped onto a indium-tin oxide (ITO) glass (1×1 cm^2^) and the COF-based photoelectrode was obtained by drying ethanol in ambient conditions. Photoelectrochemical measurements were performed under irradiation of a 300W Xenon lamp (λ>420 nm) with the power density of 300 Mw cm^-2^ in Na_2_SO_4_ solution (0.1 M, pH=7). The potential conversion relation is obtained according to the Nernst equation:

$$\begin{aligned} E_{SHE}=E_{\left( {Ag}/{AgCl} \right)}+0.059\times pH+0.197V\#\left( 5 \right) \end{aligned}$$

Electrochemical impedance spectra (EIS): Mix solution of KCl, K_3_Fe(CN)_6_ and K_4_Fe(CN)_6_·3H_2_O (pH 5; 0.1 mol L^−1^) was used as the electrolyte in the EIS measurements. The EIS frequency ranged from 10^2^ to 10^6^ Hz with an AC amplitude of 10 mV at the open circuit voltage under room-light illumination.

Transient photocurrent responses (TPR): The light on/off photocurrent response was recorded with time interval of 30 s.

Mott-Schottky (M-S): M-S plots were investigated at 1000 Hz, 2000 Hz and 3000 Hz respectively, with 5 mV amplitude.

**4. Simulation and calculations**

Geometry optimisations were performed using Gaussian 16, employing the B3LYP functional and 6-31G(d) basis set. The geometry optimisations were run with dispersion corrections from Grimme’s D3 model with Becke-Johnson damping factors. Harmonic vibrational frequencies calculations were performed to confirm the stationary points as true minima. Single point energy calculation level: M06-2X/6-311G(d,p). SDD pseudopotential and pseudopotential basis sets are used for metallic elements.

For the analysis of IGMH interaction, molecular frontier orbital, molecular surface electrostatic potential, excited state electron and hole analysis are plotted into thermal maps, all of which are extracted by Multiwfn wave function analysis software^5^ and analyzed and plotted by VMD software^6^. TD-DFT method and CAM-B3LYP method are used to calculate the excited states of molecules. The adsorption energy is calculated by subtracting the energy of adsorbent and the energy of adsorbate from the energy of adsorption complex. The ball and stick structures were created using GaussView 6.1.18.

**5.** **Evaluation of photocatalytic performance**

Batch photo-reduction experiments were conducted in a jacketed quartz beaker photoreactor. Cooling water was circulated around the reactor to maintain the system temperature of (25 ± 0.2 °C). A 300 W Xe lamp ( λ ≥ 400 nm, light intensity 1 kW m^-2^) was employed as the light source.

**5.1 Pd uptake experiment**

Pd mother solution (1,000 ppm) was prepared by dissolving Na_2_PdCl_4_ in a deionized water. Lower concentration solutions were prepared by diluting this solution.

***PdCl_4_^2-^ sorption isotherms.*** To obtain the PdCl_4_^2-^ adsorption isotherms for various adsorbents, 5 mg of sorbent materials were added into 25 mL aqueous solutions with different concentrations of PdCl_4_^2-^ in the range of 50 to 1000 ppm, respectively. After stirring for 60 min to reach adsorption-desorption equilibrium, the suspension was irradiated with a 300 W Xe lamp immediately. The treated solutions were filtrated through a 0.22-μm membrane filter. The supernatant was analyzed using ICP-OES analysis to determine the remaining PdCl_4_^2-^ concentration. The adsorbed amount at equilibrium (mg g^-1^) was calculated by:

$$\begin{aligned} q_{e}=\frac{\left( C_{0}-C_{e} \right)\times V}{m}\#\left( 6 \right) \end{aligned}$$

where V is the volume of the treated solution (mL) and m is the amount of adsorbent used (g), and C_0_ and C_e_ are the initial concentration and the final equilibrium concentration of PdCl_4_^2^, respectively.

***PdCl_4_^2-^ removal kinetics.*** Pd^2+^ aqueous solution (50 mL, ~100 ppm) with the addition of adsorbents (10 mg) were added to an Erlenmeyer flask with stirring. At appropriate time intervals, aliquots (0.5 mL) were taken from the mixture, and the adsorbents were separated by syringe filter. The PdCl_4_^2-^ concentrations in the resulting solutions were analyzed by ICP-OES. The removal efficiency and adsorption capacity of PdCl_4_^2-^ were calculated as follows:

$$\begin{aligned} R_{e}=\frac{\left( C_{0}-C_{e} \right)}{C_{0}}\times100\%\#\left( 7 \right) \end{aligned}$$

$$\begin{aligned} q_{t}=\frac{\left( C_{0}-C_{t} \right)\times V}{m}\#\left( 8 \right) \end{aligned}$$

where V is the volume of the treated solution (mL) and m is the amount of used adsorbent (mg), and C_0_ and C_t_ are the initial concentration and the concentration of PdCl_4_^2-^ at t (min), respectively.

***K_d_ value calculation.*** Pd adsorption kinetics were studied by adding 5 mg of Ole Ir-COF^2+^ into 100 ppm Pd solution (200 mL). Several data points were obtained between 0 and 30 min by taking 2 mL aliquots from the solution (every 5 min). The solutions were filtered using a 0.22 μm pore sized membrane filter and the filtrate was analyzed with ICP-OES to determine the residual metal concentration. The distribution coefficient (K_d_) value as used for the determination of the affinity and selectivity of sorbents for PdCl_4_^2-^, is given by the equation:

$$\begin{aligned} K_{d}=\frac{\left( C_{0}-C_{e} \right)}{C_{e}}\times\frac{V}{m}\#\left( 9 \right) \end{aligned}$$

where V is the volume of the treated solution (mL), m is the amount of adsorbent (g), C_0_ is the initial concentration of PdCl_4_^2-^, and C_e_ is the equilibrium concentration of PdCl_4_^2-^.

***SF value calculation.*** The selectivity factor (SF) is a key parameter for evaluating the selectivity of an adsorbent for a specific target adsorbate (A) relative to a competing adsorbate (B).

$$\begin{aligned} {SF}_{\frac{A}{B}}=\frac{K_{d,A}}{K_{d,B}}\#\left( 10 \right) \end{aligned}$$

Where K_d,A_ and K_d,B_ are the distribution coefficients (mL g^-1^), which represent the affinity of the adsorbent for the target substance at adsorption equilibrium.

***PdCl_4_^2-^ adsorption at different pH values.*** The pH of the solution was varied in the range of 3-7 using HCl or NaOH and monitored by a pH meter. Then, 5 mg of COF was added into the solution and stirred for 12 h until equilibrium was reached. The solution obtained at each pH value was filtered using a 0.22 μm pore sized membrane filter, and the filtrate was analyzed with ICP-OES to determine the residual metal concentration.

***Selectivity tests for palladium.*** In order to test selective ***PdCl_4_^2-^*** capture from wastewater, an aqueous solution (200 mL) containing 10 ppm PdCl_4_^2-^, Ni^2+^, Zn^2+^, Pb^2+^, Fe^2+^, Ca^2+^, Co^2+^ and Cd^2+^ was prepared, and then 5 mg of Ole Ir-COF^2+^ was added to this solution and stirred overnight. The solutions were filtered using a 0.22 μm pore sized membrane filter, and the filtrate was analyzed with ICP-OES to determine the residual metal concentration.

***Recyclability test:*** Pd-contacted COFs were placed in 0.2 M thiourea (50 mL) and 0.01 M HCl solution (50 mL), 0.1 M NaOH solution (50 mL), and water (50 mL) in sequence overnight. The adsorbents were filtered, washed with water, and dried under vacuum. The solutions filtered through a 0.22 μm membrane filter. The filtrate was analyzed via ICP-OES to determine the residual PdCl_4_^2^***^-^*** concentrations.

**5.2 Photocatalytic degradation experiments**

To further evaluate the photodegradation performance of the prepared catalyst in aqueous solution, BPA was used as a model organic pollutant. In brief, a 300 W xenon lamp (λ≥400 nm) was used as visible light. In a typical photodegradation system, 10 mg of the prepared catalyst was dispersed in 50 mL of BPA solution (50 mg L^-1^). Before illumination, the above suspension was continuously stirred in the dark for 30 min to achieve the absorption-desorption equilibrium between the catalyst and BPA molecules. After that, 0.5 mL of the reaction suspension was taken at the specified time intervals, and the BPA concentration was determined by liquid LC at the strongest absorption peak at 280 nm. The reproducibility of each set of reactions was ensured by three repeated experiments.

To verify the universality of Ole-Ir-COF^2+^ for the degradation of other emerging pollutants, we studied four emerging pollutants, including acetaminophen (APAP), diclofenac (DCF), naproxen (NPX) and rhodamine B (RhB). Similar to bisphenol A, the initial concentration of these four emerging pollutants was set at 50 mg/L. The details of the LC determination of these emerging pollutants are shown in Supplementary Table 5.

In order to survey the ROS generated in photocatalytic reaction, the same photocatalytic experiments with addition of radical scavengers were carried out except for the control reaction conditions. Isopropanol (IPA, 10% v/v), p-benzoquinone (p-BQ, 1 mM), L-histidine (1 mM), NaBrO_3_ (1 mM), and KI (1 mM) were used to capture •OH, •O_2_^−^, ^1^O_2_, e^−^, and h^+^ radicals, respectively. In addition, nitroblue tetrazolium (NBT) was selected as •O_2_^−^probe to quantify the •O_2_^−^generation on •O_2_^−^ultraviolet-visible spectrophotometer with strongest characteristic peak at 259 nm.

**6.** **Leaching experiments**

**6.1 The leaching process in this work is as follows**

Step 1: Waste ternary catalysts (TWCs) were crushed in a ball mill and then sieved through a 200-mesh sieve to obtain powder with a particle size less than 75 µm. The TWCs were heated in a muffle furnace at 800 °C with a heating rate of 5 °C per minute for 8 h to remove organic impurities and oxidize the metals.

Step 2: Impurities were removed by leaching in 6 M HCl at room temperature for 2 h.

Step 3: The solid-liquid ratio was 1:10 (g/mL) of calcined powder to aqua regia, and the mixture was stirred at a constant temperature for 12 h.

*Note 1*: In catalysts used at high temperatures, PGMs may be sintered and encapsulated, resulting in a lower direct aqua regia leaching rate. Due to the high standard potentials of platinum group metals (PGMs), they are almost insoluble in hydrochloric acid, and oxidants such as chlorine, hydrogen peroxide, and nitric acid are usually added to promote their leaching.

Step 4: Solid-liquid separation. Vacuum filtration was used to remove residues (ceramic fragments, undissolved impurities), the filtrate was collected and the pH was adjusted to 1-2 (to avoid metal hydrolysis). The leachate was filtered through a 0.22 µm filter membrane and then diluted with water to 50 ml. The concentrations of Pd, Pt, and Rh ions were tested using ICP-OES.

**6.2. Digestion Procedure**

*Note 2:* The platinum supported on TWCs is platinum dioxide, which is stable in air but insoluble in concentrated acids and aqua regia. Therefore, dissolving the platinum on the catalytic converter using concentrated nitric acid or aqua regia is not feasible; microwave digestion must be used to completely dissolve the entire catalytic converter to leach the platinum.

Step 1: Microwave Calcination Pretreatment. The waste automotive catalyst is crushed and ball-milled to obtain powder with a main particle size less than 75 µm. The catalyst powder is then uniformly mixed with a recycled reagent (NaClO_3_ and NaHSO_4_·H_2_O, mass ratio 1:1) in a ceramic crucible at a specific ratio. The crucible containing the mixture is wrapped with insulating material and placed in a microwave reactor. The reaction is carried out at the set calcination temperature (450-550 °C) and holding time (40-80 min). The microwave power is controlled at 300-330 W. After the reaction, the sintered block is removed and cooled.

Step 2: Water Leaching. The cooled sintered block is dispersed in water. During microwave roasting, the precious metals are converted into water-soluble compounds. Through solid-liquid separation, the target metals (Pd, Rh, Pt) are released into the aqueous solution.

The concentrations of Pt, Pd, and Rh in the leachate were also quantified by ICP-OES, and the leaching rate (Li) of each metal was calculated by Eq. (11).

$$\begin{aligned} L_{i}(\%)=\frac{Quanity of mental i in leachate}{Quanity of metal i in solid sample}\#(11) \end{aligned}$$

**Table S1 .** Detailed EIS fitting parameters of COFs.

|  | **X=0%** | | **X=10%** | | **X=30%** | | **X=50%** | | **X=70%** | |
| --- | --- | --- | --- | --- | --- | --- | --- | --- | --- | --- |
| **Element** | **Value** | **Error(%)** | **Value** | **Error(%)** | **Value** | **Error(%)** | **Value** | **Error(%)** | **Value** | **Error(%)** |
| **R_s_** | 21.12 | 1.503 | 11.32 | 1.023 | 17.56 | 1.606 | 27.28 | 1.429 | 18.45 | 3.666 |
| **R_ct_** | 390.9 | 1.531 | 71.56 | 5.040 | 57.82 | 7.724 | 75.5 | 1.097 | 148 | 8.776 |
| **CPE1-T** | 1.35E-6 | 4.737 | 2.54E-5 | 5.094 | 1.48E-5 | 12.538 | 2.10E-6 | 9.497 | 1.84E-6 | 18.544 |
| **CPE1-P** | 0.841 | 0.536 | 0.619 | 0.711 | 0.692 | 1.663 | 0.828 | 1.110 | 0.846 | 2.022 |

**Table S2.** Experimental and simulated porosity of COFs

| **COFs** | **Experimental pore**  **diameter (nm)** | **Experimental BET**  **surface area (m^2^ g^-1^)** | **Experimental pore volume (cm³ g⁻¹)** |
| --- | --- | --- | --- |
| **Ole Ir-COF^2+^** | 1.29 | 92.6 | 0.175 |
| **Ole Ir-COF** | 1.30 | 532 | 0.419 |
| **Ole COF^4+^** | 1.25 | 200 | 0.273 |
| **Im Ir-COF^2+^** | 1.30 | 612 | 0.461 |

***Note 3***: The determined BET specific areas are ranged from 92.6 to 612 m^2^ g^-1^ for 1D COFs.

**Table S3.** XPS of N for fresh, after absorbed, after irradiation.

| **XPS of N1s** | | **Fresh** | | | **After absorbed** | | | **After irradiation** | | |
| --- | --- | --- | --- | --- | --- | --- | --- | --- | --- | --- |
| **Ole Ir-COF** | Binding energy (ev) | 398.40 | 399.50 | / | 398.70 | 399.80 | / | 398.60 | 399.80 | / |
|  | Area (%) | 55.3 | 44.7 |  | 20.4 | 79.6 |  | 26.1 | 73.9 |  |
|  | FWHM (eV) | 1.16 | 1.18 |  | 1.30 | 1.41 |  | 1.36 | 1.38 |  |
| **Ole Ir-COF^2+^** | Binding energy (ev) | 398.40 | 399.50 | 402 | 398.84 | 399.96 | 402.00 | 398.60 | 399.80 | 402.00 |
|  | Area (%) | 18.4 | 66.8 | 14.8 | 10.9 | 70.1 | 19.0 | 34.5 | 51.3 | 14.2 |
|  | FWHM (eV) | 1.07 | 1.54 | 1.21 | 1.35 | 1.27 | 1.85 | 2.18 | 1.40 | 1.83 |
| **Ole COF^4+^** | Binding energy (ev) | 398.45 | 399.35 | 401.85 | / | | | 398.60 | 399.80 | 401.85 |
|  | Area (%) | 29.6 | 53.3 | 17.1 |  |  |  | 29.3 | 56.0 | 14.7 |
|  | FWHM (eV) | 1.10 | 1.43 | 1.39 |  |  |  | 1.68 | 1.44 | 1.51 |
| **Im Ir-COF** | Binding energy (ev) | 398.30 | 399.40 | / | / | | | / | | |
|  | Area (%) | 57.4 | 42.6 |  |  |  |  |  |  |  |
|  | FWHM (eV) | 1.18 | 1.41 |  |  |  |  |  |  |  |
| **Im Ir-COF^2+^** | Binding energy (ev) | 398.30 | 399.45 | 401.50 | / | | | 398.75 | 399.83 | 402 |
|  | Area (%) | 46.6 | 43.0 | 10.4 |  |  |  | 23.5 | 67.0 | 9.5 |
|  | FWHM (eV) | 1.15 | 1.50 | 2.33 |  |  |  | 1.37 | 1.42 | 1.33 |

**Table S4 .** Quantitative EDS analysis of COFs.

| **Elements** | | **C** | **N** | **Ir** | **Br** |
| --- | --- | --- | --- | --- | --- |
| **Ole Ir-COF^2+^** | nominal values (%) | 88.07 | 9.17 | 0.92 | 1.80 |
|  | atomic ratios (%) | 88.48 | 8.99 | 1.07 | 1.46 |
| **Ole Ir-COF** | nominal values (%) | 89.50 | 9.50 | 0.95 | / |
|  | atomic ratios (%) | 89.75 | 9.55 | 0.70 |  |
| **Ole COF^4+^** | nominal values (%) | 86.36 | 9.10 | / | 4.50 |
|  | atomic ratios (%) | 86.92 | 8.75 |  | 4.33 |
| **Im Ir-COF^2+^** | nominal values (%) | 87.25 | 9.80 | 0.98 | 2.00 |
|  | atomic ratios (%) | 87.23 | 9.60 | 0.94 | 2.23 |

**Table S5 .** Detailed EIS fitting parameters of four COFs.

|  | **Ole Ir-COF^2+^** | | **Ole Ir-COF** | | **Ole COF^4+^** | | **Im Ir-COF^2+^** | |
| --- | --- | --- | --- | --- | --- | --- | --- | --- |
| **Element** | **Value** | **Error(%)** | **Value** | **Error(%)** | **Value** | **Error(%)** | **Value** | **Error(%)** |
| **R_s_** | 17.56 | 1.606 | 30.41 | 1.477 | 21.12 | 1.503 | 14.94 | 1.760 |
| **R_ct_** | 57.82 | 7.724 | 239.6 | 4.365 | 390.9 | 1.531 | 95.47 | 7.717 |
| **CPE1-T** | 1.48E-5 | 12.538 | 1.761E-6 | 7.072 | 1.35E-6 | 4.737 | 1.32E-5 | 7.615 |
| **CPE1-P** | 0.692 | 1.663 | 0.786 | 0.821 | 0.841 | 0.536 | 0.727 | 1.044 |

**Table S6.** TR-PL decay lifetimes parameter of COFs.

| **Sample** | **A1** | **τ_1_** | **A2** | **τ_2_** | **τ_ave_** |
| --- | --- | --- | --- | --- | --- |
| **Ole Ir-COF^2+^** | 3.07 | 5.755 | 3.43 | 0.852 | 5.06 |
| **Ole Ir-COF** | 75.41 | 1.71 | 1.68 | 0.365 | 1.70 |
| **Ole COF^4+^** | 0.313 | 3.9 | 6.649 | 0.5 | 1.41 |
| **Im Ir-COF^2+^** | 1.012 | 5.066 | 1.776 | 0.651 | 4.25 |

**Table S7.** Comparison of palladium uptake amount by Ole Ir-COF^2+^, Ole Ir-COF, Ole COF^4+^ and Im Ir-COF^2+^ and other reported for a wide range of acidity.

| **Number** | **Photocatalyst** | **Conditions** | **Equilibration time (min)** | **pH** | ***q_e_* (mg g^-1^)** | **Ref.** |
| --- | --- | --- | --- | --- | --- | --- |
| **1** | **Tfpa-Od** | 2-500 ppm | 10 | 7 | 372.59 | 7 |
| **2** | **POP-Py** | 25-800 ppm | 10 | 7 | 708 | 8 |
| **3** | **POP-pNH_2_-Py** | 25-800 ppm | 10 | 7 | 743 | 8 |
| **4** | **POP-oNH_2_-Py** | 25-800 ppm | 10 | 7 | 752 | 8 |
| **5** | **CITCF-500** | 800 ppm | 30 | 6 | 929 | 9 |
| **6** | **CTF-S** | 80-1000 ppm | 120 | 3 M HNO_3_ | 212.9 | 10 |
| **7** | **CTF-L** | 80-1000 ppm | 120 | 3 M HNO_3_ | 324.5 | 10 |
| **8** | **UiO-66-NH_2_** | 100 ppm | 10 | 1 | 167 | 11 |
| **9** | **UiO-66-Pyta** | 100 ppm | 5 | 4.5 | 294.1 | 12 |
| **10** | **IPOF-Cl** | 100 ppm | 60 | 7 | 754 | 13 |
| **11** | **Tp-DG_Cl_** | 5-150 ppm | 180 | 2 | 342.1 | 14 |
| **12** | **VP-AMPS-CS5** | 100 ppm | 360 | 3 | 184.9 | 15 |
| **13** | **Tp-Azo-COF/SiO_2_** | 16 mmol L^-1^ | 120 | 3 M HNO_3_ | 85.4 | 16 |
| **14** | **CDCR** | 50-800 ppm | 150 | 3 | 204.3 | 17 |
| **15** | **TFBPy/CS aerogel** | 10-250 ppm | 120 | 1 | 274.4 | 18 |
| **16** | **TFBBPY-OMe-COF** | 20-600 ppm | 30 | 7 | 532 | 19 |
| **17** | **Ole Ir-COF^2+^** | 50-1000 ppm | 120 | 5 | 1749 | **This work** |
|  | **Ole Ir-COF** | 50-1000 ppm | 120 | 5 | 1275 |  |
|  | **Ole COF^4+^** | 50-1000 ppm | 120 | 5 | 894 |  |
|  | **Im Ir-COF^2+^** | 50-1000 ppm | 120 | 5 | 1390 |  |

**Table S8.** XPS of Pd for after absorbed, after irradiation.

| **XPS of Pd 3d** | | **After absorbed** | | **After irradiation** | |
| --- | --- | --- | --- | --- | --- |
| **Ole Ir-COF** | Binding energy (ev) | 337.80 | 343.10 | 337.46 | 342.80 |
|  | Area (%) | 55.6 | 44.4 | 11.8 | 88.2 |
|  | FWHM (eV) | 1.96 | 2.08 | 1.55 | 1.82 |
| **Ole Ir-COF^2+^** | Binding energy (ev) | 338.00 | 343.30 | 337.45 | 342.80 |
|  | Area (%) | 54.6 | 45.4 | 56.9 | 43.1 |
|  | FWHM (eV) | 1.88 | 2.08 | 1.69 | 1.82 |
| **Ole COF^4+^** | Binding energy (ev) | / | | 337.51 | 342.85 |
|  | Area (%) |  |  | 56.1 | 43.9 |
|  | FWHM (eV) |  |  | 1.45 | 1.61 |
| **Im Ir-COF^2+^** | Binding energy (ev) | / | | 337.53 | 342.86 |
|  | Area (%) |  |  | 56 | 44 |
|  | FWHM (eV) |  |  | 1.57 | 1.77 |

**Table S9.** X-ray Diffraction card of crystallized palladium. (Pd: PDF # 88-2335).

| **2-Theta** | **d (Å)** | **Intensity** | **(h k l)** |
| --- | --- | --- | --- |
| 40.009 | 2.2517 | 100 | (1 1 1) |
| 46.534 | 1.9500 | 43.9 | (2 0 0) |
| 67.923 | 1.3789 | 20.9 | (2 2 0) |
| 81.849 | 1.1759 | 20.9 | (3 1 1) |
| 86.344 | 1.1258 | 5.7 | (2 2 2) |

**Table S10** Molecular structures of organic compounds and LC settings**^20^.**

| Compound | Structure | Detection  Wavelength (nm) | Column  Temp  (℃) | Mobile  Phase (v/v) |
| --- | --- | --- | --- | --- |
| Paracetamol  (APAP) | 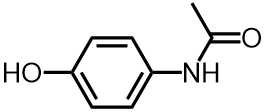 | 242 | 30 | Water:acetonitrile  = 85:15 |
| Diclofenac  (DCF) | 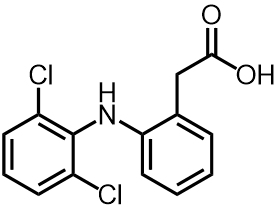 | 276 | 40 | Water (0.1% formic  acid) : acetonitrile  = 60:40 |
| Naproxen  (NPX) | 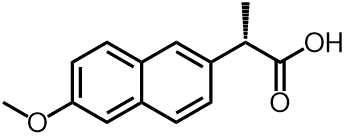 | 254 | 30 | Water (0.2% formic  acid) : methanol  = 35:65 |
| Bisphenol A  (BPA) |  | 280 | 40 | Water : methanol  = 30:70 |

***Note 4:*** The obtained filtrate was measured by UV-visible spectrophotometer at the characteristic absorption peak (RhB is 554 nm).


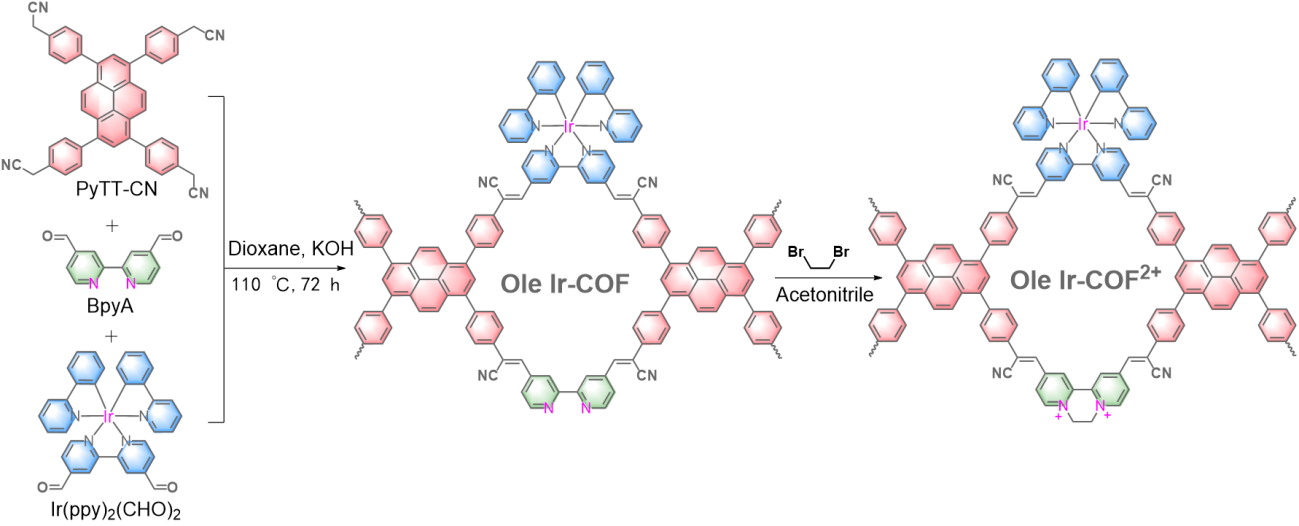
**Figure S1.** Synthesis of Ole Ir-COF and Ole Ir-COF^2+^.


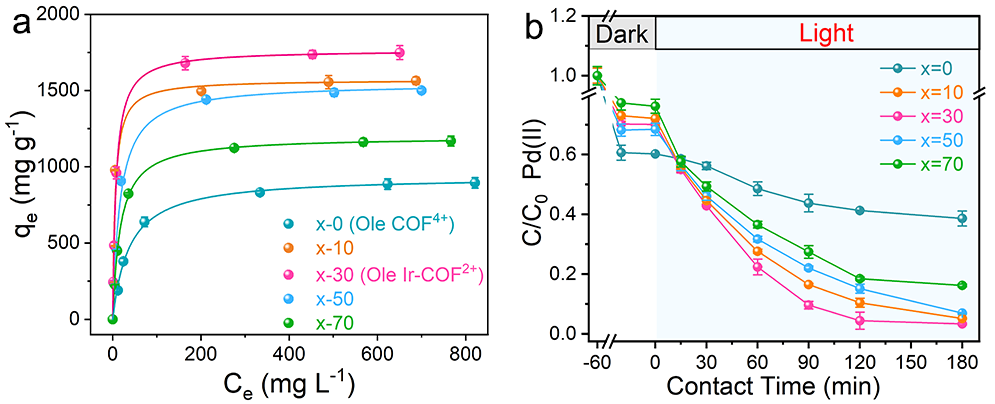


**Figure S2.** The adsorption isotherms of x-0, x-10, x-30, x-50 and x-70, Where x represents the molar ratio of Ir(ppy)_2_(CHO)_2_.

***Note*** 5: To determine the optimal loading amount of iridium complexes in COFs, a series of fully conjugated cationic 1D sp^2^C COFs (x = 0 (Ole COF^4+^), 10, 30 (Ole Ir-COF^2+^), 50, and 70, where x represents the molar ratio of Ir(ppy)_2_(CHO)_2_) were synthesized and their Pd(II) extraction abilities under light irradiation were compared. COFs with Ir(ppy)_2_(CHO)_2_ content below 30% exhibited limited light absorption capacity, resulting in constrained Pd(II) extraction under illumination.


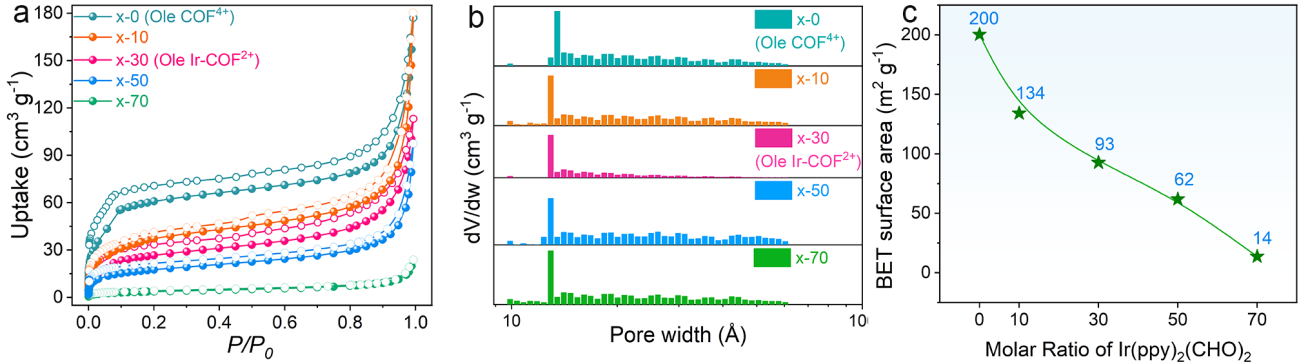


**Figure S3.** (a) Nitrogen sorption isotherms and (b) pore size distribution curves of x-0, x-10, x-30, x-50 and x-70, Where x represents the molar ratio of Ir(ppy)_2_(CHO)_2_. (c) BET surface areas for the Ir-coordinated COFs. When the Ir(ppy)_2_(CHO)_2_ content exceeded 30%, partial blockage of COF channels by functional groups likely occurred, leading to obscured active sites that could not be efficiently utilized. Increasing the iridium complex loading from 10 to 70 mmol% induced a linear decrease in the BET surface area of the fully conjugated COFs from 200 m^2^ g^-1^ to 14 m^2^ g^-1^, confirming the progressive pore-blocking effect of the functional groups.


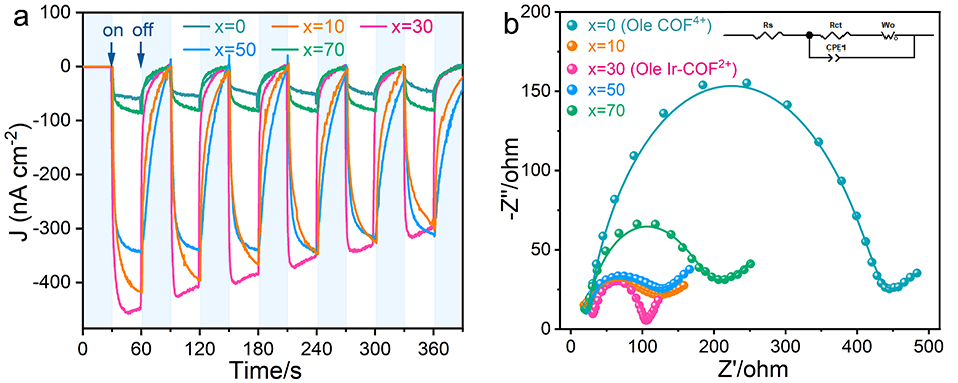


**Figure S4**. (a) EIS and (b) TPR of x-0, x-10, x-30, x-50 and x-70. The arc radius in the Nyquist plot (corresponding to charge transfer resistance) decreased initially and then increased as x varied from 0 to 70, reaching a minimum at x = 30%. This minimum signifies the highest electron-hole separation efficiency. The impedance value changes with frequency, and the point represents the original data and the curve represents the fitted curve. Consequently, Ole Ir-COF^2+^ with an Ir(ppy)_2_(CHO)_2_ content of 30 % was selected for subsequent experiments, as it provides the optimal balance between performance and cost-effectiveness. This result was corroborated by the experimental results of adsorption capacity and BET surface areas.


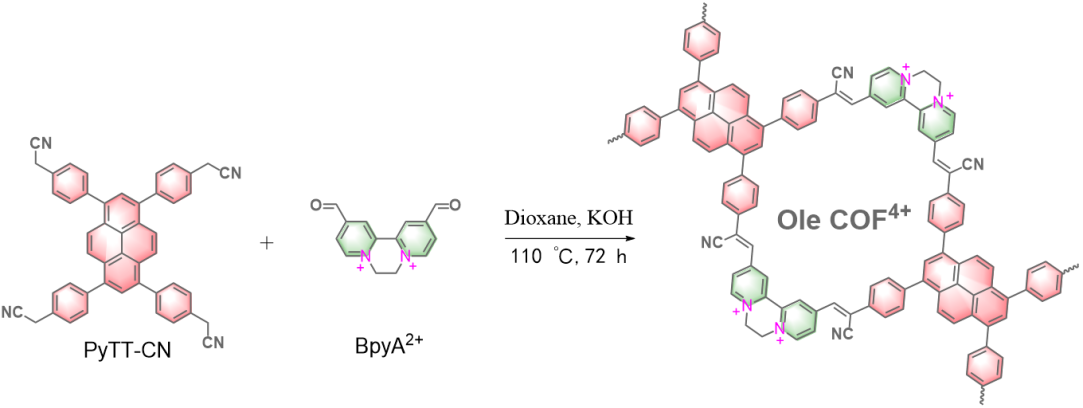


**Figure S5**. Synthesis of Ole COF^4+^ via the bottom-up method.


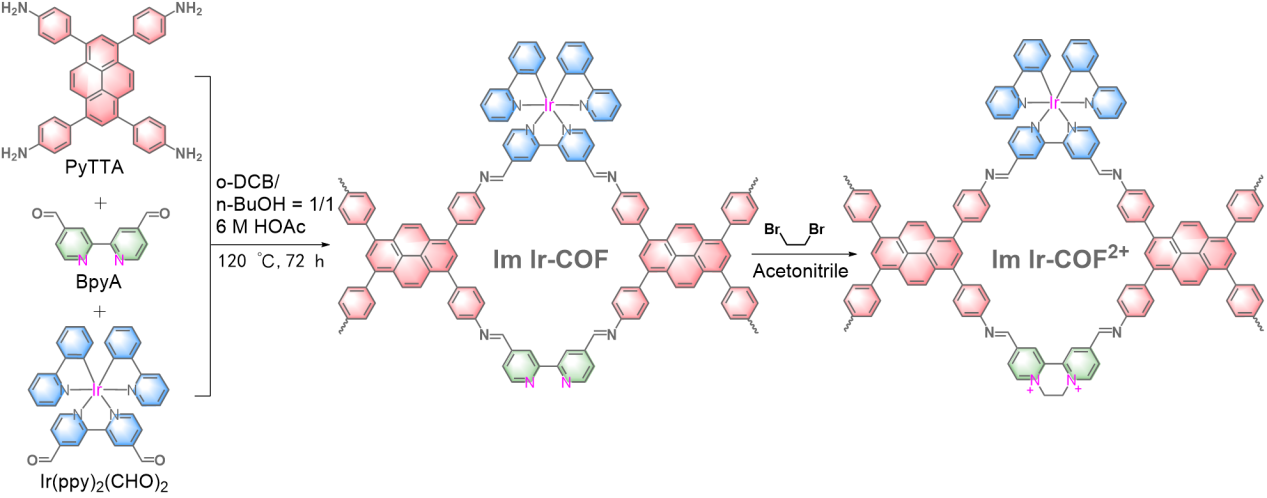


**Figure S6.** Synthesis of Im Ir-COF^2+^.


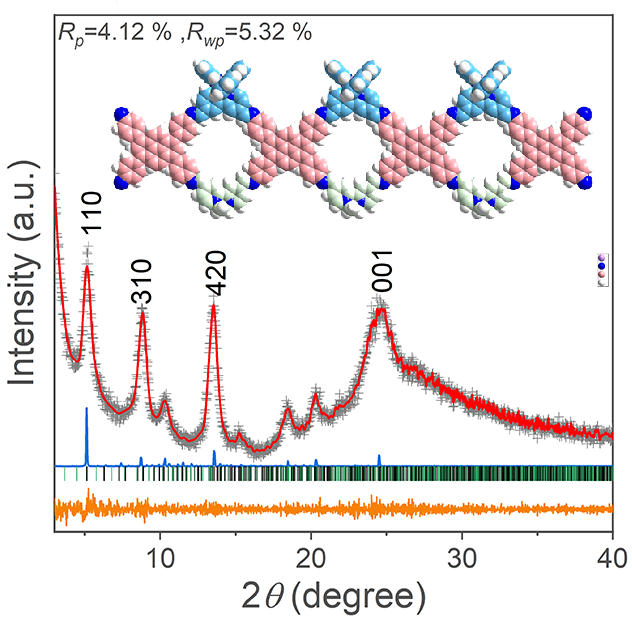


**Figure S7.** Crystal structure characterization of COFs. Experimental (black) and Pawley-refined (red) PXRD patterns with simulated AA-stacking structures for a Im Ir-COF^2+^.


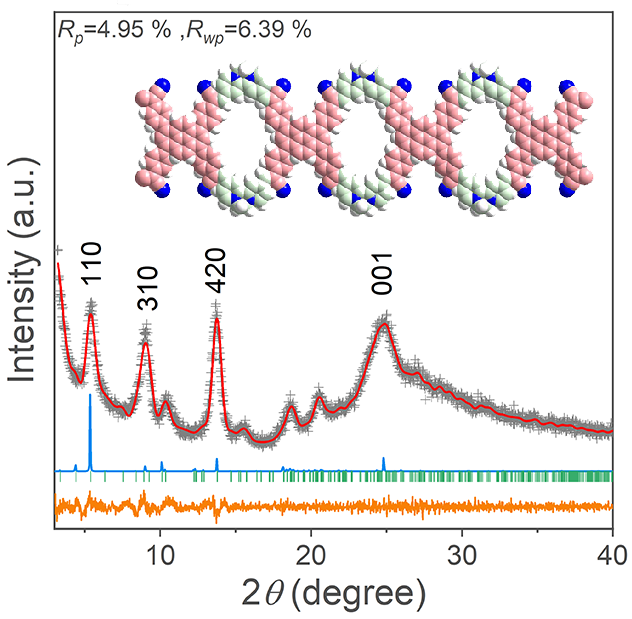


**Figure S8**. Crystal structure characterization of COFs. Experimental (black) and Pawley-refined (red) PXRD patterns with simulated AA-stacking structures for a Ole COF^4+^.


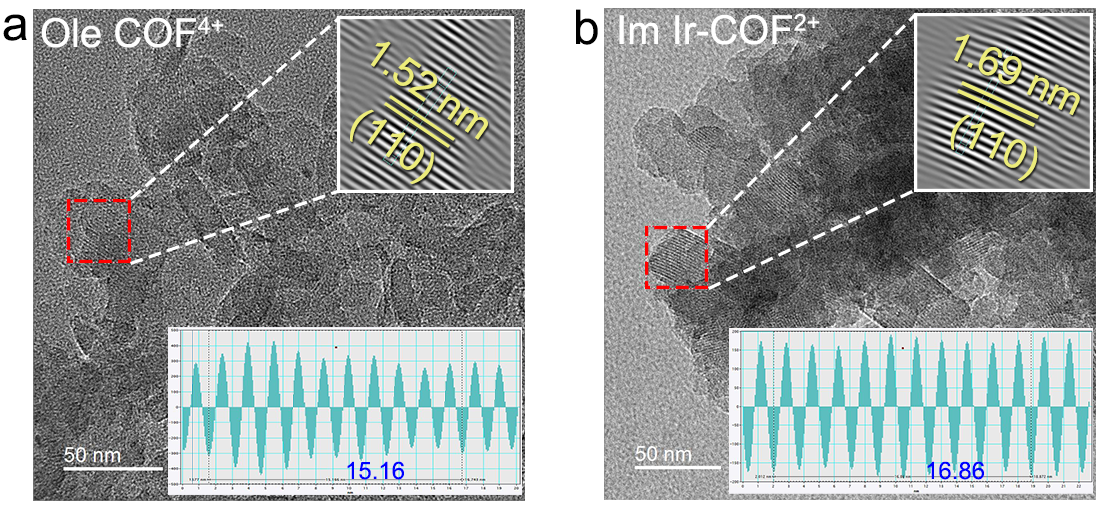


**Figure S9**. HR-TEM images of (a) Ole COF^4+^ and (b) Im Ir-COF^2+^.


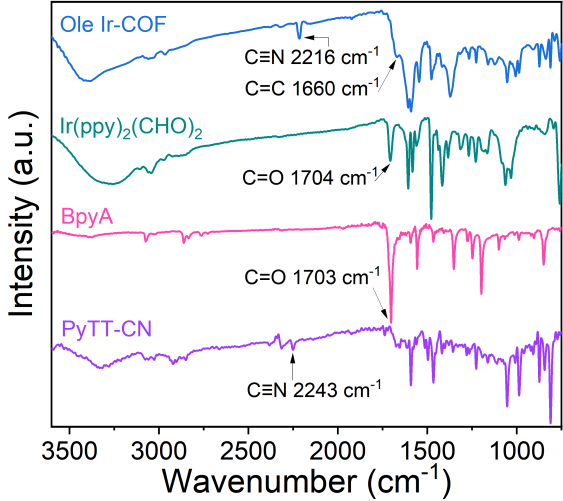


**Figure S10.** FT-IR spectra of BpyA, PyTT-CN, Ir(ppy)_2_(CHO)_2_ and Ole Ir-COF.


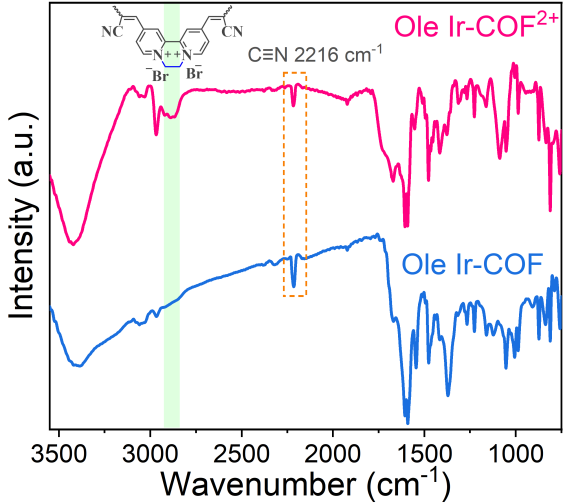


**Figure S11.** FT-IR spectra of Ole Ir-COF and Ole Ir-COF^2+^.


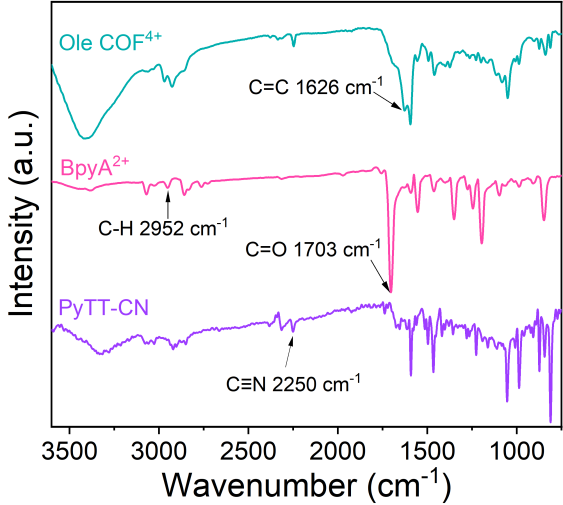


**Figure S12**. FT-IR spectra of BpyA^2+^, PyTT-CN and Ole COF^4+^.


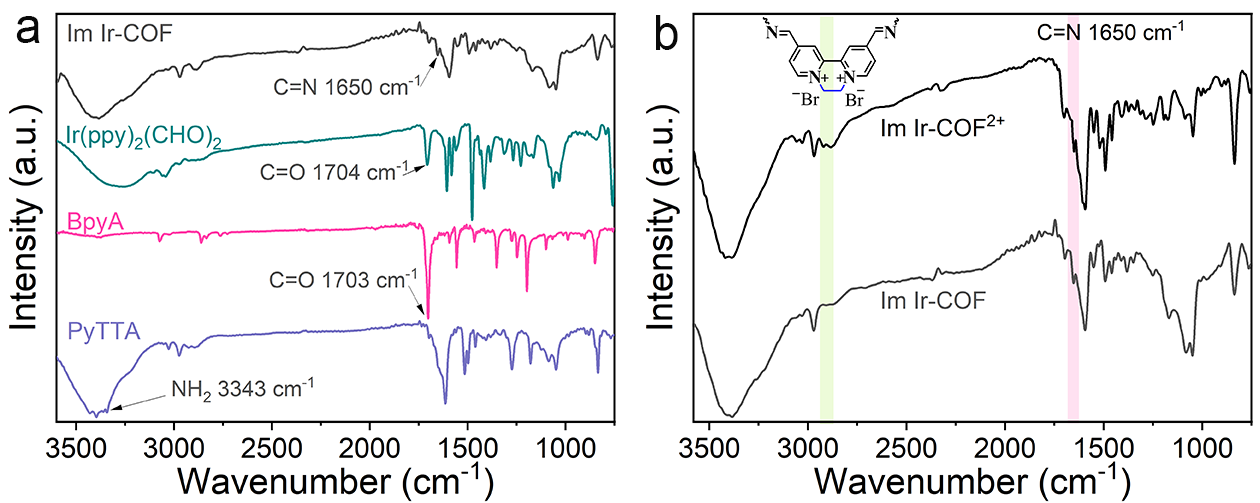


**Figure S13.** (a) FT-IR spectra of BpyA, PyTTA, Ir(ppy)_2_(CHO)_2_ and Im Ir-COF. (b) FT-IR spectra of Im Ir-COF and Im Ir-COF^2+^.


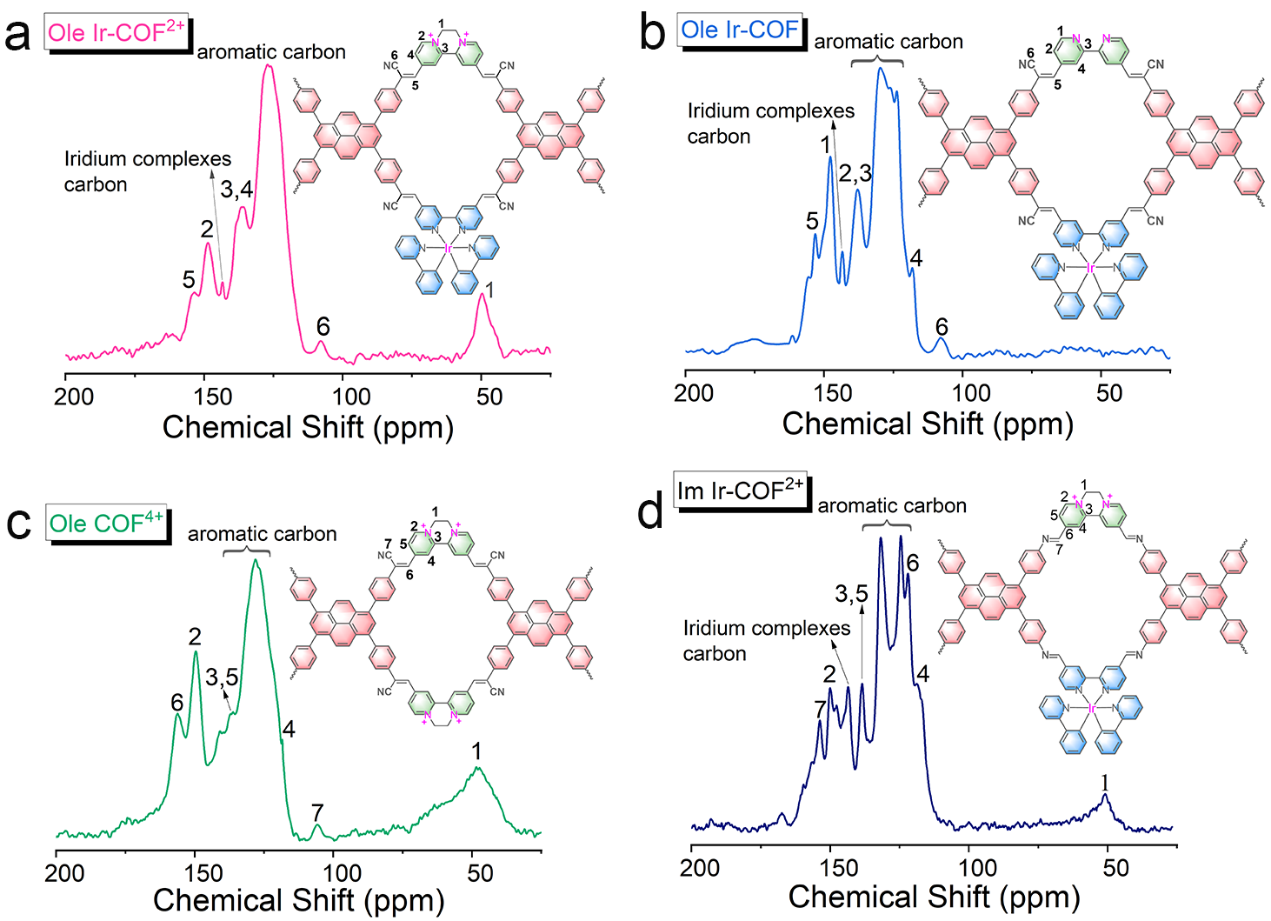


**Figure S14.** Solid-state ^13^C CP-MAS NMR spectra of (a) Ole Ir-COF^2+^ (b) Ole Ir-COF (c) Ole COF^4+^ and (d) Im Im-COF^2+^.


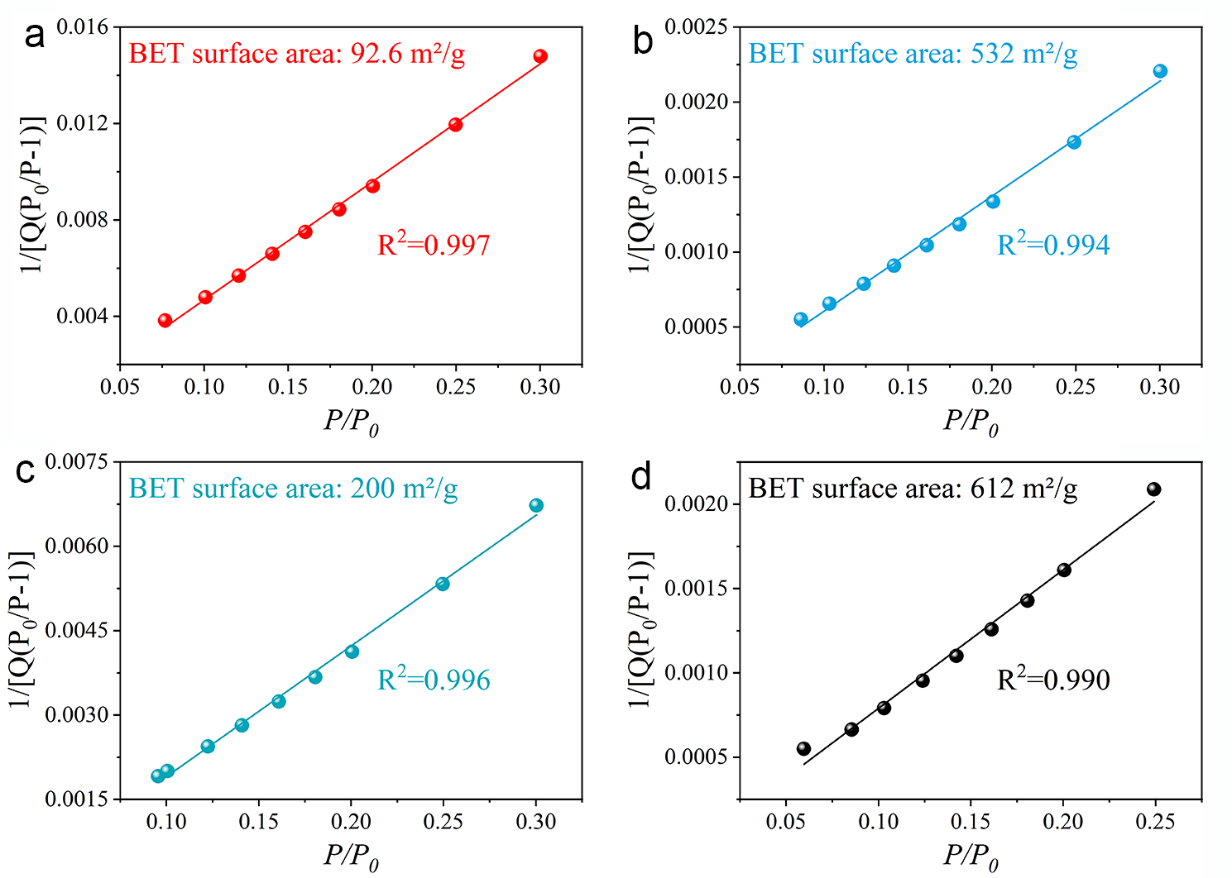


**Figure S15**. BET surface area plots of (a) Ole Ir-COF^2+^, (b) Ole Ir-COF, (c) Ole COF^4+^ and (d) Im Ir-COF^2+^ calculated from the isotherms.


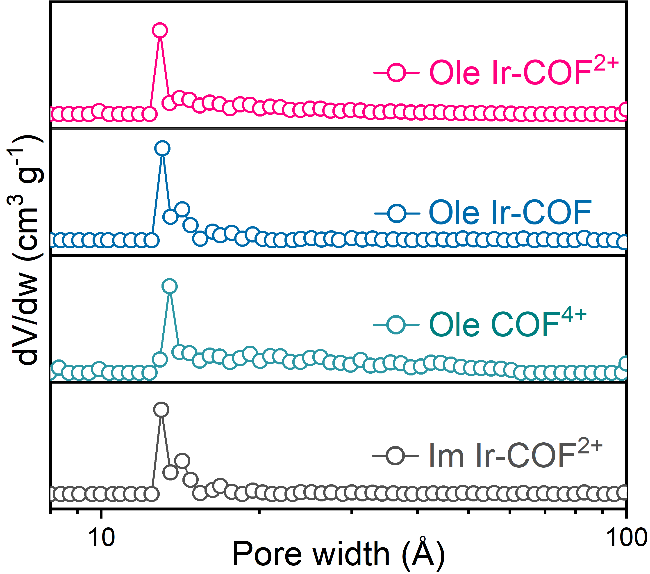


**Figure S16.** The pore size distributions of Ole Ir-COF^2+^, Ole Ir-COF, Ole COF^4+^, and Im Ir-COF^2+^ calculated from non-local density functional theory.


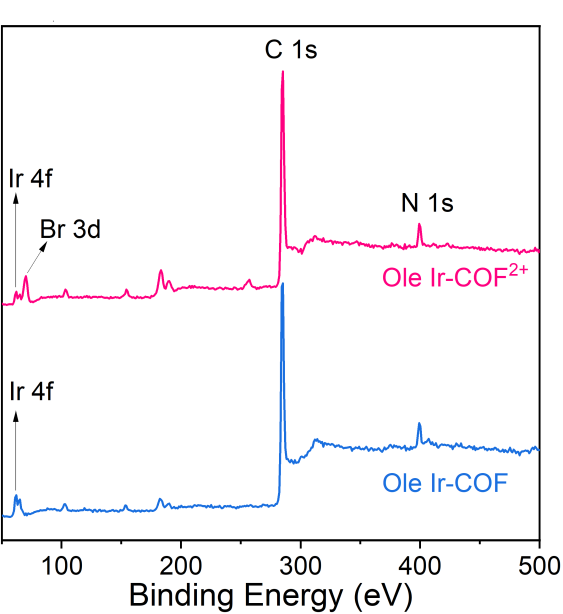


**Figure S17.** XPS survey spectra of Ole Ir-COF^2+^ and Ole Ir-COF.


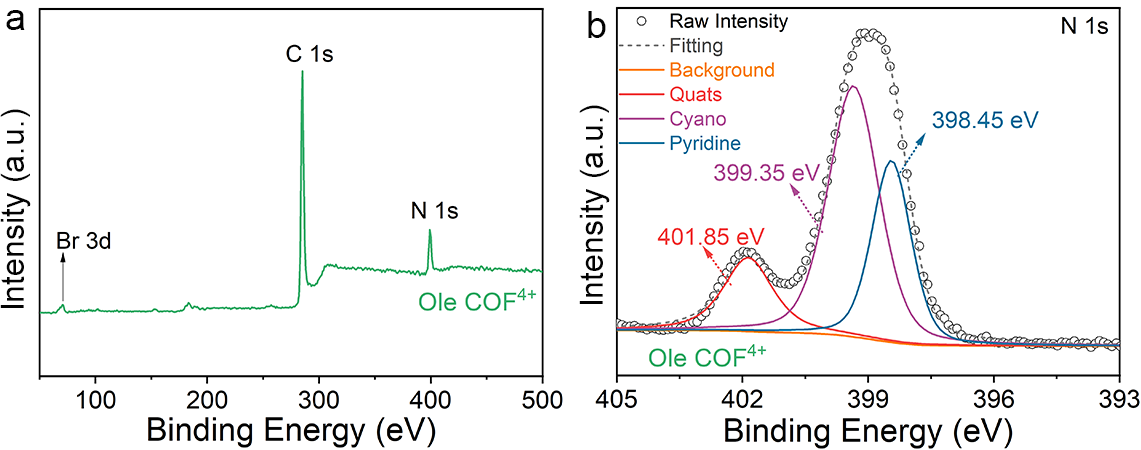


**Figure S18**. (a) XPS survey spectra of Ole COF^4+^. (b) The N 1s XPS spectra of Ole COF^4+^.


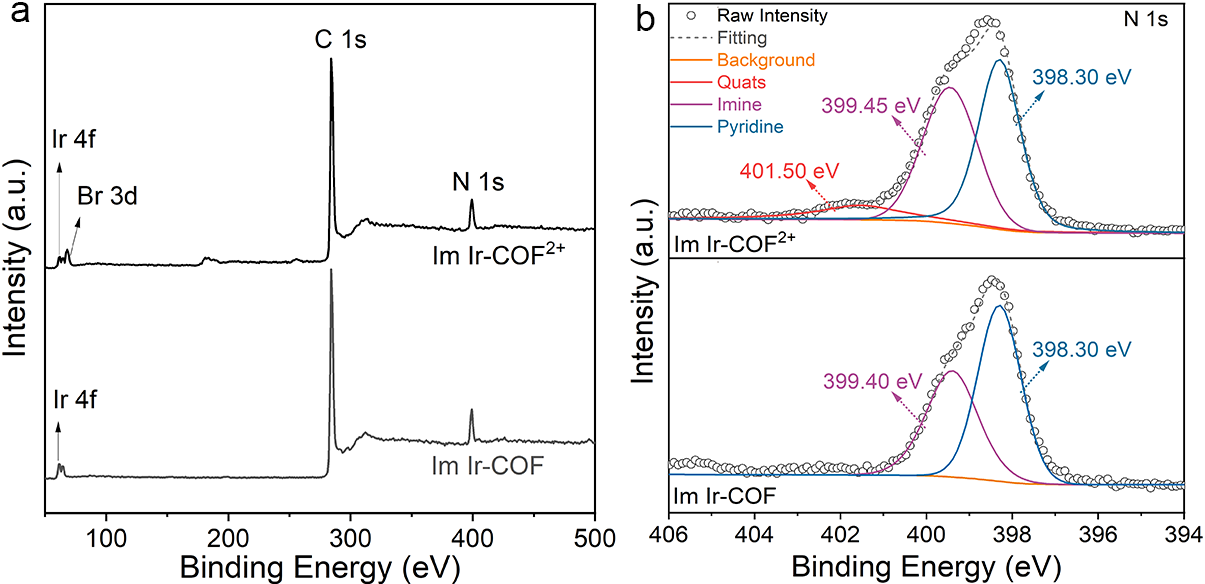


**Figure S19.** (a) XPS survey spectra of Im Ir-COF^2+^ and Im Ir-COF. (b) The N 1s XPS spectra of Im Ir-COF^2+^ and Im Ir-COF.


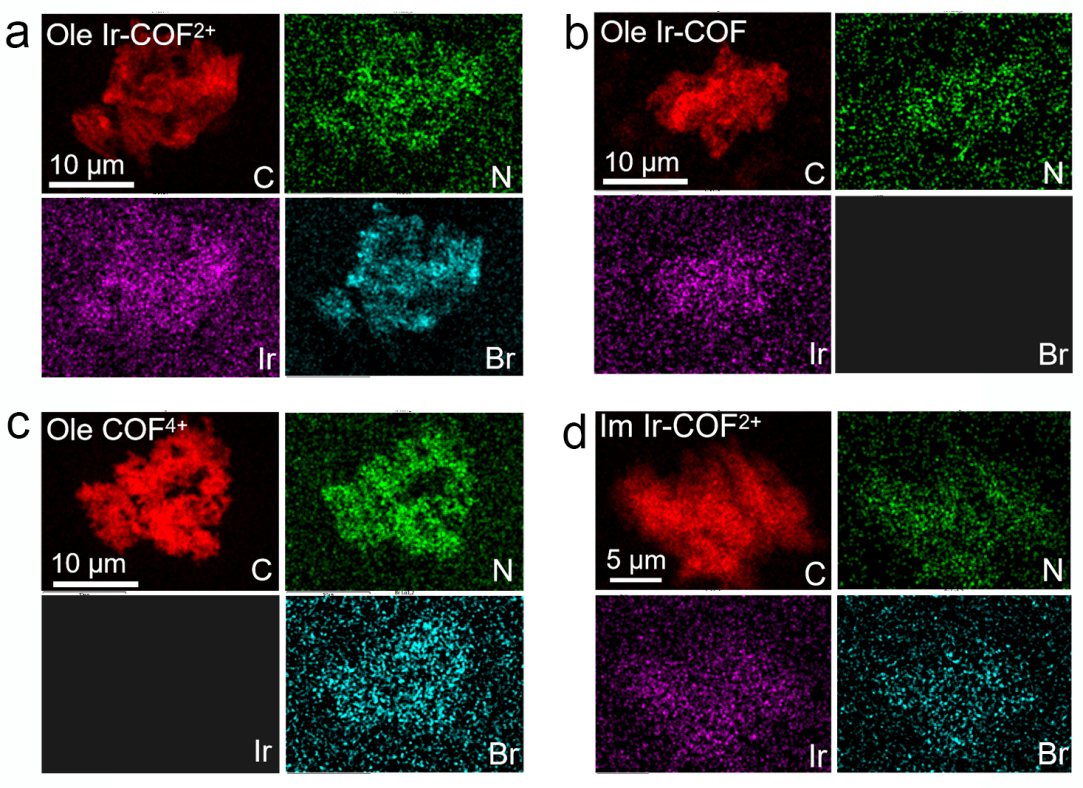


**Figure S20.** EDS element mapping images of (a) Ole Ir-COF^2+^, (b) Ole Ir-COF, (c) Ole COF^4+^ and (d) Im Ir-COF^2+^.


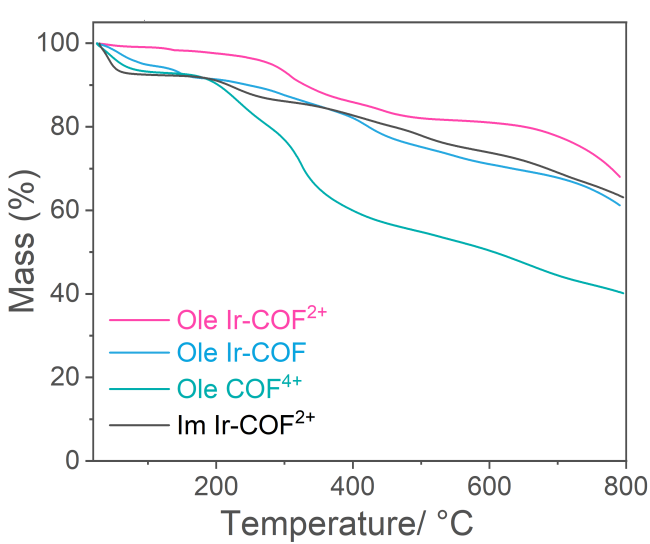


**Figure S21.** Thermogravimetric curves of Ole Ir-COF^2+^, Ole Ir-COF, Ole COF^4+^ and Im Ir-COF^2+^.


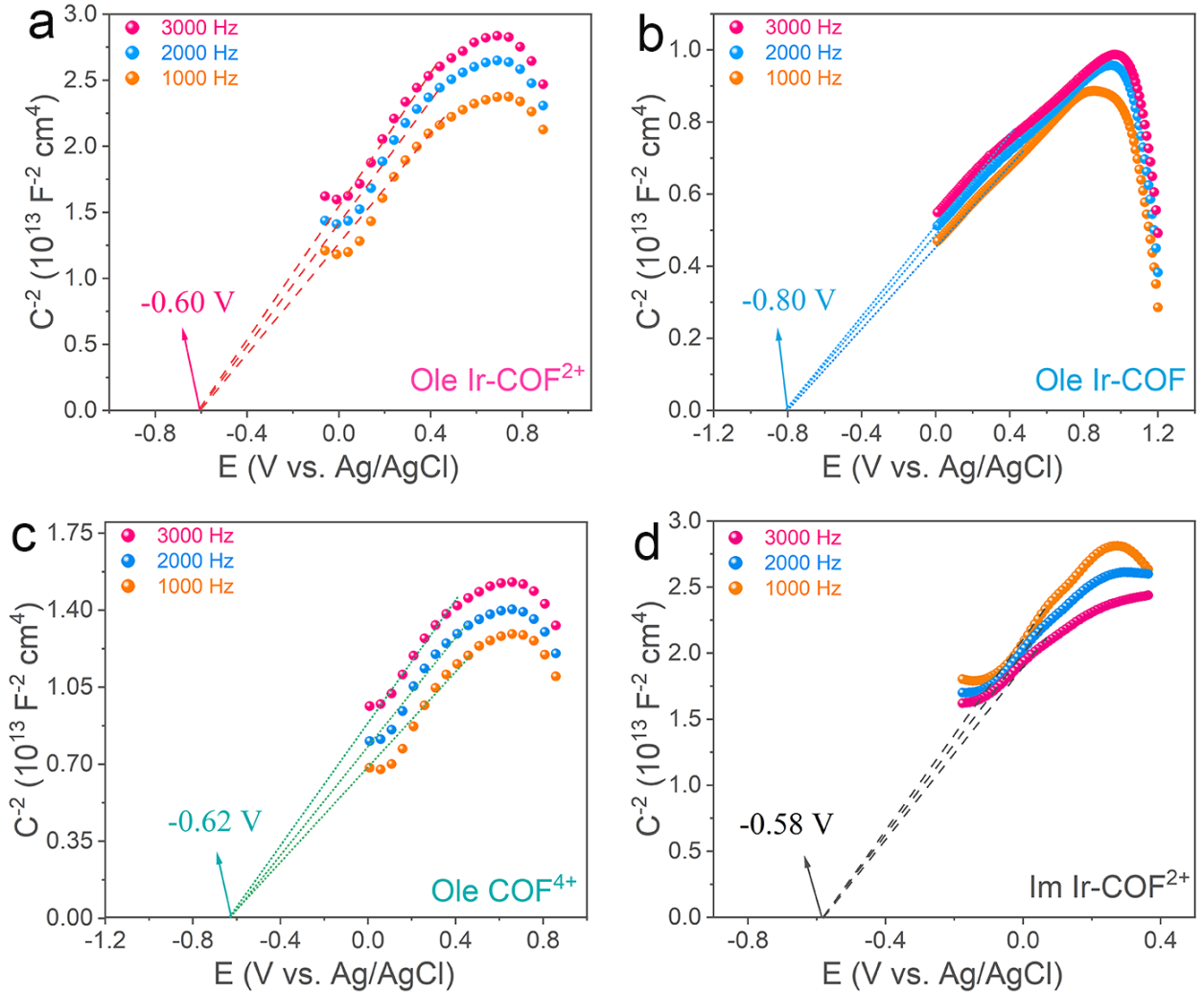


**Figure S22.** Mott-Schottky plots of PQ-TPM at 1, 2, and 3 kHz frequency. The flat-band potential of (a) Ole Ir-COF^2+^, (b) Ole Ir-COF, (c) Ole COF^4+^ and (d) Im Ir-COF^2+^ can be obtained by fitting the x-axis intercept of the linear region of the M-S plots.

**
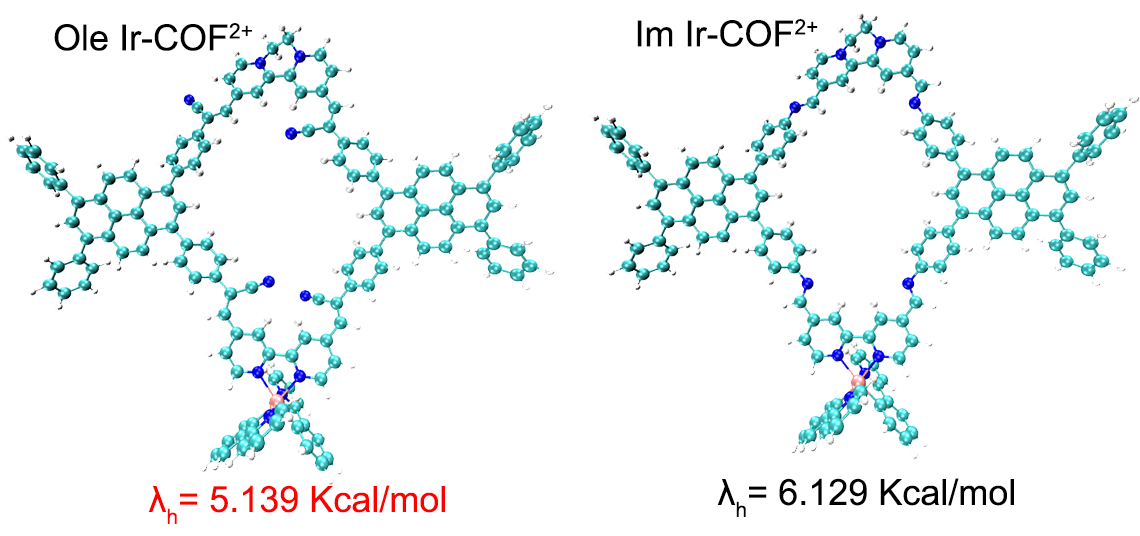
**

**Figure S23.** The charge recombination energy exhibited by Im Ir-COF^2+^ and Ole Ir-COF^2+^


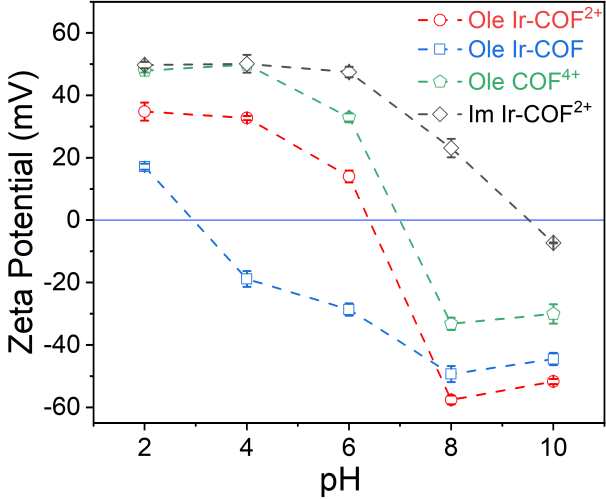


**Figure S24.** Zeta potentials of Ole Ir-COF^2+^, Ole Ir-COF, Ole COF^4+^ and Im Ir-COF^2+^. Zeta potential measurements determined the zero charge point (pHpzc) of Ole Ir-COF^2+^ to be 6.4, indicating that the surface carries a positive charge below pH 6.4 and a negative charge above pH 6.4.


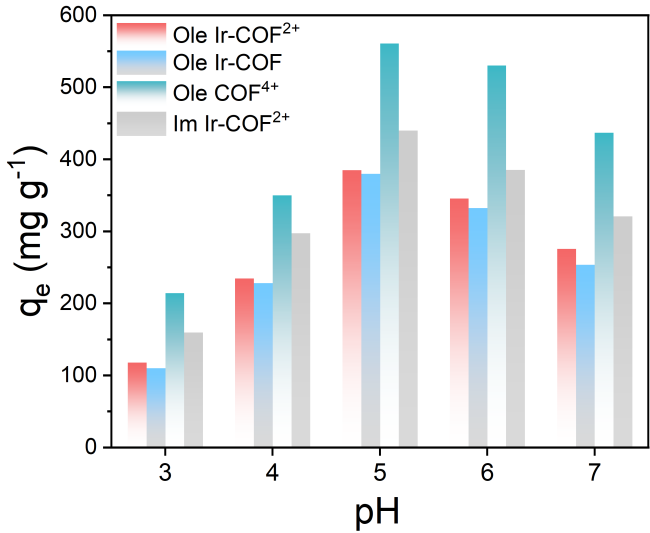


**Figure S25.** The effect of pH on the uptake of Pd(II) by Ole Ir-COF^2+^, Ole Ir-COF, Ole COF^4+^ and Im Ir-COF^2+^. As is well known, Pd(II) mainly exists in the form of chloride complexes (e.g., PdCl_4_^2-^), and its form is closely related to the solution pH. At low pH, excess H^+^ can form neutral complexes with PdCl_4_^2-^, weakening its affinity for adsorption sites. As the pH increases, Pd(II) species gradually transform into hydroxylated forms^9^. Consequently, the pH of the solution critically regulates both the adsorbent's charge state and Pd(II) speciation, significantly influencing adsorption performance.


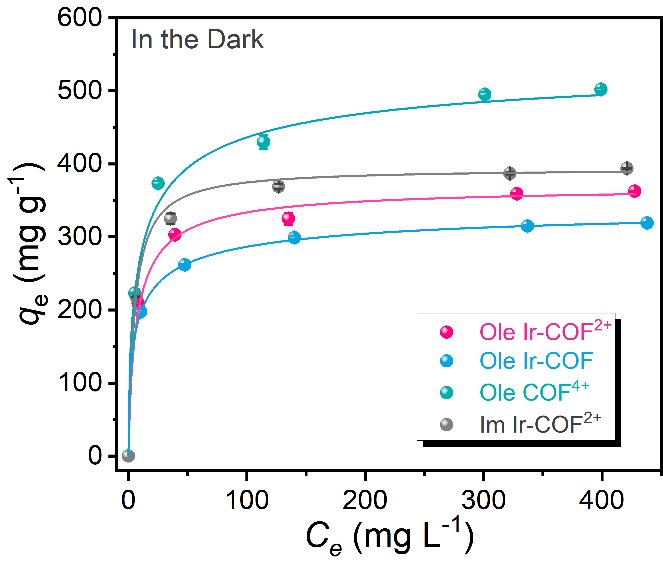


**Figure S26.** In the dark, equilibrium adsorption isotherms for PdCl_4_^-^ adsorption on Ole Ir-COF^2+^, Ole Ir-COF, Ole COF^4+^ and Im Ir-COF^2+^ at a fixed material-to-solution ratio of 0.2 mg L^−1^ in Pd-spiked water (from 50 to 500 ppm), pH=5.


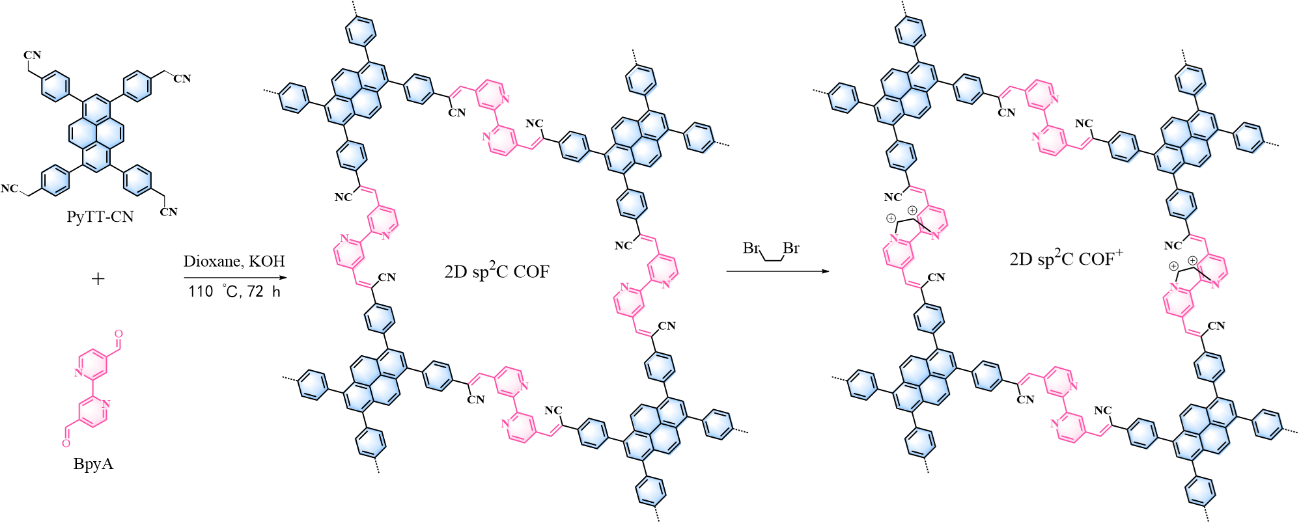


**Figure S27.** Synthetic scheme of the 2D sp^2^C COF and local cationized 2D sp^2^C COF^+21, 22, 23^. A 2D sp²c-COF was constructed. Subsequent quaternization yielded a COF containing cyclic diquat structures, named 2D sp²c-COF^+^.


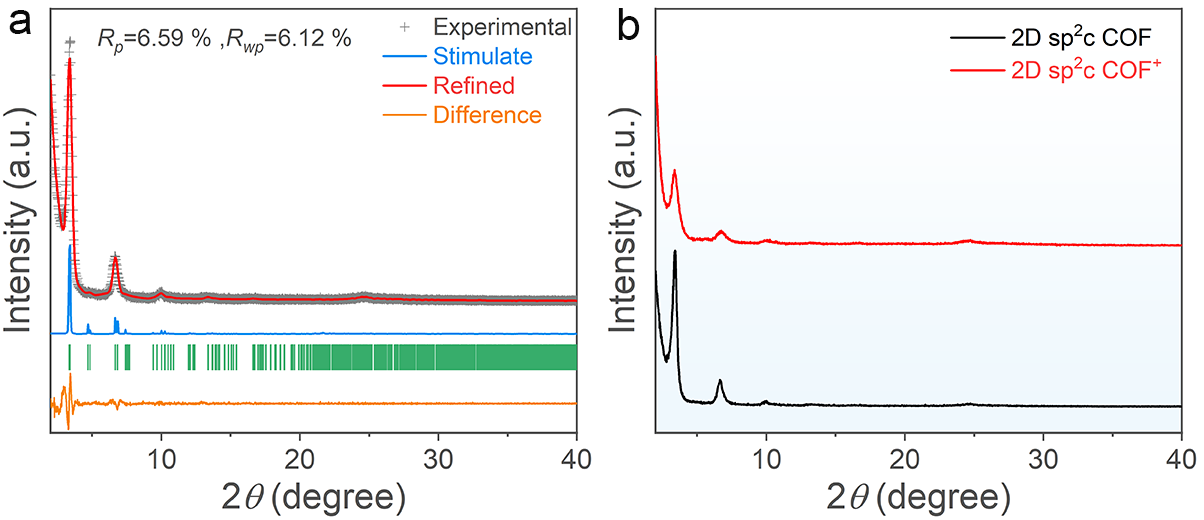


**Figure S28.** (a) Experimental (black) and Pawley-refined (red) PXRD patterns with simulated AA-stacking structures for 2D sp^2^c COF. Bragg positions (green), difference curves (orange), and AA-stacking simulations (blue) are shown. (b) The XRD spectra of 2D sp^2^c COF and 2D sp^2^c COF^+^ (after treatment with quaternization).


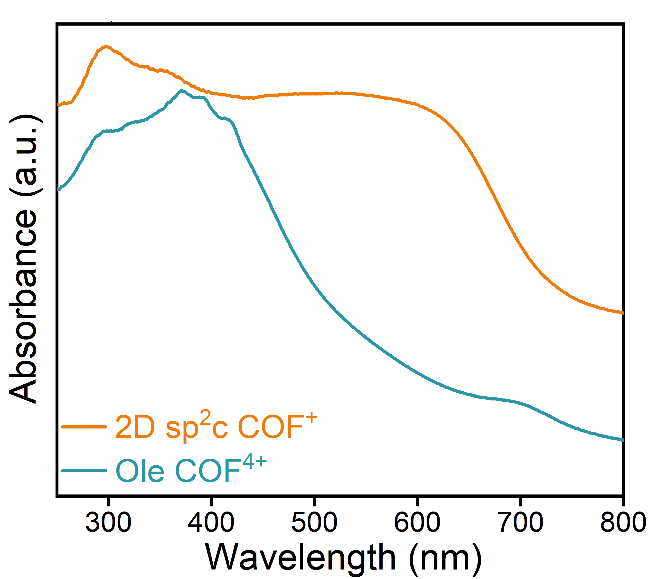


**Figure S29.** UV spectra of 2D sp^2^c COF^+^ and Ole COF^4+^. UV spectra showed that, compared with 1D COF (Ole COF^4+^), the absorption peak of 2D sp²c-COF^+^ in the visible region of 400-600 nm was distinctly bathochromic shifted. This is attributed to the larger π-conjugated plane of 2D COF, which effectively extends electron delocalization, thereby absorbing and utilizing photons of shorter wavelengths (higher energies).


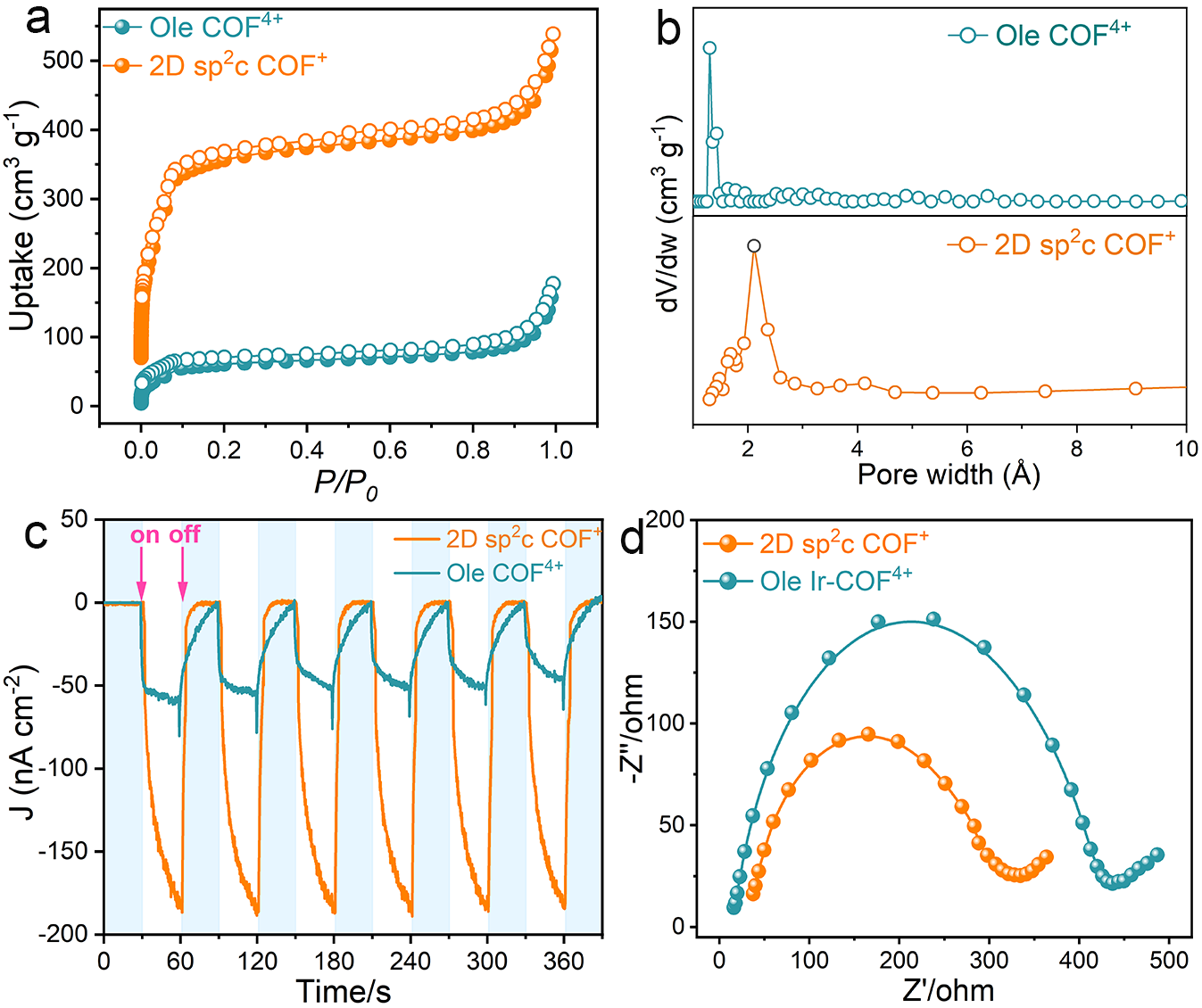


**Figure S30.** (a) N_2_ adsorption/desorption isotherms and (b) pore size distribution of 2D sp^2^c COF^+^ and Ole COF^4+^. (c) TPR and (d) EIS of 2D sp^2^c COF^+^ and Ole COF^4+^. In contrast to Ole COF^4+^, the 2D sp²c-COF exhibits a higher specific surface area with an ordered, open pore structure that enhances active site exposure and mass transfer, as well as superior charge separation and migration capabilities as confirmed by transient photocurrent and impedance spectroscopy measurements.


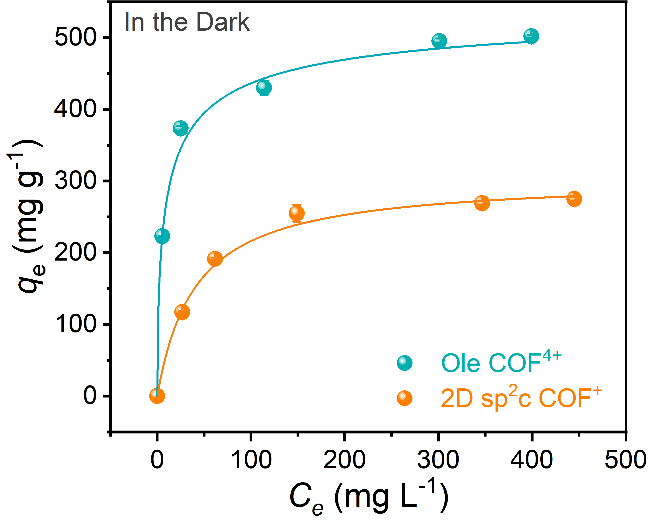


**Figure S31.** In the dark, equilibrium adsorption isotherms for PdCl_4_^2-^ adsorption on different materials at a fixed material-to-solution ratio of 0.2 mg L^−1^ in Pd-spiked water (from 50 to 500 ppm). Owing to the high accessibility of edge active sites in Ole-COF^4+^, its dark adsorption capacity for PdCl_4_^2-^ reached 4.8 times that of the 2D sp^2^c COF^+^.


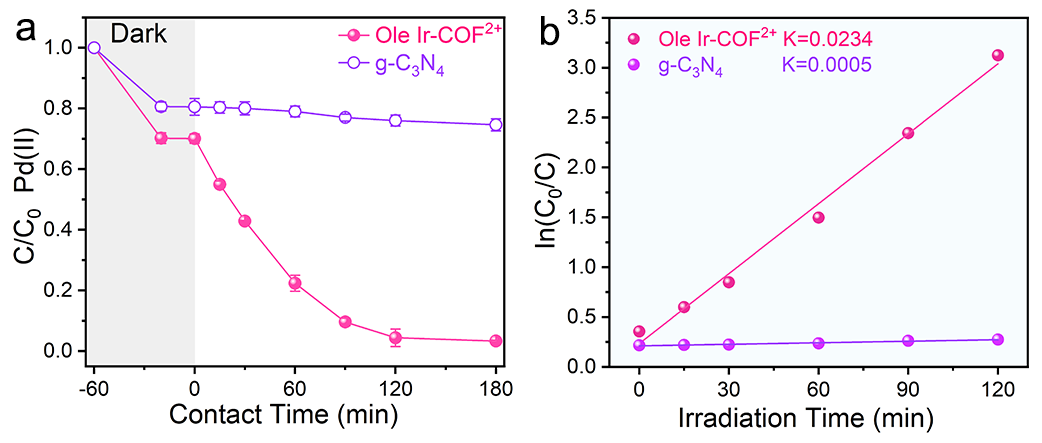


**Figure 32.** (a) The removal kinetics of PdCl_4_^2-^ on Ole Ir-COF^2+^ (pink) and g-C_3_N_4_ (purple). (b) The corresponding pseudo-first-order rate constant (k) of PdCl_4_^2-^ reduction with Ole Ir-COF^2+^ and g-C_3_N_4_. (Adsorption experimental conditions of g-C_3_N_4_: m/V = 1/5, t = 180 min, pH = 5, C_0_ = 100 ppm. The maximum adsorption capacity of g-C_3_N_4_ is 115 mg g^-1^.)


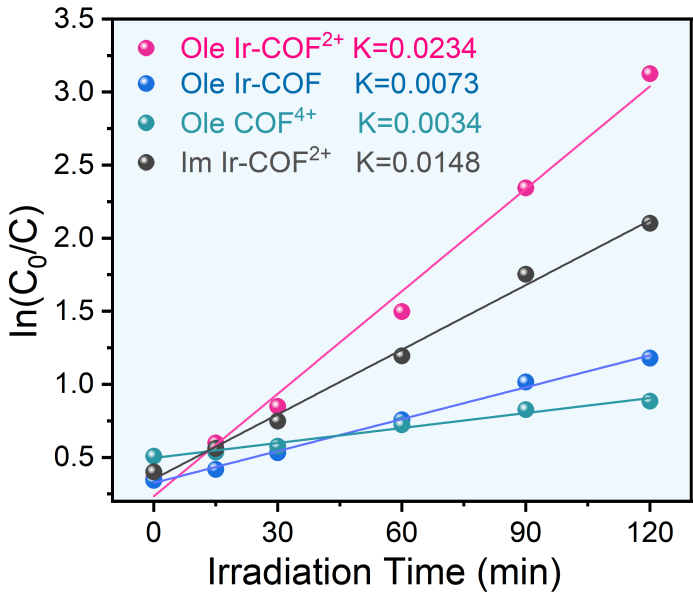


**Figure S33.** The corresponding pseudo-first-order rate constant (k) of PdCl_4_^2-^ reduction with Ole Ir-COF^2+^, Ole Ir-COF, Ole COF^4+^ and Im Ir-COF^2+^ as photocatalysts.


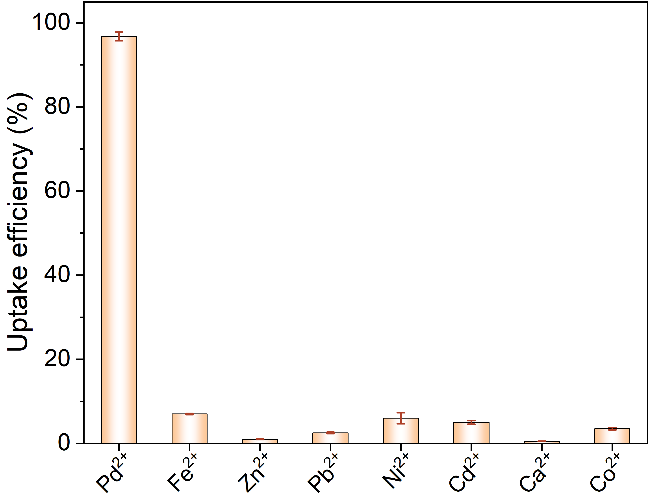


**Figure S34.** Competitive adsorption of coexistent ions by Ole Ir-COF^2+^.


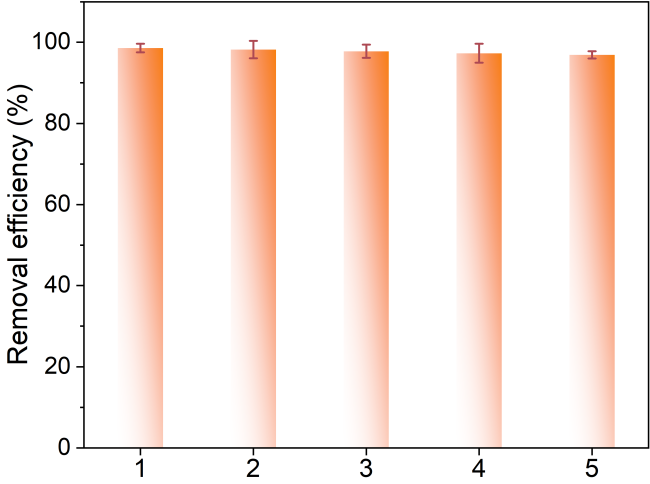


**Figure S35.** The recycle performance of Ole Ir-COF^2+^ for Pd(II) reduction showing just neglectable variety.


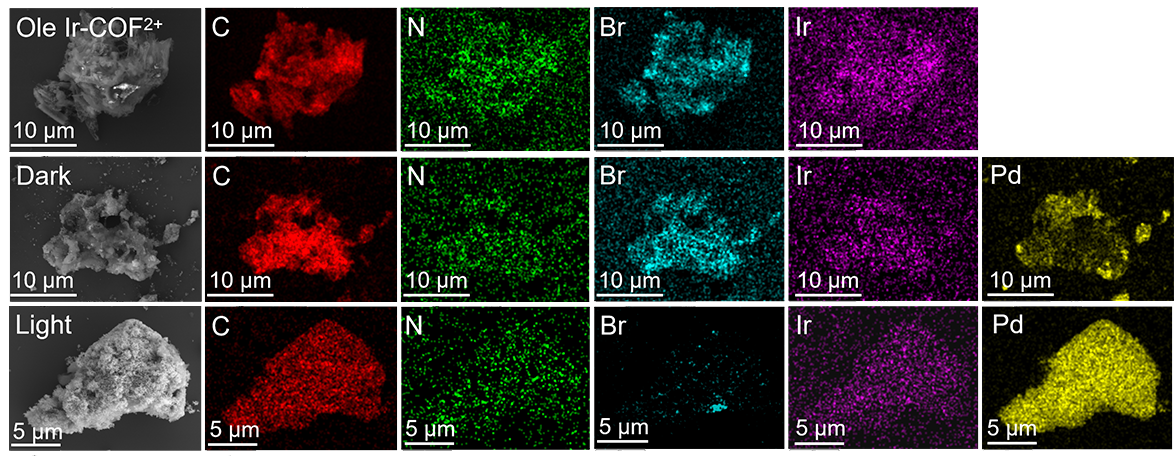


**Figure S36.** SEM and EDS mapping images of Ole Ir-COF^2+^ before adsorption and after adsorption of PdCl_4_^2-^ in the dark and under light.


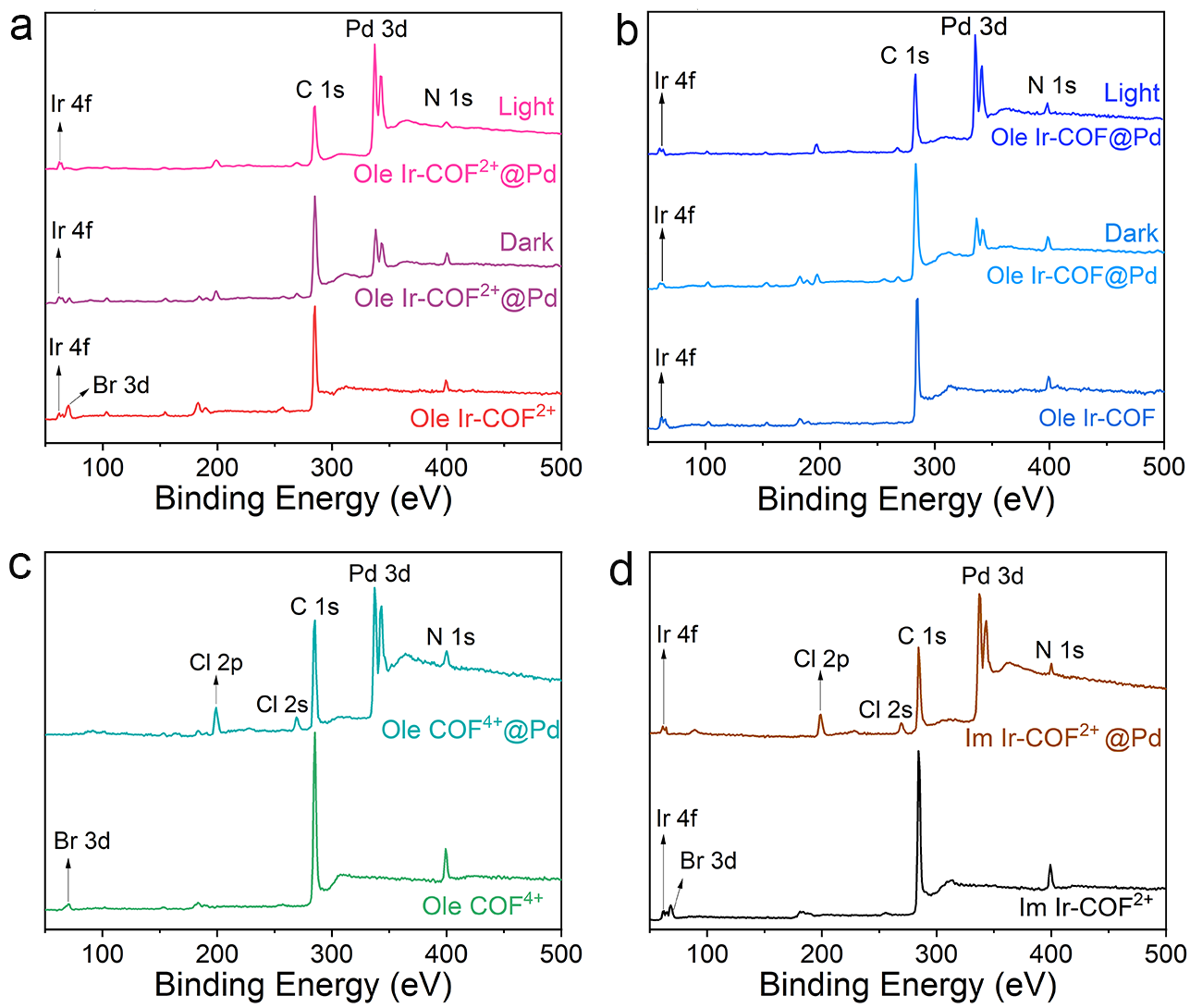


**Figure S37.** Comparison of normalized XPS survey spectra of (a) Ole Ir-COF^2+^, (b) Ole Ir-COF in its pristine state, after palladium loading under dark conditions, and after palladium loading under visible light irradiation. XPS survey spectra of (c) Ole COF^4+^ and (d) Im Ir-COF^2+^ before and after treatment with palladium under light condition. Appearance of Pd 3d peaks confirms Pd successful loaded onto Ole Ir-COF^2+^, Ole Ir-COF, Ole COF^4+^ and Im Ir-COF^2+^.


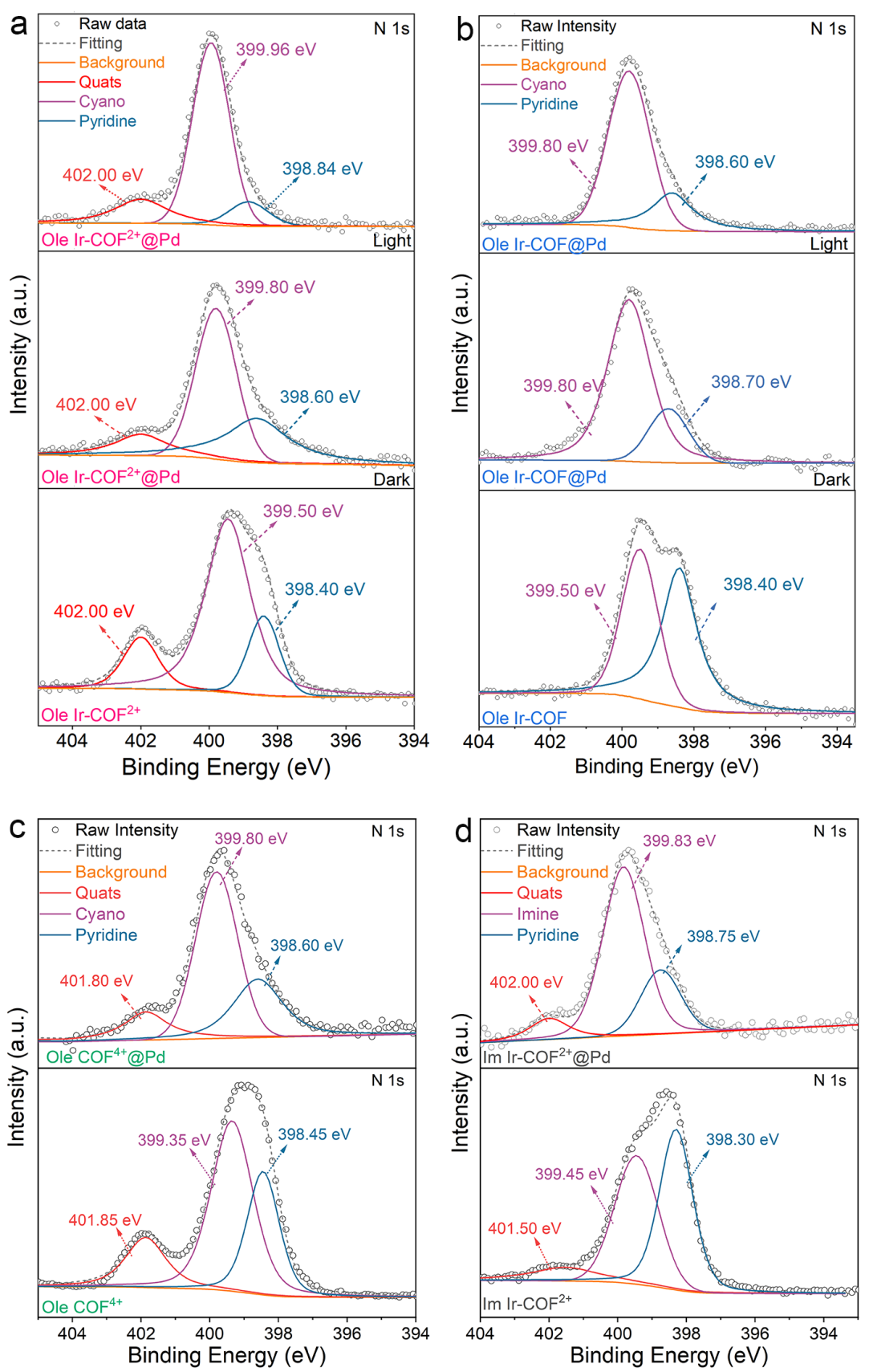


**Figure S38.** XPS characterization of photoreduced Pd(II) on four COFs. The N1s XPS spectra of (a) Ole Ir-COF²⁺, (b) Ole Ir-COF in its original state, after palladium loading under dark conditions, and after palladium loading under visible light illumination. The N1s XPS spectra of (c) Ole COF^4+^ and (d) Im Ir-COF^2+^ before and after treatment with palladium under visible light. Ole Ir-COF after photocatalytic Pd(II) recovery was named Ole Ir-COF@Pd. Ole COF^4+^ after photocatalytic Pd(II) recovery was named Ole COF^4+^@Pd. Im Ir-COF after photocatalytic Pd(II) recovery was named Im Ir-COF^2+^@Pd.


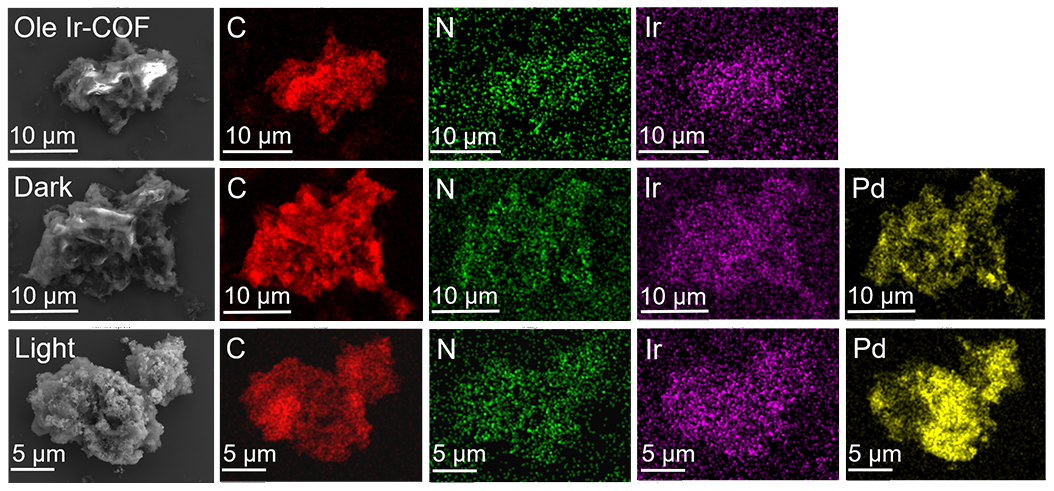


**Figure S39**. SEM and EDS mapping images of Ole Ir-COF before adsorption and after adsorption of PdCl_4_^2-^ in the dark and under light.


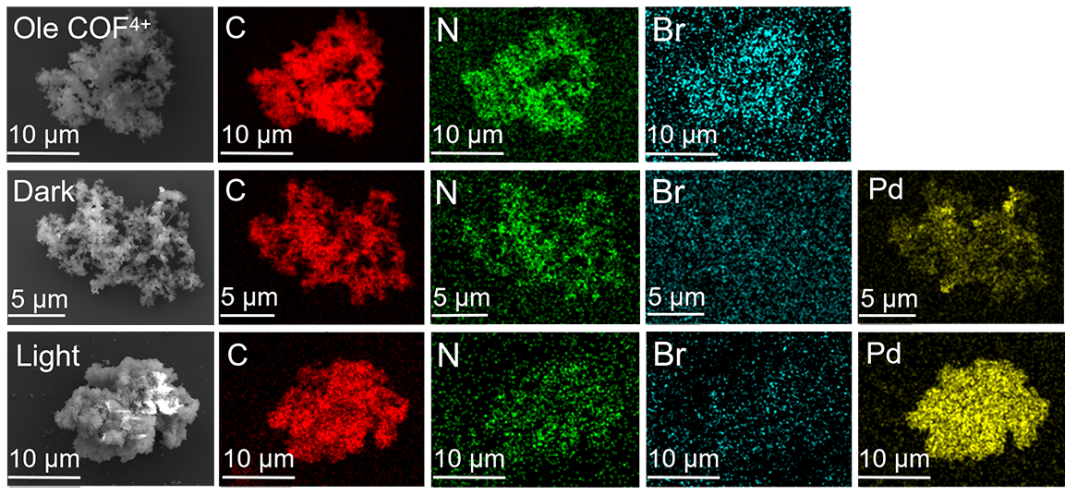


**Figure S40.** SEM and EDS mapping images of Ole COF^4+^ before adsorption and after adsorption of PdCl_4_^2-^ in the dark and under light.


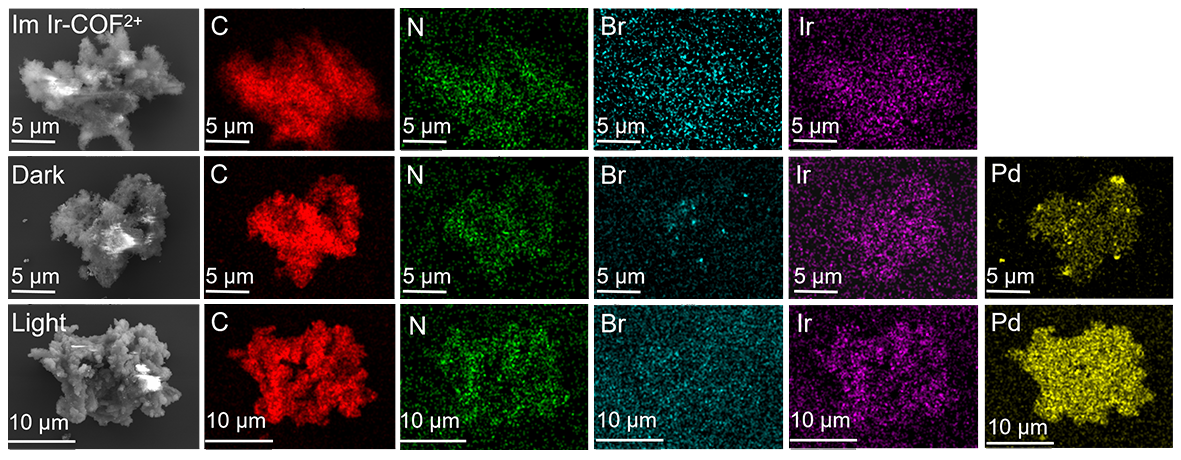


**Figure S41.** SEM and EDS mapping images of Im Ir-COF^2+^ before adsorption and after adsorption of PdCl_4_^2-^ in the dark and under light.


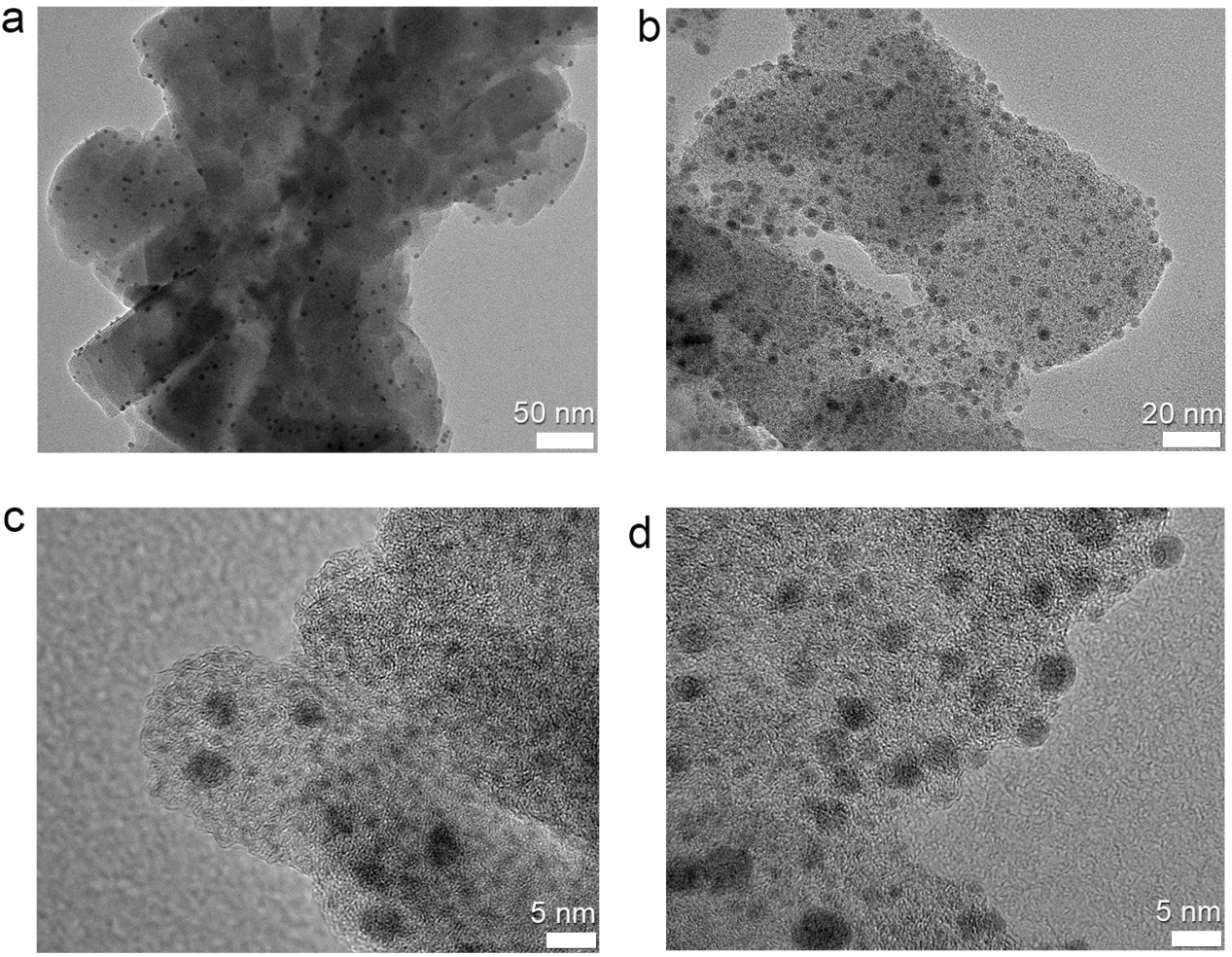


**Figure S42**. HR-TEM images of Ole Ir-COF^2+^@Pd.


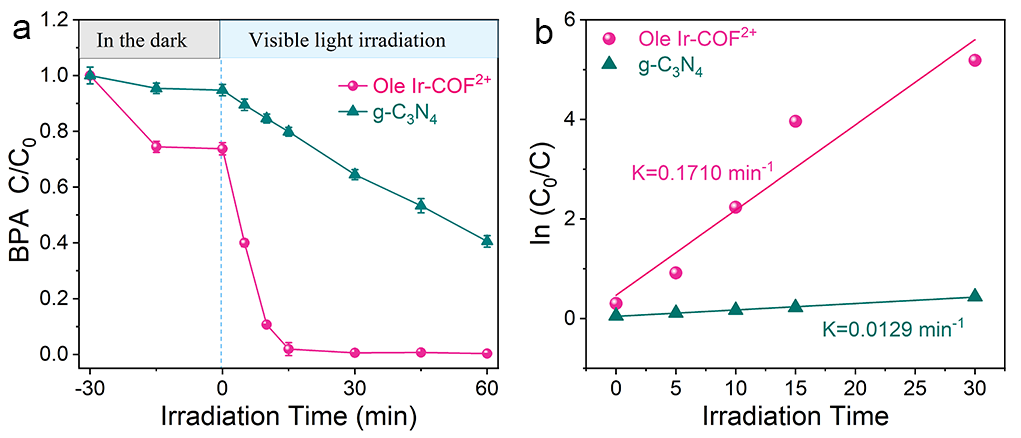


**Figure S43.** (a) The removal kinetics of BPA on Ole Ir-COF^2+^ and g-C_3_N_4._ (b) The g-C_3_N_4_ degradation rate constant of BPA.


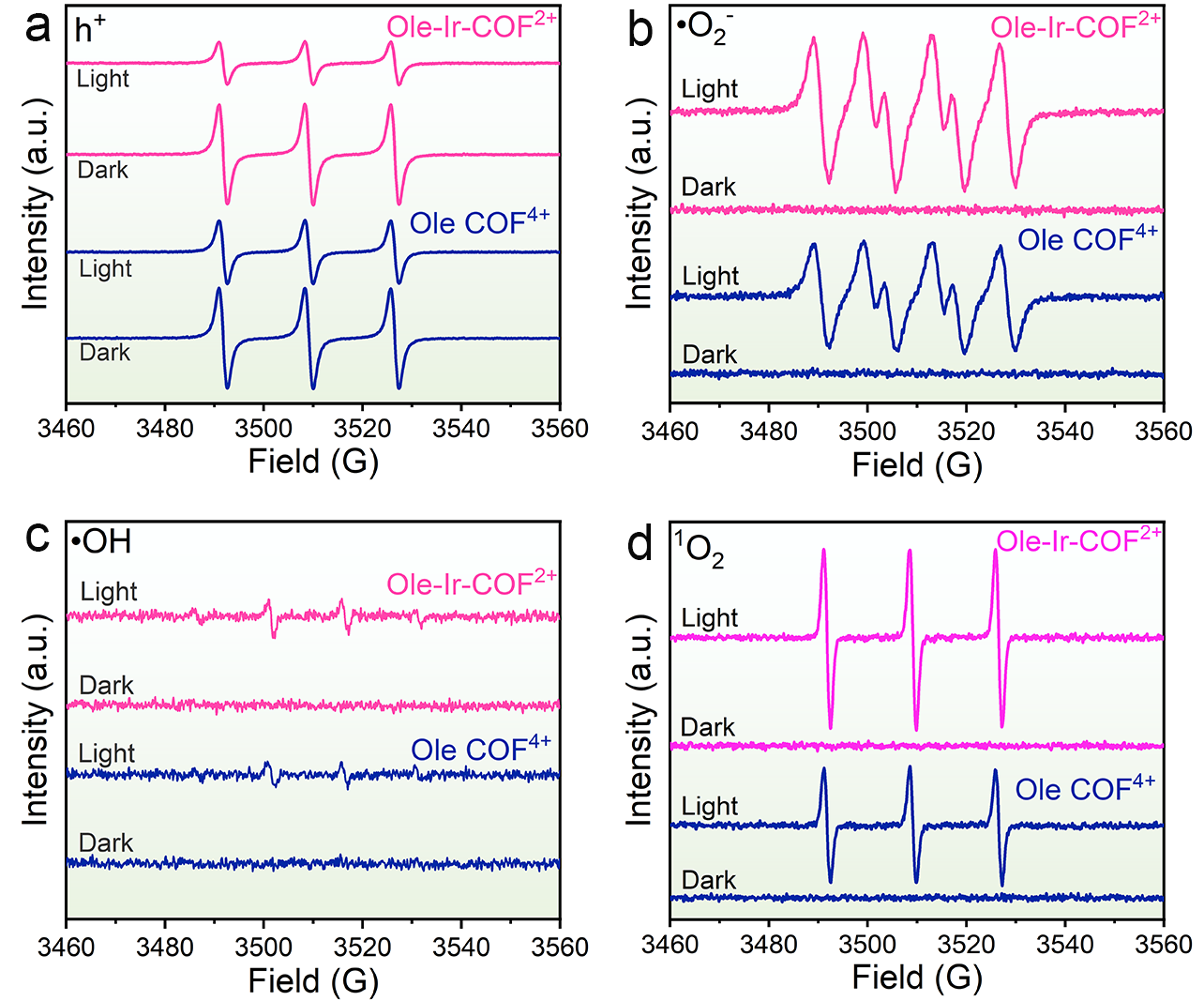


**Figure S44.** Spin-trapping EPR spectra for reactive species in Ole Ir-COF^2+^ and Ole COF^4+^ with and without light: (a) h^+^, (b) •O_2_^-^, (c) •OH, (d) ^1^O_2_.


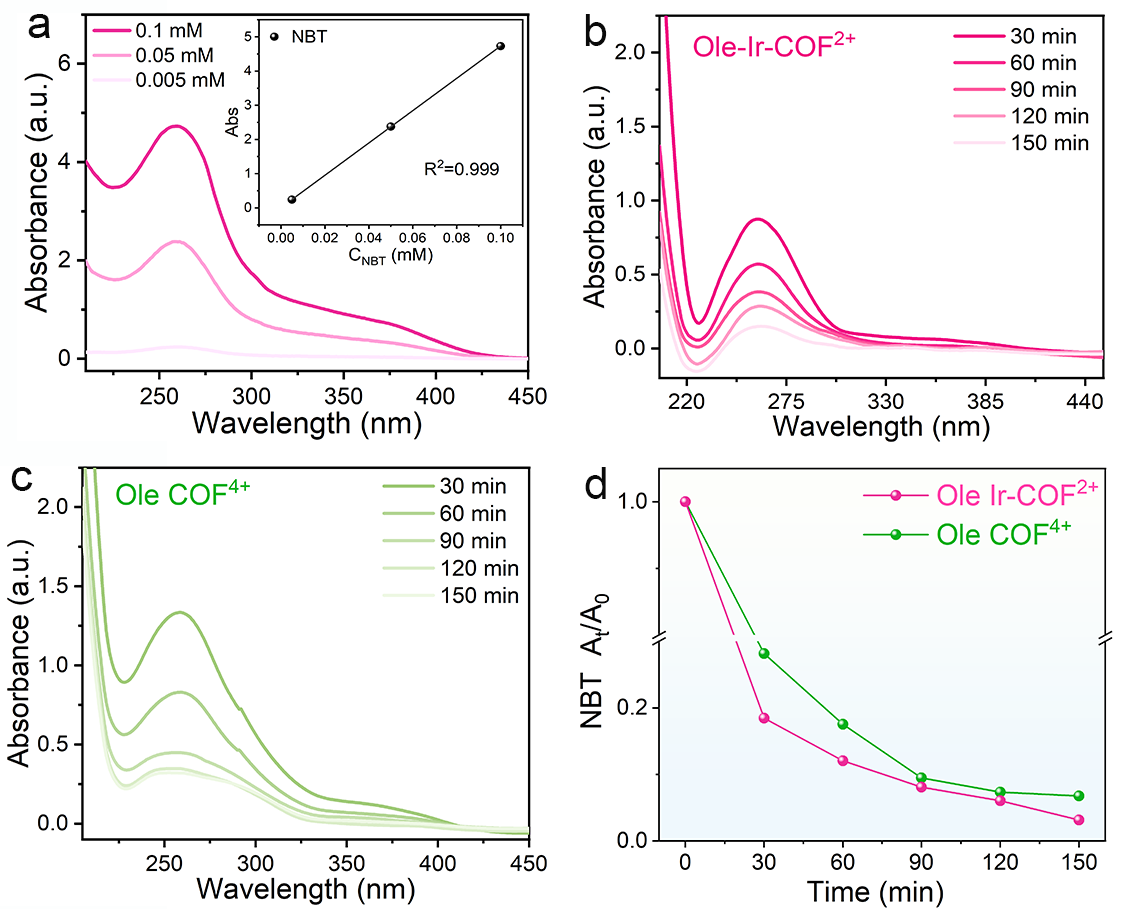


**Figure S45.** (a) UV-vis absorbance spectra of NBT solution at different concentrations. (b, c) UV-vis spectra of NBT solution over Ole Ir-COF^2+^ and Ole COF^4+^ at different time under 300 W Xe lamp. (d) Detection of O_2_^•−^ using NBT in Ole Ir-COF^2+^ and Ole COF^4+^ photocatalytic systems.

***Note 6:*** The formation of O_2_^•−^ was detected using the nitro blue tetrazolium (NBT) assay. O_2_^•−^ can reduce NBT to insoluble purple formazan. Typically, nitro blue tetrazolium (NBT) was ultrasonically dispersed in phosphate buffered saline (PBS) (pH=7) to prepare an NBT solution with an initial concentration of 0.1 mM. Then, the COF sample was added to 5 mL of NBT PBS solution (0.1 mM) in a quartz test tube. The resulting mixture was stirred well and irradiated under a 300 W xenon lamp for 3 h. Subsequently, the precipitate was filtered out using a 0.22 μm polyvinylidene fluoride (PVDF) membrane filter before UV-visible measurement. The filtrate containing NBT was diluted to achieve the best optical reading. A standard curve can be obtained based on the absorbance of NBT standard solutions with different concentrations. Assuming that the reaction molar ratio of NBT to O_2_^•−^ is 1:4^24^, the generation of O_2_^•−^ is quantified by measuring the absorbance change of the characteristic signal of NBT at 259 nm. Therefore, the concentration of O_2_^•−^ can be determined by calculating the concentration change of NBT degraded by COF samples at different times.


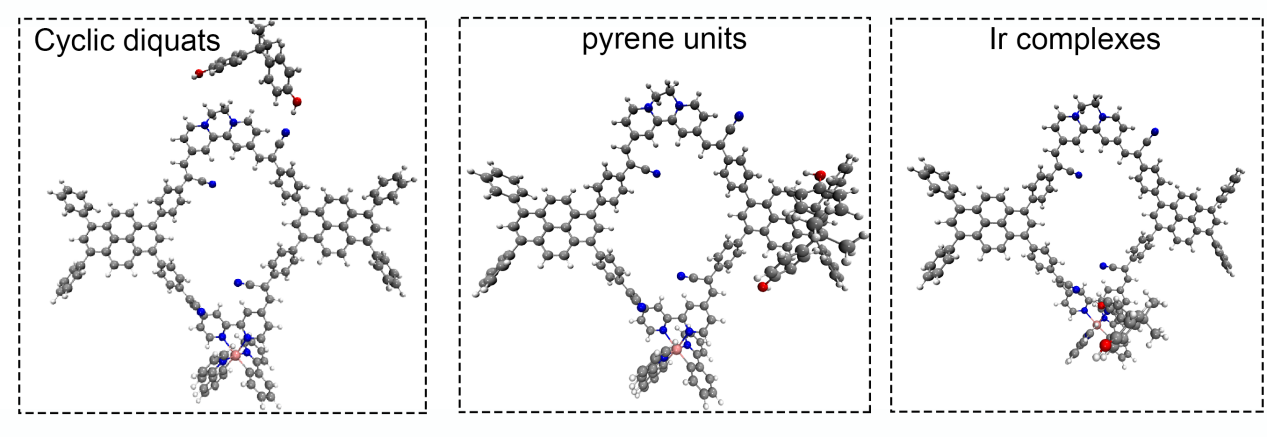


**Figure S46.** Calculation model of adsorption energy of BPA molecules at different sites on Ole Ir-COF^2+^.


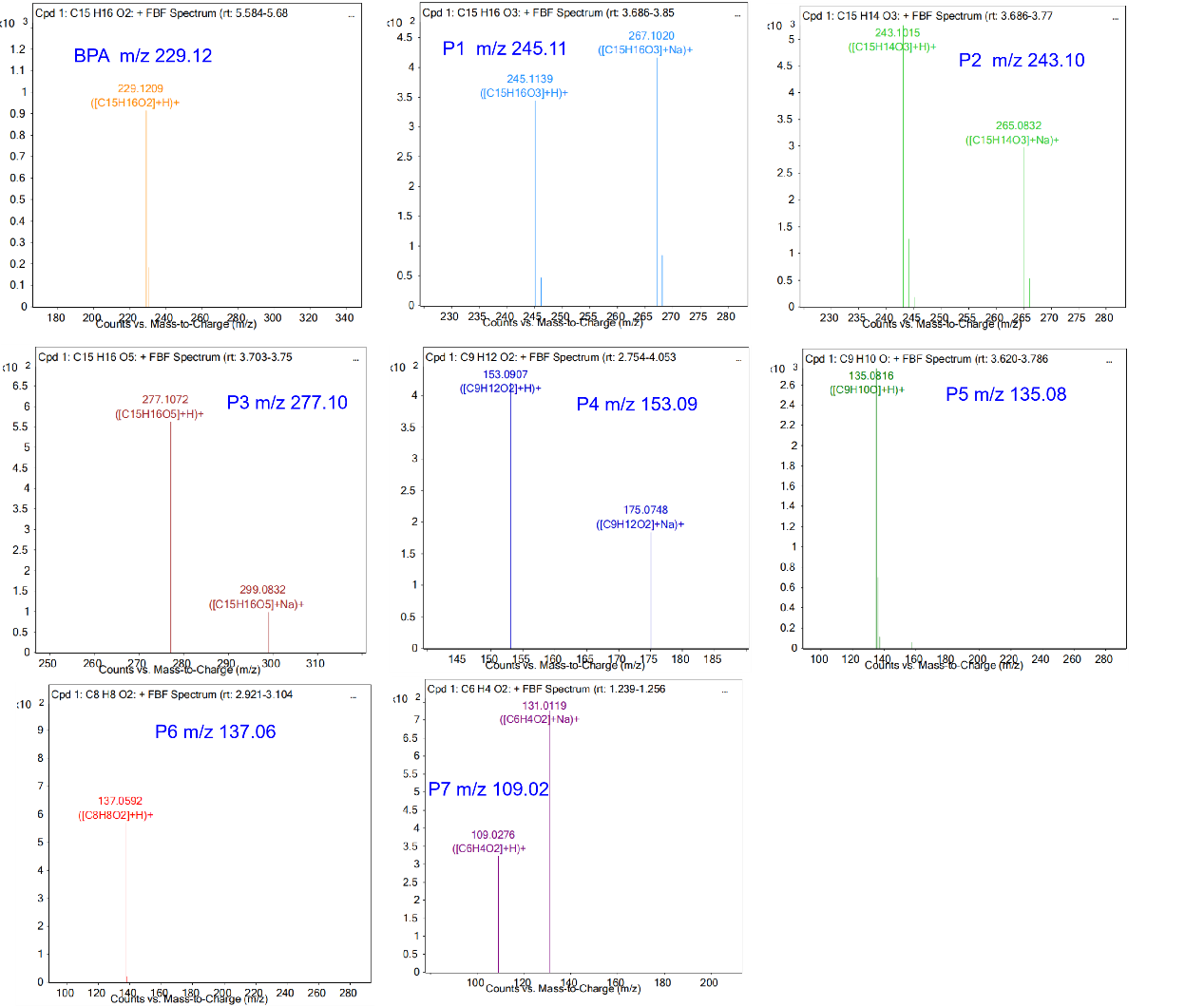


**Figure S47.** LC/MS chromatogram and mass spectra for BPA. Mass spectra of the main peaks. Routine conditions: [BPA] = 50 ppm, [catalyst] = 0.2 mg L^-1^, temperature = 25 °C, and initial pH=5^25^.

***Note 7:*** The main pathway for the degradation of BPA by Ole Ir-COF^2+^ under visible light irradiation is also through hydroxylation and ring cleavage. In pathway 1, carbon atoms with high f^0^ values ​are more susceptible to free radical attack and electrophilic attack, and BPA is easily attacked by hydroxyl radicals to form monohydroxylated byproduct P1 (m/z 245)^26^. Further oxidized by •O_2_^-^ radicals, P1 can be converted into quinone structure product P2 (m/z 243), which is then oxidized to form carboxylic acid product P3 (m/z 277)^27^. At the same time, product P2 may form byproduct P4 (m/z 153) through ring cleavage. In pathway 2, the aromatic ring is directly attacked by photogenerated h^+^, and the β-C bond is broken due to the low f0 value of the C atom between the two benzene rings of BPA, resulting in primary product P4, which is converted into product P5 (m/z 135) through elimination reaction. The removal of the hydroxyl group (-OH) is accompanied by the elimination of β-hydrogen (β-H) to form a new unsaturated double bond. This process conforms to the classical *E1* or *E2* elimination reaction mechanism, while releasing water molecules (H₂O) as a by-product. These products are subsequently oxidized to form intermediate P6 (m/z 137). Thereafter, the intermediate can be catalytically rearranged to benzoquinone P7 (m/z 109) by active species. Hydroxyl oxidation-dehydrogenation mechanism The hydroxyl group (-OH) of the intermediate P6 (m/z 137) undergoes oxidative dehydrogenation under the action of active oxygen species to form a keto group (C=O). This step may be accompanied by the elimination of β-hydrogen of the adjacent carbon (E1cb or free radical mediated) to form a conjugated double bond structure (quinone skeleton). Ultimately, the aromatic compounds progress to the ring-opening intermediates during the photocatalytic degradation process and are ultimately mineralized into carbon dioxide and water.

**
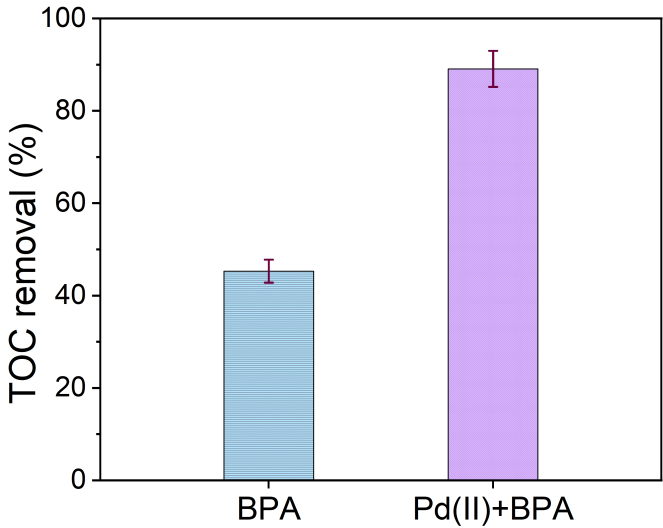
**

**Figure S48.** After 120 minutes of illumination, the TOC removal rates of Ole Ir-COF^2+^ on the single BPA system and the Pd(II)+BPA system were compared.


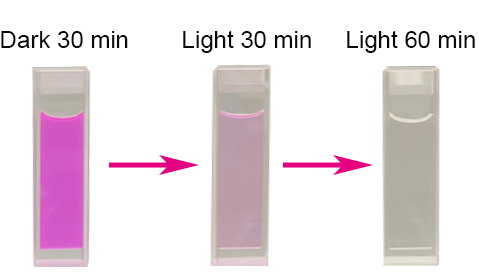


**Figure S49.** Color change of Ole Ir-COF^2+^ before and after photocatalytic degradation of contaminant RhB. After 1 h of illumination, the initially pink RhB solution became colorless, confirming its complete degradation.


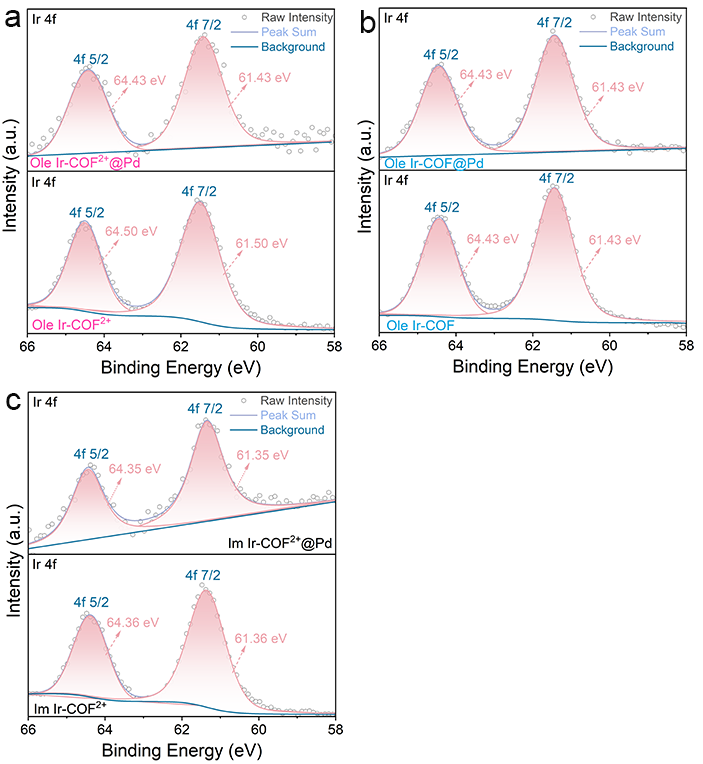


**Figure S50.** The Ir 4f XPS spectra of (a) Ole Ir-COF^2+^, (b) Ole Ir-COF, and (c) Im Ir-COF^2+^ before and after treatment with palladium under visible light. Obviously, after palladium binding under light irradiation, the two Ir 4f peaks of Ole Ir-COF^2+^, Ole Ir-COF, and Im Ir-COF^2+^ have no obvious shift changes, further indicating that the Ir atoms in Ole Ir-COF^2+^, Ole Ir-COF, and Im Ir-COF^2+^ do not participate in the coordination or adsorption of Pd.


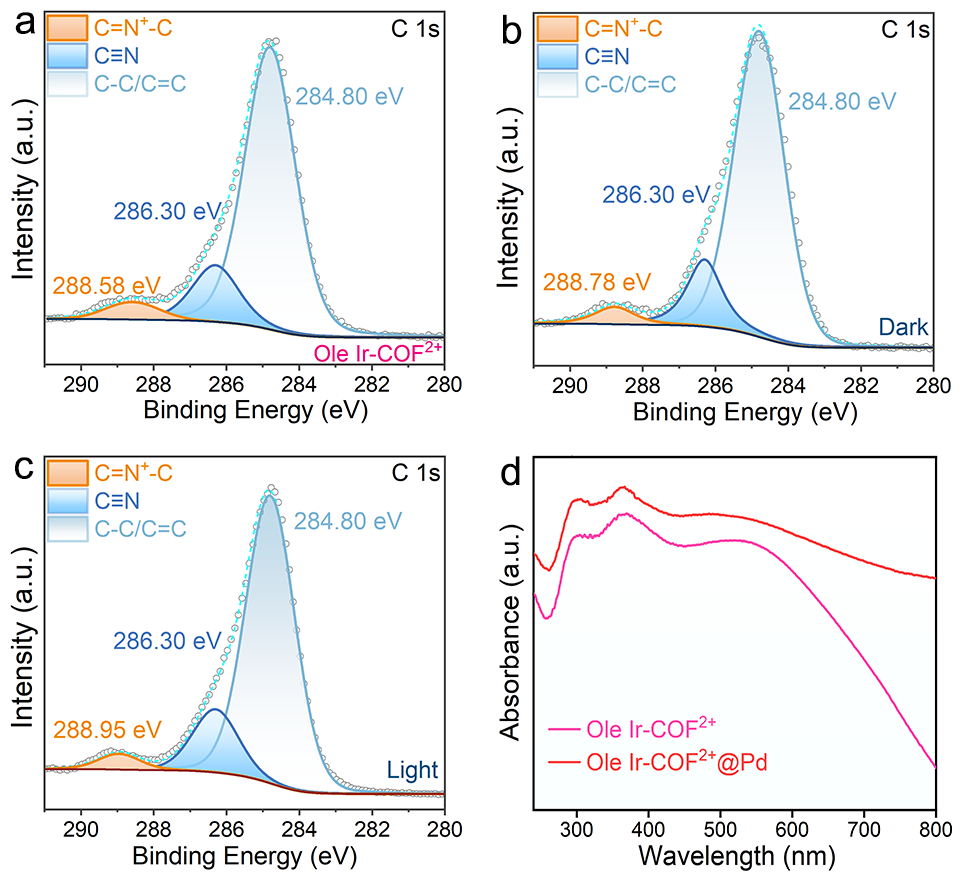


**Figure S51 .**The C 1s XPS spectra of Ole Ir-COF^2+^, (a) before and after treatment with palladium under (b) dark and (c) visible light. (d) The UV-vis spectra of Ole Ir-COF^2+^  before and after treatment with palladium under visible light.

**Table S11.** XPS of C for fresh, after absorbed, after irradiation.

| **XPS of C 1s** | | **Fresh** | | | **After absorbed** | | | **After irradiation** | | |
| --- | --- | --- | --- | --- | --- | --- | --- | --- | --- | --- |
| **Ole Ir-COF^2+^** | Binding energy (ev) | 284.80 | 286.30 | 288.58 | 284.80 | 286.30 | 288.78 | 284.80 | 286.30 | 288.95 |
|  | Area (%) | 79.56 | 15.64 | 4.80 | 79.75 | 15.69 | 4.56 | 79.27 | 16.76 | 3.97 |
|  | FWHM (eV) | 1.63 | 1.48 | 1.73 | 1.62 | 1.12 | 1.30 | 1.58 | 1.47 | 1.34 |


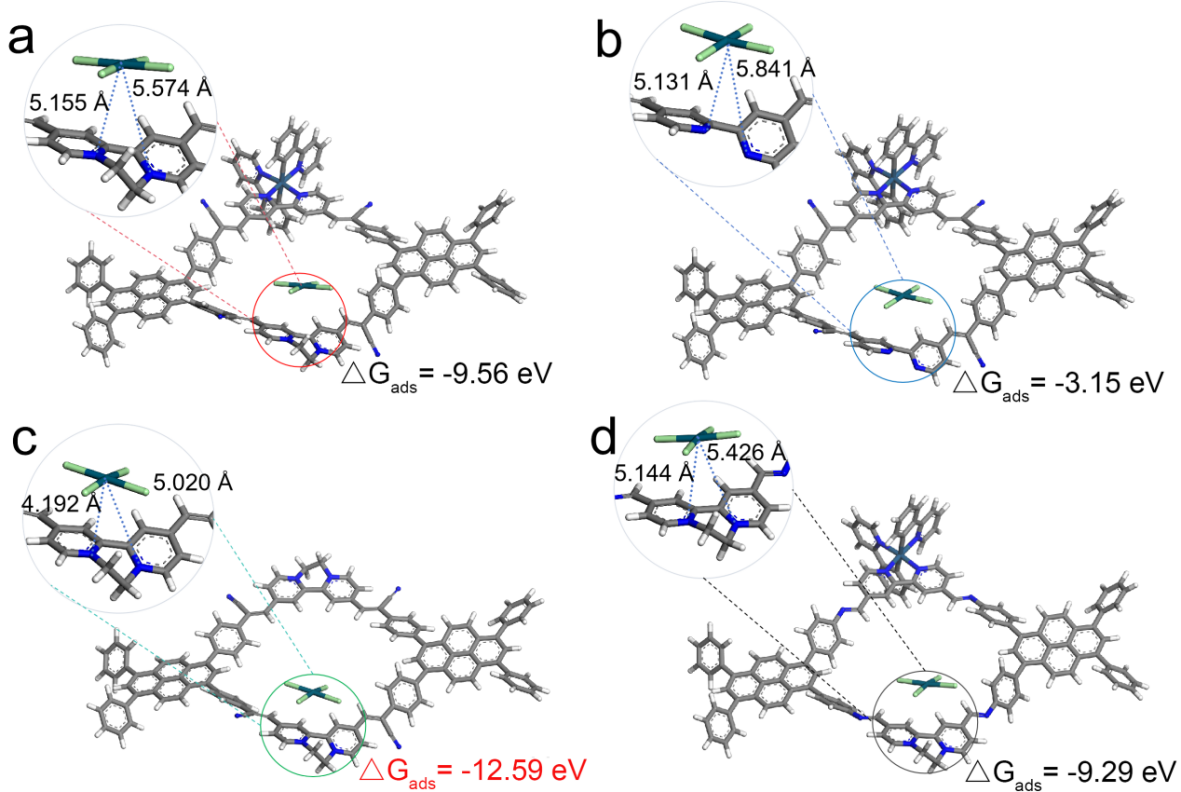


**Figure S52.** The optimized structure of the complexes and the binding energy, bipyridine adsorption sites of PdCl_4_^2-^ on (a) Ole Ir-COF^2+^, (b) Ole Ir-COF, (c) Ole COF^4+^ and (d) Im Ir-COF^2+^.


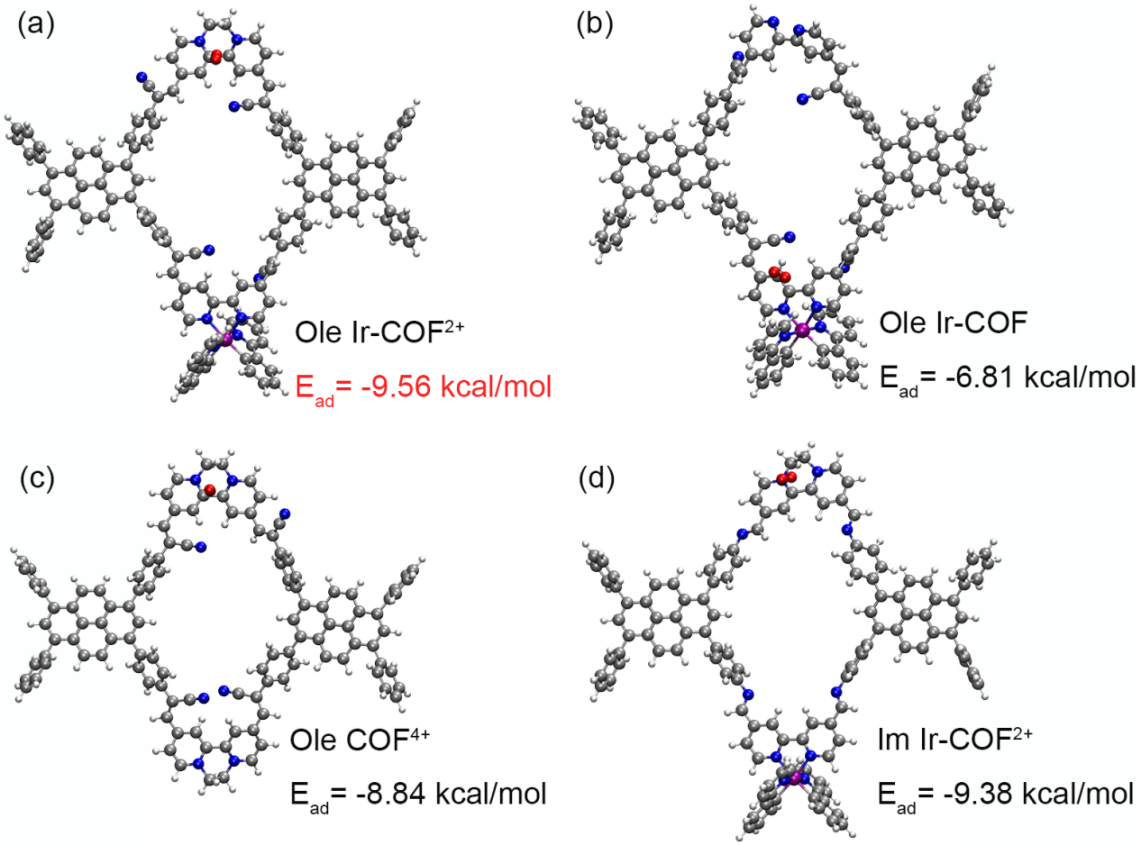


**Figure S53.** Optimized structures of the complexes in the first excited state, and the binding energy of O_2_ at the electron accumulation site of (a) Ole Ir-COF^2+^, (b) Ole Ir-COF, (c) Ole COF^4+^, and (d) Im Ir-COF^2+^.


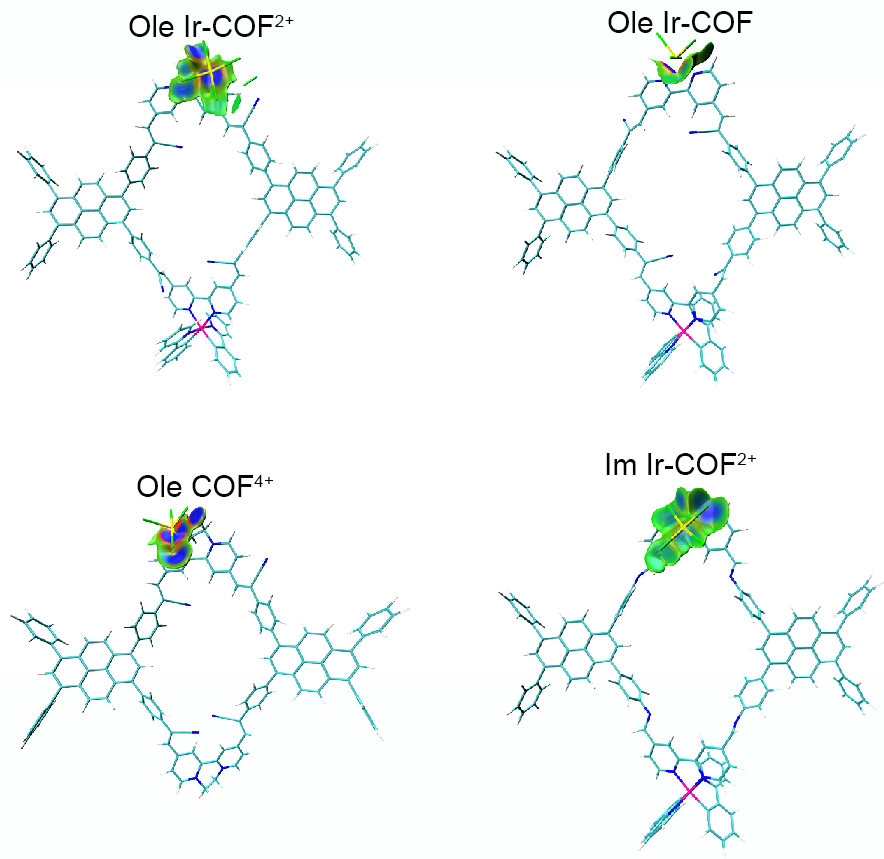


**Figure S54.** IGMH analysis of (a)Ole Ir-COF²⁺, (b) Ole Ir-COF, (c) Ole COF^2+^, (d) Im Ir-COF^2+^ isosurfaces are colored by sign(λ₂) ρ (δginter = 0.005 a.u.).


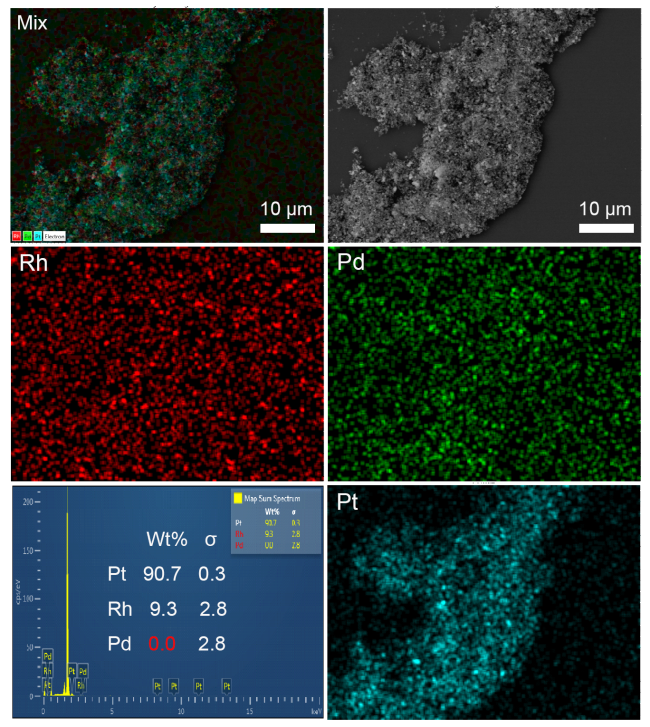


**Figure S55.** The EDS signal of Ole Ir-COF^2+^ disappeared completely after leaching.


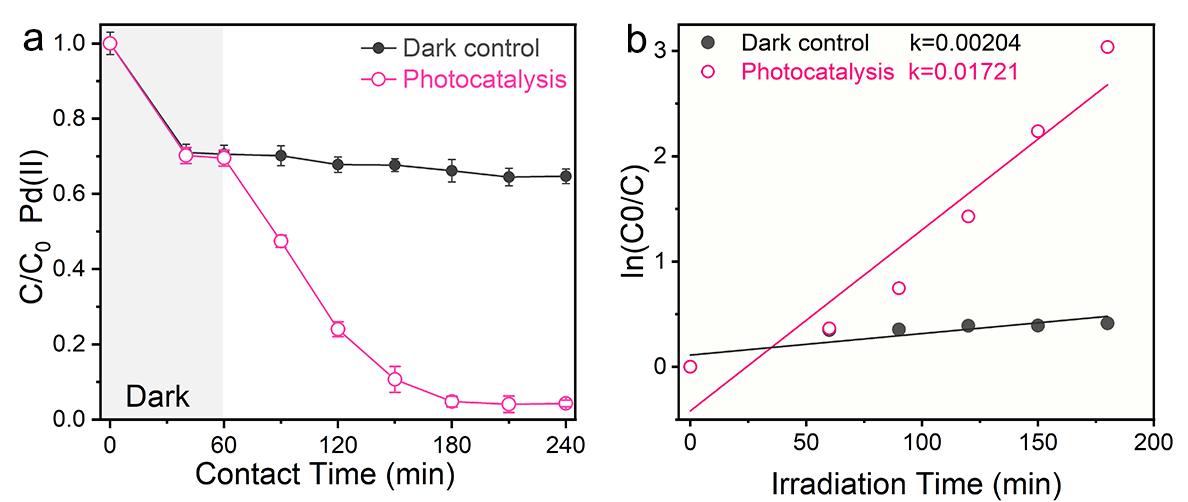


**Figure S56.** The EDS signal of Ole Ir-COF^2+^ disappeared completely after leaching. The contribution rate of COF to the total degradation rate under illumination can be approximately calculated using the following formula:

$$Light Contribution Rate=\frac{k_{Total}-k_{Dark}}{k_{Total}}\times100\%$$

# References

[1] B. Das, S. T. Borah, S. Ganguli, P. Gupta, *Chem. Eur. J.* **2020**, *26*, 14987.

[2] J.-B. Liu, C. Yang, C.-N. Ko, K. Vellaisamy, B. Yang, M.-Y. Lee, C.-H. Leung, D.-L. Ma, *Sensor. Actuat. B-Chem.* **2017**, *243*, 971.

[3] S. Guo, T. Huang, S. Liu, K. Y. Zhang, H. Yang, J. Han, Q. Zhao, W. Huang, *Chem. Sci.* **2017**, *8*, 348.

[4] J. S. Nam, M.-G. Kang, J. Kang, S.-Y. Park, S. J. C. Lee, H.-T. Kim, J. K. Seo, O.-H. Kwon, M. H. Lim, H.-W. Rhee, T.-H. Kwon, *J. Am. Chem. Soc.* **2016**, *138*, 10968.

[5] T. Lu, F. Chen, *J. Comput. Chem.* **2012**, *33*, 580.

[6] W. Humphrey, A. Dalke, K. Schulten, *J. Mol. Graph.* **1996**, *14*, 33.

[7] M. Bashri, S. Kumar, P. Bhandari, S. Stephen, M. J. O’Connor, S. Gaber, T. Škorjanc, M. Finšgar, G. E. Luckachan, B. Belec, E. Alhseinat, P. S. Mukherjee, D. Shetty, *ACS Appl. Mater. Interfaces* **2025**, *17*, 17804.

[8] B. Aguila, Q. Sun, H. C. Cassady, C. Shan, Z. Liang, A. M. Al-Enizic, A. Nafadyc, J. T. Wright, R. W. Meulenberg, S. Ma, *Angew. Chem. Int. Ed.* **2020**, *59*, 19618.

[9] K. S. Song, T. Ashirov, S. N. Talapaneni, A. H. Clark, A. V. Yakimov, M. Nachtegaal, C. Copéret, A. Coskun, *Chem* **2022**, *8*, 2043.

[10] P. Wu, H. Liu, M. Sun, Y. Zeng, J. Ye, S. Qin, Y. Cai, W. Feng, L. Yuan, *J. Mater. Chem. A* **2021**, *9*, 27320.

[11] S. Lin, J. K. Bediako, C.-W. Cho, M.-H. Song, Y. Zhao, J.-A. Kim, J.-W. Choi, Y.-S. Yun, *Chem. Eng. J.* **2018**, *345*, 337.

[12] S. Daliran, M. Ghazagh-Miri, A. R. Oveisi, M. Khajeh, S. Navalón, M. Âlvaro, M. Ghaffari-Moghaddam, H. Samareh Delarami, H. García, *ACS Appl. Mater. Interfaces* **2020**, *12*, 25221.

[13] S.-S. Qin, Z.-K. Wang, L. Hu, X.-H. Du, Z. Wu, M. Strømme, Q.-F. Zhang, C. Xu, *Nanoscale* **2021**, *13*, 3967.

[14] L. Zhao, X. Ma, J. Xiong, Q. Zhou, W. Chen, Z. Yang, F. Jiang, S. Wang, X. Yang, H. Bai, *J. Environ. Chem. Eng.* **2023**, *11*, 110549.

[15] A. Haleem, F. Wu, M. Ullah, T. Saeed, H. Li, J. Pan, *Sep. Purif. Technol.* **2024**, *329*, 125213.

[16] S. Xu, S. Ning, X. Wang, F. Gao, L. Chen, X. Yin, T. Fujita, Y. Wei, *Sep. Purif. Technol.* **2023**, *327*, 124977.

[17] L. Chen, K. Wu, M. Zhang, N. Liu, C. Li, J. Qin, Q. Zhao, Z. Ye, *Chem. Eng. J.* **2023**, *466*, 143082.

[18] Y. Liu, W. Guo, J. Liu, H. Tao, J. Yang, Q. Shuai, Y. Yamauchi, B. Yuliarto, Y. Asakura, L. Huang, *Chem. Sci.* **2025**, *16*, 5745.

[19] Y. Zhao, C. Xu, Q. Qi, J. Qiu, Z. Li, H. Wang, J. Wang, *Chem. Eng. J.* **2022**, *446*, 136823.

[20] Y. Hou, P. Zhou, F. Liu, K. Tong, Y. Lu, Z. Li, J. Liang, M. Tong, *Nat. Commun.* **2024**, *15*, 7350.

[21] Y. Xiang, W. Dong, P. Wang, S. Wang, X. Ding, F. Ichihara, Z. Wang, Y. Wada, S. Jin, Y. Weng, H. Chen, J. Ye, *Appl. Catal. B Environ.* **2020**, *274*, 119096.

[22] Z. Mi, P. Yang, R. Wang, J. Unruangsri, W. Yang, C. Wang, J. Guo, *J. Am. Chem. Soc.* **2019**, *141*, 14433.

[23] S. Shang, Y. Wei, X. Zhao, W. Wang, S. An, H. Li, C. Peng, H. Liu, H. Chen, J. Hu, *Small* **2025**, *21*, e06081.

[24] R. Ma, Y. Zhang, F. Yu, S. Wei, Y. Xing, C. Qiao, Z. Xia, Q. Yang, G. Xie, S. Chen, *ACS Catalysis* **2025**, *15*, 3046.

[25] F. Cheng, J. Wang, *Chem. Eng. J.* **2024**, *484*, 149414.

[26] Y. Tao, Y. Hou, H. Yang, Z. Gong, J. Yu, H. Zhong, Q. Fu, J. Wang, F. Zhu, G. Ouyang, *Proc. Natl. Acad. Sci, U.S.A.* **2024**, *121*, e2401175121.

[27] B. Zhang, F. Liu, C. Nie, Y. Hou, M. Tong, *J. Hazard. Mater.* **2022**, *435*, 128966.
